# Benchmarking Structures and UV–Vis Spectra of Iron Complexes Against Experimental Data

**DOI:** 10.1021/acs.jpca.5c06391

**Published:** 2025-10-29

**Authors:** Renan R. Bertoloni, Vania M. Ramos, Ana Paula de Lima Batista, Antonio G. S. de Oliveira-Filho

**Affiliations:** ‡ Departamento de Química, Faculdade de Filosofia, Ciências e Letras de Ribeirão Preto, 124588 Universidade de São Paulo, 14040-901 Ribeirão Preto, SP, Brazil; § Departamento de Química, Grupo Computacional de Catálise e Espectroscopia (GCCE), 67828 Universidade Federal de São Carlos (UFSCar), 13565-905 São Carlos, SP, Brazil; ∥ Instituto de Química de São Carlos, 201361 Universidade de São Paulo, 13566-590 São Carlos, SP, Brazil

## Abstract

This benchmark study focuses on the evaluation of theoretical methodologies for geometry and ultraviolet–visible (UV–vis) spectral prediction of mononuclear iron coordination complexes. For this purpose, 17 structurally diverse iron complexes with experimentally determined X-ray structures and UV–vis absorption spectra were selected from the literature. For ground-state geometry, different computational approaches were evaluated: GFN1-xTB, BP86­(D4), PBE­(D4), revPBE­(D4), OPBE­(D4), TPSS­(D4), r^2^SCAN, B97­(D4), B3LYP/G­(D4), TPSSh­(D4), MN15, revM11, ωB97X­(D4), HF-3c, r^2^SCAN-3c, and PBEh-3c. The meta-hybrid functional TPSSh­(D4) delivers the best performance, establishing it as the preferred method for geometry optimizations of iron coordination complexes. For the prediction of UV–vis absorption spectra, time-dependent density functional theory (TD-DFT) calculations were performed on the optimized structures, at the TPSSh­(D4)/def2-TZVP/CPCM level of theory, using 13 density functionals (TPSS, r^2^SCAN, revM06-L, TPSSh, O3LYP, B97, B3LYP/G, PBE0, MN15, revM11, ωPBE, CAM-B3LYP and ωB97X). The functionals were ranked based on their ability to reproduce both the excitation energies and the overall spectral shape of the experimental spectra after using optimized Gaussian broadening and energy shifts on the calculated spectra. The hybrid functional O3LYP provided the most accurate excitation energies, with the lowest average energy shift, while the meta-GGA functional revM06-L demonstrated exceptional performance for reproducing the spectral shape, with the highest median similarity to the experimental spectra.

## Graphical abstract

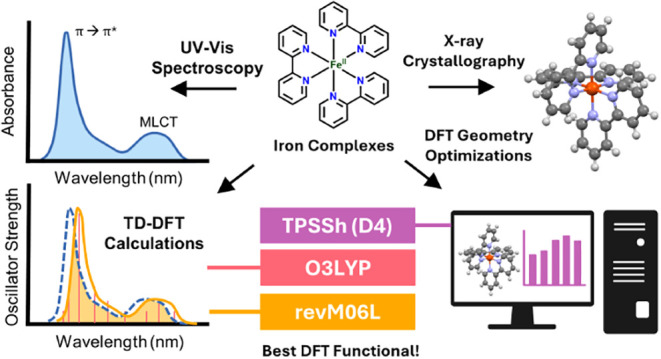

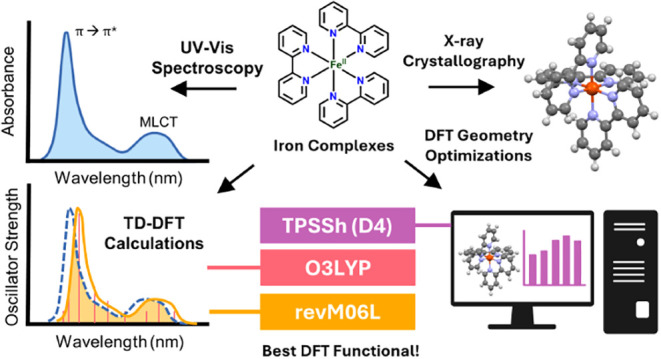

## 1 Introduction

The properties of transition metals arise from their d-shell electrons, which enable variable oxidation states, diverse chemical reactivity due to the formation of coordination complexes with an enormous number of ligands, and distinct physical, electronic, and magnetic properties.^1–9^ Iron, in particular, is the most abundant in the Earth’s crust by mass (approximately 6%) and the cheapest among transition metals.^10,11^ Biologically, iron is essential to all high forms of life due to its participation in the biocatalysis of iron-dependent enzymes, with ubiquitous involvement in redox processes.^12^ Chemically, iron is a multifaceted element that supports formal oxidation states ranging from *–*II to VII.^13–15^ Its multifunctionality enables broad application across areas such as (photo)­catalysis,^16–19^ supercapacitors,^20^ and metallopharmaceuticals,^21,22^ making iron an excellent candidate to replace precious metals in emerging applications.

Computational and data-driven approaches are crucial for exploring and realizing the potential of new compounds in novel applications.^23^ Kohn–Sham density functional theory (KS-DFT) and its time-dependent version (TD-DFT) are the workhorses of computational chemistry for exploring the structure, reactivity, electronic and optical properties of molecules and materials, widely applied to coordination and organometallic chemistry.^24–29^ Although DFT functionals provide computationally efficient and reasonably accurate solutions to the electronic Schrödinger equation, they have inherent limitations, such as delocalization errors, self-interaction inaccuracies, inadequate treatment of dispersion interactions, and the lack of a systematic hierarchy, which can make DFT (and TD-DFT) calculations occasionally fail in unexpected ways.^30–34^ Additionally, transition metal chemistry can be particularly difficult for such calculations due to their multiconfigurational nature, open-shell configurations, and strong electron correlation effects.^27,35–37^ Rigorous benchmarking against experimental or high-level theoretical reference data is necessary to address these shortcomings, ensuring reliability and the usage of a suitable DFT functional for a given task. Data reliability and accuracy are critical factors for robust computational and data science applications in chemistry.^38^

Determining ground-state properties, such as molecular structure, is fundamental to understanding chemical systems. Numerous benchmark studies have compared DFT-derived geometries to experimental data and/or high-level computational results for specific sets of compounds.^39–44^ High-level calculations have the disadvantage of high computational cost, making it unfeasible to carry out benchmarks that contain larger molecules, such as most coordination compounds. As for studies using experimental data, the limitation lies in the choice of compounds that have already been synthesized with published crystal structures. For instance, Aoto et al. demonstrated that the choice of reference data, between experimental or high-level computations, did not significantly change the relative performance ranking of DFT functionals.^40^ Therefore, the choice of reference data in a benchmark should consider that high-level calculations, while broadly applicable, incur prohibitive computational costs for large systems like coordination compounds, especially when in complex environments.

For transition metal benchmarks, what is observed in these studies and justifies benchmarking different types of systems is that higher rungs in Jacob’s ladder do not necessarily deliver more robust results, since hybrid methods like M06, TPSSh, and B3LYP tend to give the best results for molecular structure calculations.^39,45^ Tight-binding methods, such as GFN2-xTB, are a promising alternative for molecular structure calculations due to their low computational cost.^42^ However, individual analyses for coordination compounds are still scarce and, given the difficulty in ranking DFT functionals, are indispensable when it comes to studying this type of compound.

Regarding excitation energies, several papers have been published to verify the accuracy of TD-DFT methods for various types of systems, using the calculation of electronic spectra to check the performance of this approach.^46–51^ The biggest challenge encountered in the computational determination of a ultraviolet–visible (UV–vis) spectrum from calculated excited-state properties is that these values cannot be compared directly with the experimental data, but rather depend on models for analyzing experimental UV–vis spectra that fail to take into account all the experimental conditions and the band broadening generated by these conditions.^46,52^ An alternative approach to this problem is to directly compare the excited-state properties calculated using TD-DFT with those calculated using high-level theory.^53–56^ However, this approach becomes more challenging as the systems studied increase in size and complexity, such as coordination complexes, making indirect comparison with experimental UV–vis spectra the most viable method to carry out TD-DFT benchmarks for this class of compounds.^28,29,57–59^ One of the main limitations of TD-DFT, concerning the calculation of excitation energies, is the underestimation of charge transfer excitations, which can be challenging for metal–ligand charge transfer (MLCT) transitions.^50,60,61^ Typically, range-separated functionals are employed to overcome such limitations,^49^ but the literature lacks information on the performance of various types of functionals in predicting the UV–vis spectra of coordination complexes.

Other challenge to overcome in this type of study is the use of a consistent quantitative metric for analyzing the error associated with TD-DFT functionals when compared with experimental data.^46,50^ Usually, the relative error for a specific excitation energy is calculated, or the profile of the two spectra is qualitatively compared (shifting the theoretical spectra as necessary).^29,54,62,63^ None of these methods is quantitatively satisfactory when ranking the best DFT functionals to describe excited electronic states. Therefore, there is an effort to determine the best way to perform functional ranking for TD-DFT calculations.^46,50,64,65^ Fehér et al. developed a method for analyzing the complete spectral shape and excitation energies that uses a sum of Gaussians, depending on two parameters associated with the bandwidth and a linear wavelength scaling factor, to obtain the full absorption spectrum from excitation energies and oscillator strengths.^51^ The same procedure was applied to a transition-metal benchmark, with representation of the Cu, Ru, Ir, Fe, Au, Mo, and W elements.^59^ Among all studied complexes, iron complexes seem to be the most problematic ones, indicating that further investigations may be needed for this specific set of complexes.

In this work, we undertook a benchmark on a diverse data set of Fe complexes to (i) determine the best computational model for the molecular structure of Fe coordination complexes among the selected functionals and methods and (ii) employ a quantitative ranking analysis based on both spectral shape and excitation energies to select the most suitable TD-DFT functionals for predicting the UV–vis spectra of iron complexes. To ensure the diversity of the data set, we selected experimental data that represent a wide range of possibilities, including variations in oxidation state, geometry, and class of ligands present in the complexes, with a focus on mononuclear ones. This systematic benchmark addresses a critical gap by rigorously quantifying the performance of computational methods for iron coordination complexes, serving as an essential guide for selecting methodologies in future studies of analogous compounds.

## 2 Experimental Reference Values

To assess the performance of computational models for the structure and electronic spectrum, a database of experimentally determined reference values for crystallographic structures and UV–vis spectra was compiled. It consists of 15 iron coordination complexes and 2 organometallic compounds ranging in size from 11 to 67 atoms, with tetrahedral, trigonal bipyramidal, and octahedral geometries, in coordination numbers 4, 5, and 6, in formal oxidation state 0 to IV, in charge from 2– to 2+, and in spin multiplicity from 1 to 6. The lowest energy spin state was considered based on the information available in the respective references for each compound.

The crystallographic data for all studied compounds were obtained from the Cambridge Structure Database (CSD).^66^ Counterions, solvent molecules, and other extraneous structures present in the crystalline structure were excluded to focus the subsequent modeling and analysis solely on the metal complex.

The experimental UV–vis spectra were obtained from the respective references for each compound studied. The spectra were digitized using the PlotDigitizer application.^67^ To allow a direct comparison between the experimental and computed spectra, the experimental spectra were converted from units of wavelength to energy units using the Jacobian transformation factor (*hc*/*E* ^2^) to scale the intensity.^68^ After converting, the spectra were smoothed and interpolated to have a 100 cm^–1^ (∼0.0124 eV) interval between the points. In cases where necessary, the window range of the experimental spectra was narrowed to a smaller region to minimize spectral noise.

Figure 1 illustrates the representative structural formulas for all 17 complexes studied in this work, along with the numbering used to label them. Table 1 shows the numbering of every complex studied in this work, together with the reference works, the medium in which the experimental UV–vis spectrum was obtained, and the spectral range used for similarity calculations (for names and the spin multiplicity of each compound, see Table S1).

**Figure 1:**
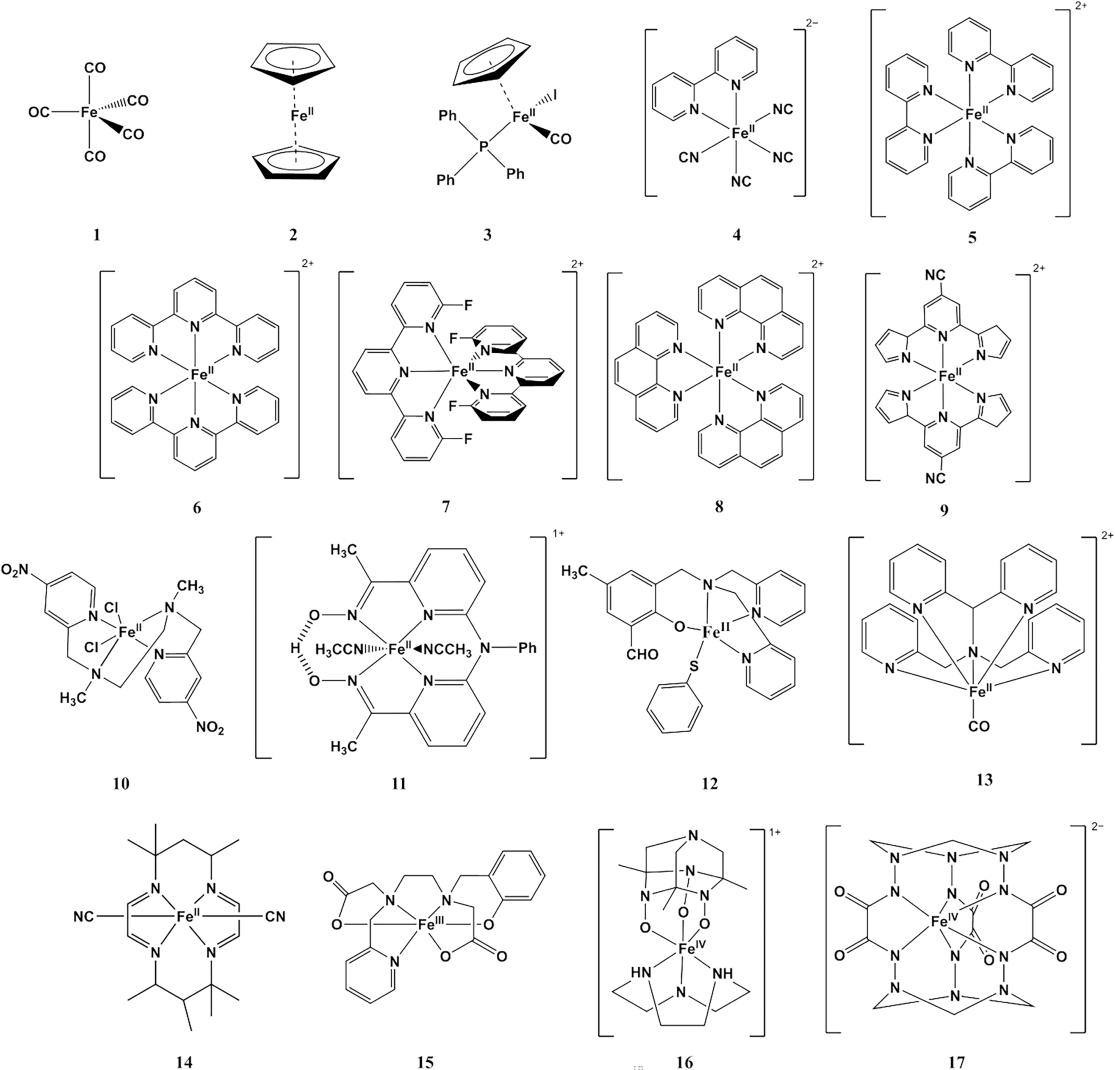
Structural formulas of the iron complexes investigated in this work, labeled 1–17.

**Table 1:** Number, Medium in Which the UV–Vis Spectrum Was Recorded, Spectral Range, and Respective References for All the Compounds Studied in This Work

compound	medium	spectral range (nm)	spectral range (eV)	refs
1	gaseous	200–354	3.5–6.2	^69^
2	isopentane	182–620	2.0–6.8	^70–72^
3	dimethyl sulfoxide	264–827	1.5–4.7	^73^
4	acetonitrile	258–886	1.4–4.8	^74,75^
5	acetonitrile	258–886	1.4–4.8	^74,76^
6	acetonitrile	243–689	1.8–5.1	^77,78^
7	acetonitrile	248–590	2.1–5.0	^79^
8	water	248–653	1.9–5.0	^80,81^
9	acetonitrile	230–827	1.5–5.4	^82^
10	acetonitrile	302–886	1.4–4.1	^83^
11	acetonitrile	264–620	2.0–4.7	^84^
12	dichloromethane	326–827	1.5–3.8	^85^
13	water	288–539	2.3–4.3	^86^
14	dichloromethane	248–886	1.4–5.0	^87^
15	water	282–729	1.7–4.4	^88^
16	methanol	288–827	1.5–4.3	^89^
17	water	276–827	1.5–4.5	^90^

## 3 Computational Details

### 3.1 Electronic Ground State Geometries

All electronic structure calculations were performed using the Orca program (version 5.0.4),^91–94^ applying the standard Resolution of Identity (RI) approximation for Coulomb integrals (RI-J)^94^ and COSX numerical integration for HF exchange.^95^ The molecular geometry of each complex was optimized employing 16 methods, including the tight-binding DFT (GFN1-xTB^96^), composite methods (HF-3c,^97^ PBEh-3c,^98^ r^2^SCAN-3c^99^), generalized gradient approximation, GGA, (BP86­(D4),^100,101^ PBE­(D4),^102^ revPBE­(D4),^103^ OPBE­(D4)^102,104^ ), meta-GGA (TPSS­(D4),^105^ r^2^SCAN^106^), hybrid-GGA (B97­(D4),^107^ B3LYP/G­(D4)^108,109^ ), hybrid meta-GGA (TPSSh­(D4),^105,110^ MN15 ^111^) and range-separated hybrid (revM11,^112^ ωB97X­(D4)^113^), with the def2-TZVP^114,115^ basis set. The Grimme dispersion correction (D4)^116,117^ was used in all methods that do not account for dispersion forces explicitly. To confirm the nature of the optimized geometries as true minima on the potential energy surface, vibrational frequency analyses were carried out. The absence of imaginary frequencies confirmed that all optimized structures were a local minima.

To compare the optimized structures produced by different computational methods with the experimentally determined structure obtained from X-ray diffraction data, we used the following metrics: root-mean-square error (RMSE), mean unsigned error (MUE), and mean signed error (MSE). The RMSE,^118,119^ as defined in eq 1, was used to quantify the deviation between the optimally superposed Cartesian coordinates of the computationally and experimentally derived structures.

1 $$\mathrm{RMSE}=\sqrt{\frac{1}{N}\sum_{i}^{N}\sum_{\gamma =x,y,z}{\left(\gamma_{i}^{\mathrm{comp}}-\gamma_{i}^{\mathrm{exptl}}\right)}^{2}}$$

The MUE and MSE, as defined in eqs 2 and 3, respectively, were used to quantify deviations between computationally and experimentally derived bond distances involving the metallic center, i.e., bonds of the type Fe–X, where X is an atom directly coordinated to iron.

2 $$\mathrm{MUE}=\frac{1}{n}\sum_{X}\left|R_{\mathrm{FeX}}^{\mathrm{comp}}-R_{\mathrm{FeX}}^{\mathrm{exptl}}\right|$$

3 $$\mathrm{MSE}=\frac{1}{n}\sum_{X}\left(R_{\mathrm{FeX}}^{\mathrm{comp}}-R_{\mathrm{FeX}}^{\mathrm{exptl}}\right)$$

where *n* is the number of FeX bonds and *R*_FeX_^comp^ and *R*_FeX_^exptl^ are the computationally and experimentally derived bond distances, respectively. Hydrogen atoms were excluded from the geometry/bond error analysis due to the challenges in accurately determining their positions from weak scattering signals in conventional X-ray diffraction experiments, particularly in the presence of heavier elements.^120^

During the benchmarking analysis of the ground state molecular geometry, all the structure optimizations were first performed for the isolated molecules in vacuum. Once the best density functional was selected (TPSSh­(D4), see Section 4.1), the geometry of each complex was reoptimized using the conductor-like polarizable continuum model (C-PCM)^121,122^ to account for solvation effects. These calculations are referred as DFT/TPSSh­(D4)/def2-TZVP/CPCM­(solvent). The inclusion of solvent effects is essential, as most experimental UV–vis spectra used as reference data in this study were recorded in solution. The C-PCM parameters of the solvent in which the experimental UV–vis spectrum was recorded is listed in Table 1.

### 3.2 TD-DFT Calculations and UV–Vis Absorption Spectra Analysis

In order to ensure methodological consistency with previous studies and facilitate reproducibility, the UV–vis absorption profiles were calculated by TD–DFT employing the Tamm–Dancoff approximation, which is widely used due to its favorable balance between computational efficiency and accuracy,^123^ using the def2-TZVP^114,115^ basis set and a selection of functionals: meta-GGA (TPSS,^105^ r^2^SCAN,^106^ revM06-L^124^), global hybrids (TPSSh,^105,110^ O3LYP,^104^ B97,^107^ B3LYP/G,^108,109^ PBE0,^125^ MN15 ^111^), and range-separated hybrids (revM11,^112^ ωPBE,^126^ CAM-B3LYP,^127^ ωB97X^113^). TD-DFT calculation for 40 electronic states was performed as a single-point calculation on the electronic ground state geometry of each complex, optimized at the DFT/TPSSh­(D4)/def2-TZVP/C-PCM­(solvent) level of theory (See Section 4.1 and Table 1 for the solvents).

All the TD-DFT calculations also employed the implicit solvent model C-PCM with the same solvents used in the experimental reference spectra (Table 1).

The computed spectra were compared to the experimental data to assess the performance of the density functions used in this work. This comparison is based on the overall similarity between the shape (relative spectral intensity as a function of energy) of the experimental and computed spectra. In order to do that, we assume that the electronic spectra can be obtained from the calculated TD-DFT vertical transition energies and oscillator strengths by applying Gaussian broadening, i.e., the overall spectrum, *I* ^comp^(*E*), is a sum of Gaussian functions

4 $$I^{\mathrm{comp}}\left(E;\left\{f_{i},E_{i}\right\},\delta ,\sigma \right)=N\sum_{i}f_{i}\exp\left(-\frac{1}{2}\frac{{\left(E-E_{i}+\delta \right)}^{2}}{\sigma^{2}}\right)$$

where *N* is a constant that normalizes the maximum intensity of the spectra to one, i.e., *N* = 1/max_*E*_ (*I*(*E*)), *f*_*i*_ and *E*_*i*_ are, respectively, the oscillator strengths and transition energies of a given electronic transition *i* derived from the TD-DFT calculation, and δ and σ are, respectively, the shift in the transition energies and the broadening parameters that are uniformly applied for all computed transitions. From eq 4, one sees that the shape of the computed spectra is (i) parametrically dependent on the set of transition energies and oscillator strengths, {*f*_*i*_, *E*_*i*_}, (determined by the level of theory); (ii) the width of the Gaussian functions, σ, that is related to the full width at half-maximum, FWHM, (FWHM ≈ 2.355σ), and, (iii) the energy shift, δ.

The similarity factor, *S*, between the experimental (*I* ^exptl^(*E*)) and the computed (*I* ^comp^(*E*)) spectra over a given energy range *E*_1_ to *E*_2_ can be quantified as

5 $$S\left(\delta ,\sigma \right)=\frac{\int_{E_{1}}^{E_{2}}I^{\mathrm{exptl}}\left(E\right)I^{\mathrm{comp}}\left(E\right)dE}{\sqrt{\int_{E_{1}}^{E_{2}}{\left[I^{\mathrm{exptl}}\left(E\right)\right]}^{2}dE\int_{E_{1}}^{E_{2}}{\left[I^{\mathrm{comp}}\left(E\right)\right]}^{2}dE}}$$

which is based on cosine similarity and was used for comparing spectra in refs 64,128–130 . The numerical integration was performed using Simpson’s rule.^131^ Given that for each level of theory, the similarity between computed and experimental spectra is a function of both the energy shift and the broadening, these two parameters were optimized (eq 6) in order to maximize *S* using the Nelder–Mead algorithm.^132^

6 $$S^{\max}=\max_{\delta ,\sigma }S\left(\delta ,\sigma \right)$$

To avoid meaningless values of these parameters, δ was constrained within a range of −1.5 to 1.5 eV, while σ varied between 0 and 0.5 eV.

As a result of the analysis expressed in eq 5, a given level of theory can be evaluated in terms of the maximum similarity *S* ^max^, a value between 0 and 1 (100%), between the experimental spectrum and the calculated one for optimal energy shift and broadening. The optimal energy shift can also be used to evaluate a given level of theory in relation to the apparent average error in the calculated transition energies.

## 4 Results and Discussion

### 4.1 Ground Electronic State Geometries

To discuss the quantitative evaluation of the molecular geometry of the Fe complexes obtained by geometry optimization at a given level of theory, we mainly based on the RMSE, MUE and MSE averaged over all the 17 compounds, Figure 2. In this case, they are referred as average RMSE, average MUE and average MSE, respectively. For some cases (see below), we also discuss an error metric for an individual molecule, and all values can be found in SI.

**Figure 2:**
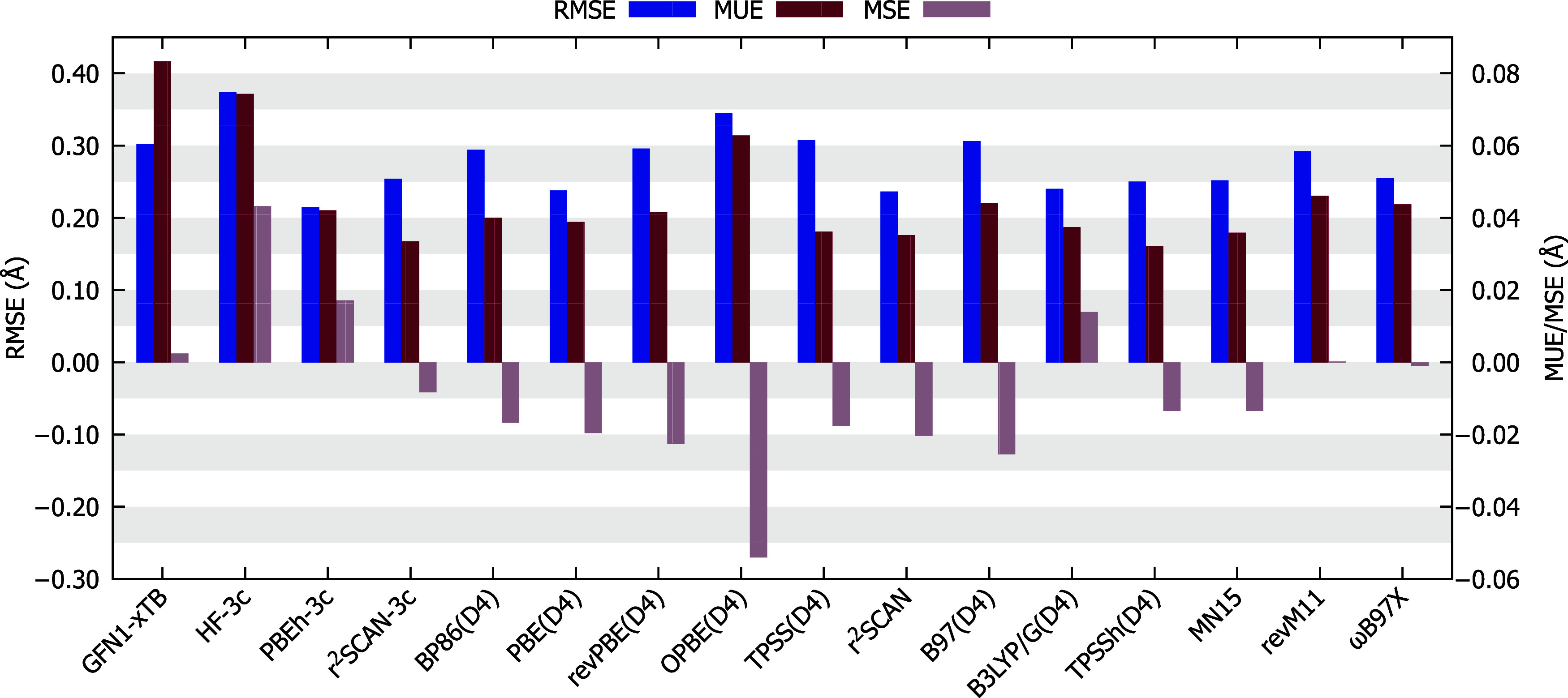
Average root-mean square error (RMSE), average mean unsigned error (MUE), and average mean signed error (MSE) obtained for every method studied in this work. For the GGA, meta-GGA, hybrids, and ωB97X­(D4) functionals, the def2-TZVP basis was used.

The seven best performances were presented, in ascending order of average RMSE, by PBEh-3c, r^2^SCAN, PBE­(D4), B3LYP/G­(D4), TPSSh­(D4), MN15, and r^2^SCAN-3c methods. The highly efficient and practical PBEh-3c method yielded the lowest average RMSE value of 0.2142 Å. The meta-GGA functionals PBE and r^2^SCAN also demonstrated good performance, with favorable computational costs, and are worthy of highlighting, yielding average RMSE values of 0.2357 and 0.2374 Å, respectively.

The seven worst performances were presented, in ascending order of average RMSE, by BP86­(D4), revPBE­(D4), GFN1-xTB, B97­(D4), TPSS­(D4), OPBE­(D4) and HF-3c. HF-3c obtained the highest average RMSE value of 0.3736 Å. The GFN1-xTB tight-binding method was also one of the worst performers, with an average RMSE of 0.3017 Å. However, this value is still lower than that found by Nurhuda et al.^133^ in their study of the performance of this method for metal–organic frameworks geometry optimization (0.489 Å), indicating a better performance when the metal elements are in the form of coordination complexes. Once the difference in the average RMSE between the semiempirical GFN1-xTB and the best performing density functionals is of the order of 0.1 Å, this method can be used as a starting point for the optimization of molecular geometries for iron complexes, that can be further refined using PBEh-3c and/or other of the top performing methods, in the same way that was suggested by Vuckovic and Burke,^134^ based on a different metric for a data set of main-group molecules. The ωB97X and revM11 functionals showed average performance, with mean RMSE values of 0.2547 and 0.2917 Å, respectively. Considering that both are range-separated, these results do not justify the high computational cost, and therefore, these functionals are not efficient for molecular geometries of Fe coordination complexes. No correlation was observed between the errors obtained and the oxidation number of the metal center, indicating that this is not a significant factor when choosing a methodology for these calculations.

Compounds 2, 3, and 15 had RMSE values above 0.3 Å, while compound 12 had an exceptionally high value of 1.4187 Å (Figure 3). These higher RMSE values can be justified on the basis of the type of ligand present in the structure. In the case of compounds 2 and 3, the cyclopentadienyl anion can present many conformers due to rotation of its structure, which also occurs with the triphenylphosphine ligand in compound 3, the thiophenolate in compound 12, and with the multiple single bonds present in compound 15. In a recent study, Fomsbee et al.^135^ demonstrated that the presence of only one freely rotating bond can lead to an increase in the RMSE value obtained for optimized geometries, which explains the observed values for these compounds. Although RMSE is widely used to evaluate the efficacy of computational methods in predicting molecular geometries based on overlap with experimental geometries, due to the conformational flexibility introduced by freely rotating bonds, RMSE may provide a misleading assessment of methodological performance and it should therefore be interpreted with caution in the context of this study. Another limitation of RMSE analysis is its strong dependence on system size, as it scales with the number of atoms and disregards underlying chemical information.^136,137^ Consequently, alternative approaches for analyzing and comparing molecular structures should also be considered.

**Figure 3:**
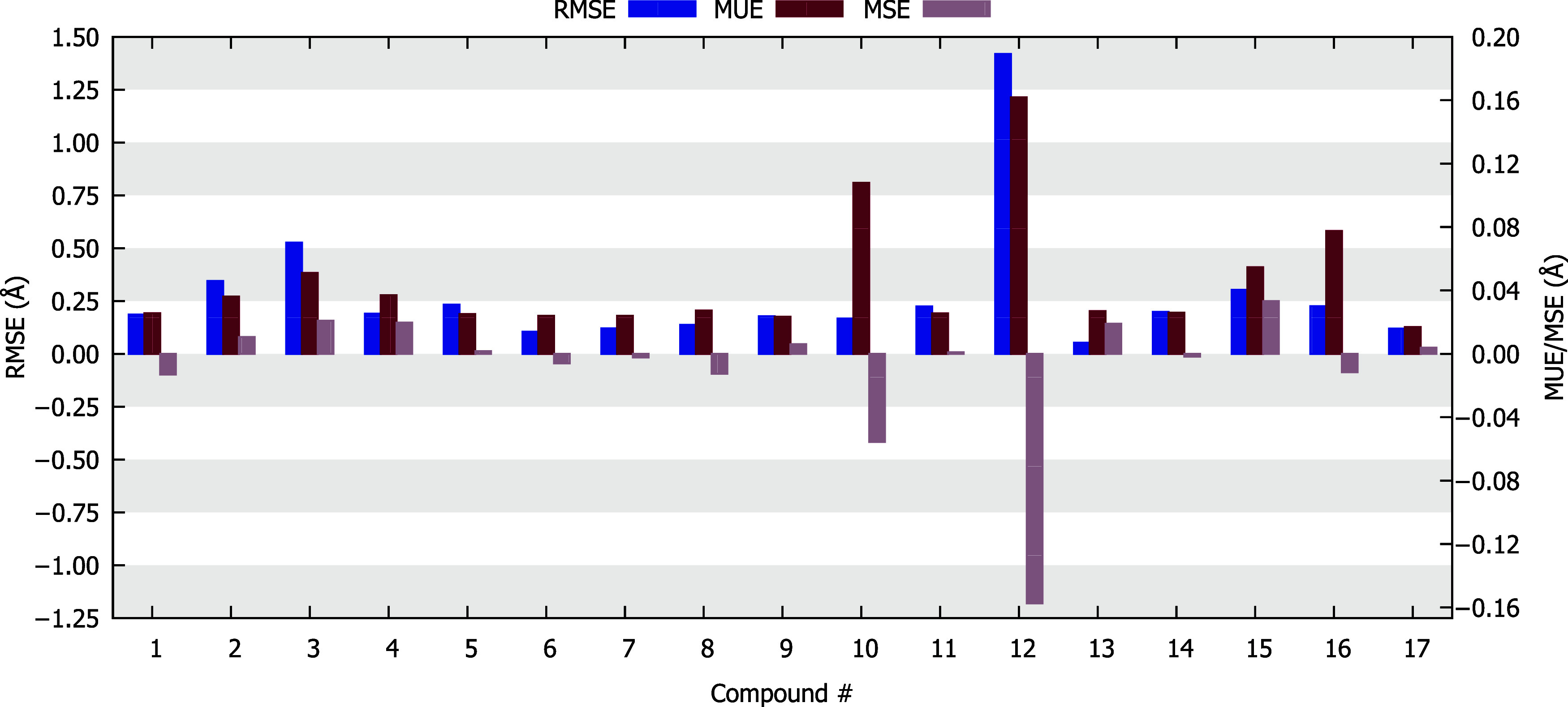
Average root-mean square error (RMSE), mean unsigned error (MUE), and mean signed error (MSE) obtained for every compound studied in this work. For the GGA, meta-GGA, hybrids, and ωB97X­(D4) functionals, the def2-TZVP basis was used.

According to ligand field theory, the atoms coordinated directly to the metal center are the most important for determining important factors such as molecular geometry, electronic density of the metal center, and ligand lability.^138^ Therefore, we analyze the average MUE and MSE values for the bonds involving the metal center. In this analysis, the seven best performances were presented, in ascending order of average MUE, by the TPSSh­(D4), r^2^SCAN-3c, r^2^SCAN, MN15, TPSS­(D4), B3LYP/G­(D4), and PBE­(D4) methods. TPSSh­(D4) was the method that obtained the lowest average MUE value of 0.0321 Å. The seven worst performances were presented, in ascending order of average MUE, by the PBEh-3c, ωB97X­(D4), B97­(D4), revM11, OPBE­(D4), HF-3c, and GFN1-xTB. The tight-binding GFN1-xTB again showed poor performance, returning an average MUE value of 0.0832 Å, which is more than twice the value for TPSSh. Among the composite methods, HF-3c again performed poorly, with an average MUE of 0.0742 Å, whereas r^2^SCAN-3c performed well, with an average MUE of only 0.0334 Å, which is very close to the best method. GGA and meta-GGA methods demonstrated an average performance, characterized primarily by the BP86 and revPBE with average MUE values of 0.0399 and 0.0415 Å, respectively. These two values lie between the seven best and the seven worst performances. The only exception to this is OPBE, which showed a high average MUE of 0.0627 Å. The hybrid methods again performed better, with a notable highlight being the best performance of TPSSh (0.0321 Å) and the lowest median MUE value of MN15 (0.0164 Å), ωB97X had a slightly better performance, having an average value of 0.0436 Å. However, this value is still too high for the computational cost of a range-separated hybrid functional. It should be noted that the TPSSh functional was selected as the best for optimizing the geometry of Fe complexes not because it outperformed all other functionals in every individual case, but because it yielded the lowest average MUE in this study. This functional ranked among the top eight for 15 of the 17 molecules analyzed and showed the best performance overall for molecule 17. These values indicate that, in a generalized way, and with the goal of selecting a functional that tends to avoid large deviations in the geometry optimization of iron complexes, TPSSh is the most suitable functional for this purpose.

A nice way to visualize the relative performance of these functionals is through a box plot, as shown in Figure 4. This allows us for a clear assessment of the inferior performance of GFN1-xTB, HF-3c, and OPBE compared to the other studied functionals. It also highlights the excellent performance of TPSSh, as well as that of r^2^SCAN-3c, r^2^SCAN, and MN15, which showed a lower median value of MUE, even when compared to TPSSh.

**Figure 4:**
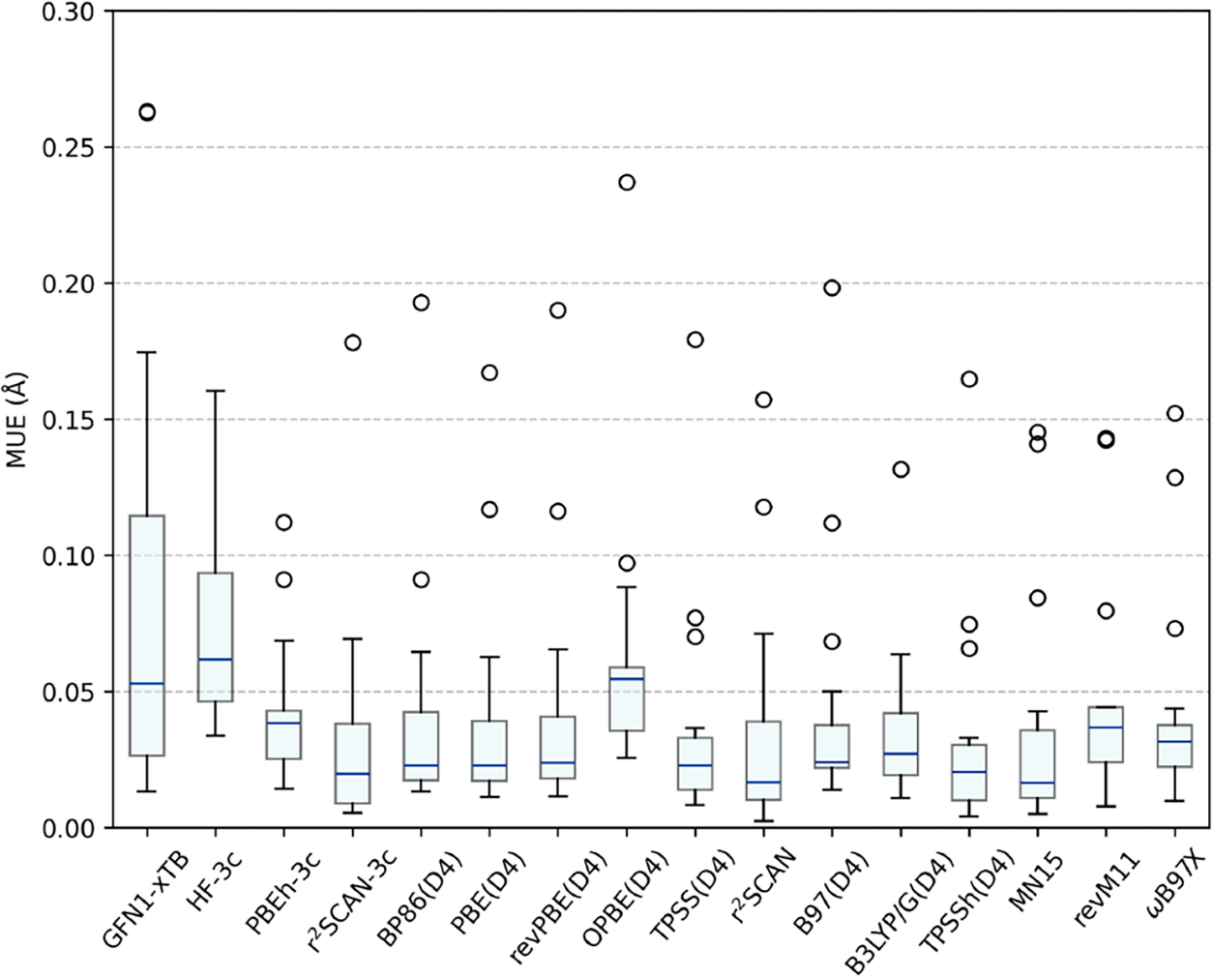
Box plot of mean unsigned error (MUE) values obtained for every method studied in this work. The whiskers represent the minimum and maximum values, excluding outliers. The lowest and highest lines of the blue rectangle represent the first and third quartiles, respectively. There are exactly 25% of the points that are less than the first quartile and exactly 25% of the points that are more than the third quartile, excluding outliers. The blue line represents the median value. The circles are the outliers.

However, it is important to make a distinction regarding the complexes studied. Among the 17 structures studied, 13 contain Fe in II oxidation state. To better understand the role of this oxidation state in the obtained results, the same box plot was constructed considering two different groups: the first comprising the 13 Fe­(II) compounds, and the second including the remaining four compounds. These plots are shown in Figure 5. As expected, the trend of TPSSh yielding the lowest average error remained for the first group, whereas for the second group, this functional exhibited the second-lowest average, surpassed only by its meta-GGA counterpart, the TPSS functional. Although the second group contains far fewer molecules and thus does not accurately represent Fe complexes in other oxidation states, this analysis reinforces that the TPSS and TPSSh functional families are excellent choices for Fe geometry optimizations in a general context.

**Figure 5:**
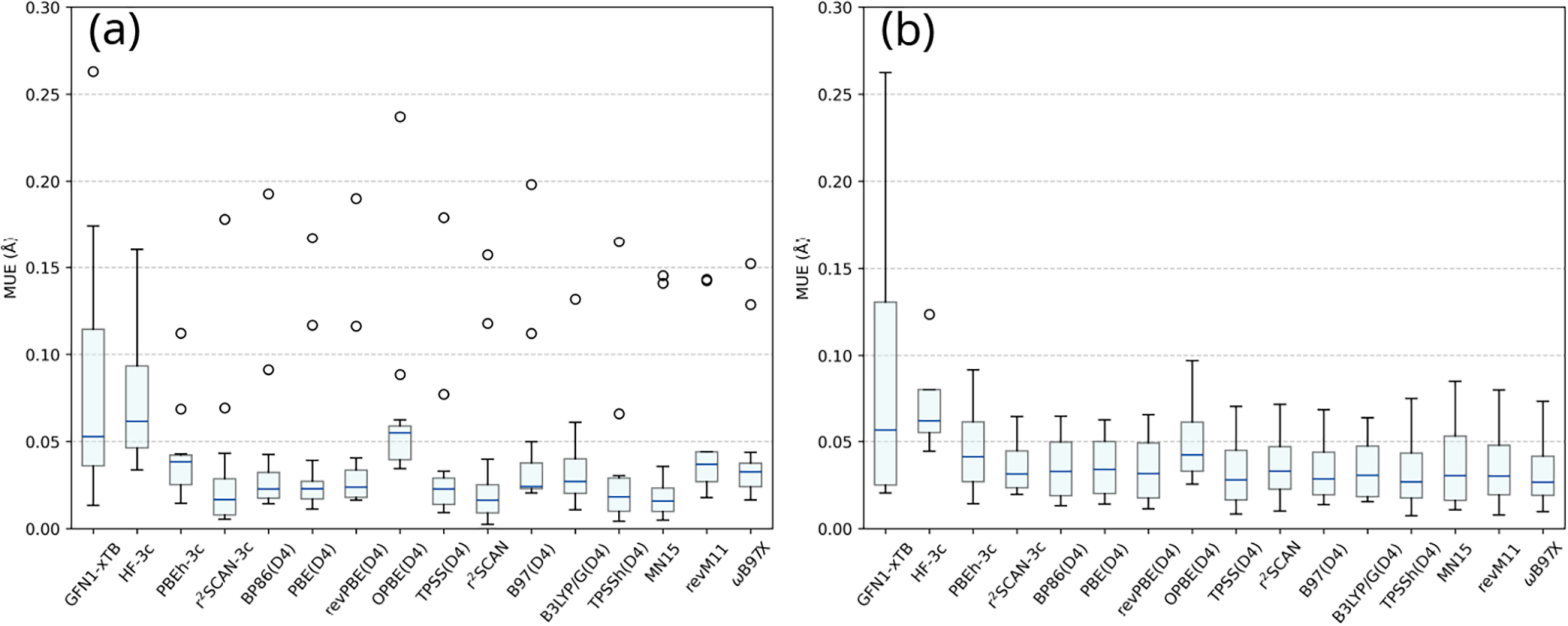
Box plot of mean unsigned error (MUE) values obtained for (a) all Fe­(II) compounds (2–14) and (b) compounds 1, 15, 16, and 17. The whiskers represent the minimum and maximum values, excluding outliers. The lowest and highest lines of the blue rectangle represent the first and third quartiles, respectively. There are exactly 25% of the points that are less than the first quartile and exactly 25% of the points that are more than the third quartile, excluding outliers. The blue line represents the median value. The circles are the outliers.

In the MUE analysis, compound 12 again showed an exceptionally high MUE value (0.1618 Å) together with compound 10 (0.1079 Å). Compound 10 has a greater number of single bonds in its ligands, resulting in an increased number of conformers However, in this case, the atoms participating in these simple bonds are directly linked to the metal center, which reflects in a higher MUE value. MSE analysis for compound 12 shows that all analyzed bonds are being underestimated. Figure 6 shows the calculated unsigned error for every M-L bond individually in compound 12. For this compound, HF-3c returned the lowest errors and OPBE the highest. For all the methods, the highest error is associated with the Fe–N bonds, indicating that the presence of single bonds between the N atoms and atoms other than the metal center makes the position of this atom more difficult to calculate with good precision. A second factor that may have made compound 12 the most pathological is its molecular geometry, of the trigonal bipyramid type. This geometry may be favored by steric hindrance caused by the sulfur atom present in one of the ligands, disfavoring the common octahedral geometry for Fe­(II) complexes due to the greater proximity of the ligands. The electronic structure methods used may have greater difficulty in capturing this effect, resulting in significant distortions in the geometry of this complex, which can cause an increase in MUE. This example is crucial for determining the limitations of DFT-based methods in determining molecular geometries of coordination complexes, especially those with atypical geometries.

**Figure 6:**
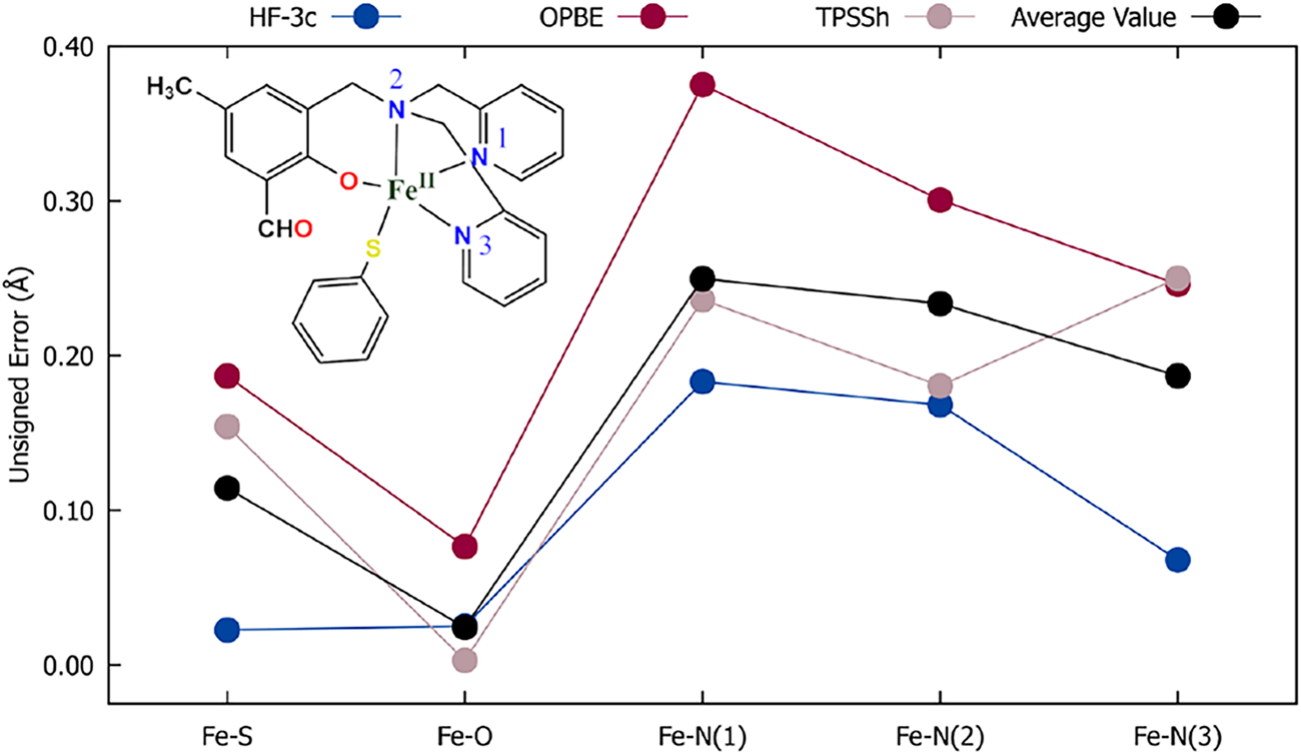
Individual unsigned error (|*R*_*i*_(Theor.) – *R*_*i*_(Exptl.)|) values for all the metal–ligand bonds in molecule 12 using the OPBE­(D4)/def2-TZVP (blue), HF-3c (red), TPSSh­(D4)/def2-TZVP (beige) methods, and its average value (black).

Overall, the analysis of the geometries reveals that the PBEh-3c method is most accurate in terms of RMSE, whereas the TPSSh functional offers the smallest deviations when considering MUE. For the next stage of the work, TPSSh was chosen to carry out the solvent optimizations because of two main factors. The first is that MUE values are the primary factor in determining the accuracy of a method, without the influence of errors associated with the difference in position of atoms far from the metal center, and consequently, they are less significant for the chemical properties of this complex. The second is that, comparing the RMSE and MUE values for both methods, the MUE of PBEh-3c falls in the middle, while the RMSE of the TPSSh functional is among the lowest, indicating that the TPSSh method is more accurate in general. Previous works have demonstrated that TPSSh is highly effective in calculating the ground-state electronic geometries and energies of first- and second-row transition metals.^39,44,139^

An important aspect in the quantitative analysis of the performance of computational methods for predicting molecular geometries is having a meaningful target, something analogous to the concept of chemical accuracy (1 kcal/mol) as proposed by Pople for thermochemical measurements.^140^ DeYonker et al. proposed that for transition metal compounds, given their complex electronic structure, a value of 3 kcal/mol should be used instead of the chemical accuracy typically employed for main-group chemistry.^141^ For equilibrium bond lengths, Peterson, Feller, and Dixon arbitrarily proposed the value of ±0.005 Å as chemical accuracy.^142^ As shown in Table 2, none of the methods investigated in this work reach the stringent target of ±0.005 Å proposed by Peterson et al., indicating that the target is too rigorous and/or the investigated systems are some what difficult to model accurately (presence of multireference character or unusual bonding) and/or the computational methods are in fact less accurate than the target. Therefore, in parallel with the work of DeYonker et al.,^141^ which proposes transition metal chemical accuracy three times greater than the one proposed by Pople, an arbitrary accuracy of ±0.02 Å for bond lengths seems a reasonable target for an accurate and robust method when applied to transition metal complexes. In this work, even with a relaxed target, none of the methods achieves a precision of 0.02 Å. However, as will be discussed in Section 4.2, the calculated electronic spectra (and possibly other properties) on these approximate molecular geometries can be considered as accurate when compared to experimental data, to some extent due to favorable error cancellations.

**Table 2:** Calculated Average and Median MUE Values for All Used Methods

method	average MUE (Å)	median MUE (Å)
TPSSh(D4)	0.0321	0.0203
r^2^SCAN-3c	0.0334	0.0196
r^2^SCAN	0.0351	0.0166
MN15	0.0357	0.0164
TPSS(D4)	0.0361	0.0227
B3LYP/G(D4)	0.0373	0.0271
PBE(D4)	0.0387	0.0228
BP86(D4)	0.0399	0.0227
revPBE(D4)	0.0415	0.0238
PBEh-3c	0.0419	0.0383
ωB97X(D4)	0.0436	0.0315
B97(D4)	0.0439	0.0241
revM11	0.0460	0.0369
OPBE(D4)	0.0627	0.0545
HF-3c	0.0742	0.0616
GFN1-xTB	0.0832	0.0528

### 4.2 TD-DFT Calculations

The shift, broadening, and similarity parameters were used to quantitatively evaluate the effectiveness of the functionals chosen for the TD-DFT calculation of mononuclear Fe complexes. After calculating the theoretical spectrum, a shift and broadening value were applied to the line spectrum to maximize the similarity between the theoretical and experimental spectra, resulting in the “optimized” spectrum.

Figure 7 shows both the calculated and optimized TD-DFT spectra in comparison with the UV–vis experimental spectrum for compound 5 ([Fe­(bpy)_3_]^2+^), as an illustrative example. In this case, the values obtained for the shift and broadening were −0.23 and 0.58 eV, respectively. These parameters yield a theoretical spectrum with a 95.5% similarity to the experimental one. The same two bands present in the experimental spectrum are also present in the theoretical one. The lower energy band corresponds to the metal–ligand charge transfer (MLCT) transition of the complex, while the higher energy band corresponds to an intraligand (IL).^74^ A larger error was observed in the MLCT band, which, despite being able to predict the shape of the band with some accuracy, the calculation of the energy of this transition showed a significantly greater error than when compared to the IL band. This difference in the ability to predict the bands is a direct reflection of the limitations of DFT. By nature, the MLCT transition involves a redistribution of charge in the molecule, where the electronic density is transferred from orbitals of greater metallic character to orbitals of greater ligand character, characterizing a charge transfer and therefore presenting one of the difficulties of traditional DFT. In general, this same pattern can be observed for most of the results, highlighting the difficulty of DFT in modeling this type of transition for the iron systems.

**Figure 7:**
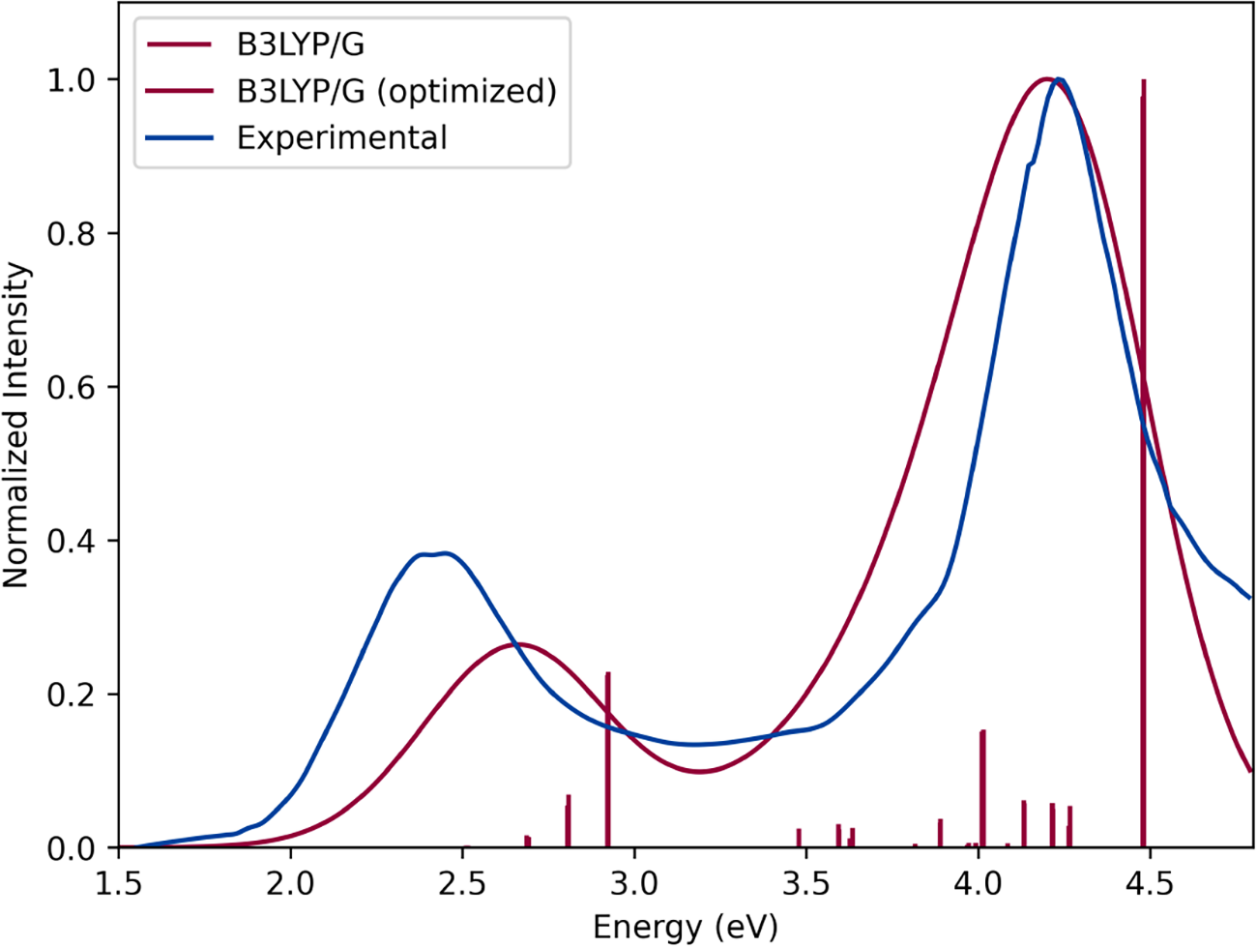
TD-DFT/B3LYP­(G)/def2-TZVP/CPCM­(acetonitrile)//DFT/TPSSh­(D4)/def2-TZVP/CPCM­(acetonitrile) calculated (red spikes) and optimized (red curve) spectra of compound 5 ([Fe­(bpy)_3_]^2+^) in comparison with experimental UV–vis data (blue curve).

The same procedure was applied to all systems, and the averaged results over all molecules are presented in Table 3 and illustrated in the boxplot format in Figure 8.

**Figure 8:**
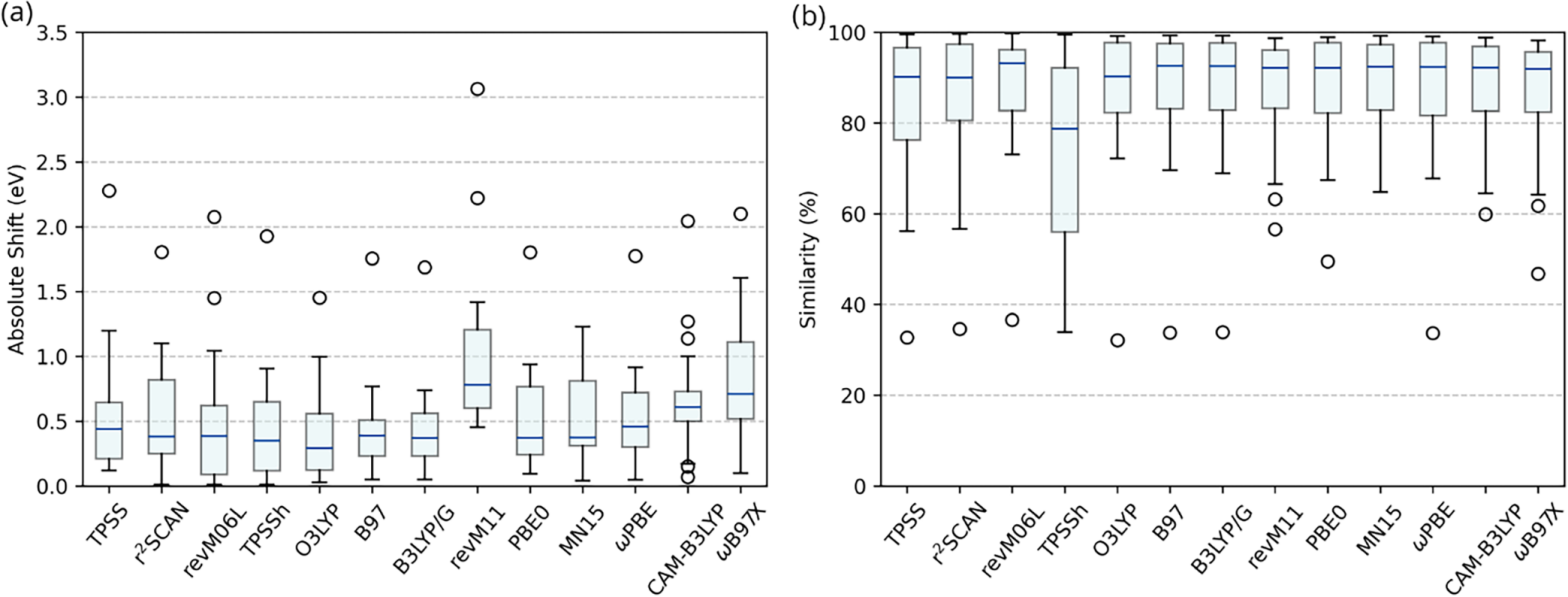
Box plot of absolute shift values, in eV (a) and average % similarity (b) obtained for every method in TD-DFT calculations. The whiskers represent the minimum and maximum values, excluding outliers. The lowest and highest lines of the blue rectangle represent the first and third quartiles, respectively. There are exactly 25% of the points that are less than the first quartile and exactly 25% of the points that are more than the third quartile, excluding outliers. The blue line represents the median value. The white circles are outliers.

**Table 3:** Calculated Average and Median Values for Absolute Shift and Similarity for All Functionals in TD-DFT Calculations

functional	average absolute shift (eV)	median absolute shift (eV)	average similarity (%)	median similarity (%)
O3LYP	0.42	0.29	86.4	90.3
B3LYP/G	0.44	0.37	86.7	92.5
B97	0.44	0.39	86.8	92.6
TPSSh	0.46	0.35	74.6	78.8
MN15	0.50	0.37	87.6	92.4
PBE0	0.51	0.37	87.0	92.2
revM06-L	0.52	0.39	87.1	93.2
r^2^SCAN	0.53	0.38	84.9	90.0
ωPBE	0.54	0.46	85.8	93.1
TPSS	0.59	0.44	83.4	90.2
CAM-B3LYP	0.69	0.61	87.0	92.2
ωB97X	0.84	0.71	85.5	91.9
revM11	1.04	0.78	86.9	92.2

For charge transfer transitions, range-separated functionals are an option to overcome the limitation of traditional DFT, splitting the electron–electron interaction into short- and long-range components and using different values of exact HF exchange for each distance. This, in theory, makes the description of a CT transition more accurate by correcting the interaction between separated charges and reducing the delocalization error.^47,61^ However, this was not the general trend observed in the results. Looking at Table 3, all four range-separated functionals tested (revM11, ωPBE, CAM-B3LYP, and ωB97X) were among the worst performers of the set, with revM11 having an average absolut shift over 1 eV. This error in the transition energy is not compensated for by the similarity values obtained by the range-separated functionals. Most of the functionals exhibited an average similarity of between 85.0 and 87.0%, which was also the case for the range-separated functionals, and the maximum value was obtained by MN15 87.6%. ωPBE showed a median value of 93.1%, which is very close to the maximum value of 93.2% obtained by revM06-L. The revM11 functional, however, showed the highest average shift value of 1.04 eV, making it the least effective functional among all the studied ones for predicting transition energies. The poor performance of ωB97X and revM11 for predicting transition energies for this set of compounds can be visualized in the box plot (Figure 8), where these functionals’ boxes lie way higher when compared to the other functionals. The six best performances, in terms of absolute shift values, were achieved by the O3LYP, B3LYP/G, B97, TPSSh, MN15, and PBE0 functionals, in ascending order of absolute shift. revM06-L stands as the middle value of 0.52 eV. The six worst performers were r^2^SCAN, ωPBE, TPSS, CAM-B3LYP, ωB97X, and revM11, also in ascending order of absolute shift.

Among the meta-GGA functionals, revM06-L is a great highlight with the second highest value for average (87.1%) and the highest value for median (93.2%) similarity, with an absolute shift value of 0.52 eV. Contrary to what was observed for molecular structure, the TPSSh hybrid functional does not appear to be a suitable method for predicting UV–vis spectra. Although it performs well in terms of absolute shift (with an average value of 0.46 eV), it shows the worst overall performance in terms of similarity, with a value of just 74.6%. Bearing in mind that the second worst similarity value, obtained for TPSS, was 83.4%, this result for TPSSh makes it significantly worse than any other method studied in this work in terms the shape obtained for the calculated UV–vis spectrum. This result can also be visualized in the box plot of similarities, where the TPSSh box is way below any other box on the plot. However, the average absolute shift value obtained was 0.46 eV, very close to the lowest value obtained (0.42 eV), indicating that the transition energies predicted for this functional are among the most accurate, even though the relative intensities are not as good. The O3LYP functional stands out as the best in predicting transition energies, with an average absolute shift value of 0.42 eV. However, the overall performance of the B97 and B3LYP/G functionals is also noteworthy, as they showed an average absolute shift value of 0.44 eV, but with significantly higher median similarities (92.6 and 92.5% for B97 and B3LYP, respectively, compared to 90.3% for O3LYP). When it comes to hybrid functionals, comparing the amount of HF exchange in each functional is essential for determining the optimal value for this. B3LYP and B97 have 20 and 21% HF exchange, respectively, in their formulations. This makes O3LYP, with 12% HF exchange, the hybrid functional that, for this set of compounds, yields more accurate energy transition values, even if it has slightly worse performance in shape prediction of UV–vis spectra, as evident from the median value. PBE0 and MN15 have 25 and 44% of HF exchange, respectively, and yield average and median similarity values that are not significantly different from those of B97 and B3LYP. When it comes to absolute shift values, the PBE0 and MN15 showed values that are higher than those for other hybrid methods, indicating that, in this case, increasing the amount of HF exchange in a hybrid functional beyond a value of approximately 20% may negatively impact the results.

The expected accuracy of the computed electronic spectrum with a given density function is of great importance for evaluating our results and for using them for practical advice. According to Zobel and González,^57^ a computed electronic spectrum of a medium- to large-sized molecule can be regarded as reasonably accurate if the differences between calculated and measured absorption bands are within the 0.1–0.5 eV range. Here, we can use the absolute shifts as a proxy for the error associated with a given functional for the energies of the absorption bands in the electronic spectrum. Thus, the five best functionals–O3LYP, B3LYP/G, B97, TPSSh, and MN15–obtained an average absolute shift below 0.5 eV, can be regarded as capable of describing the electronic spectra of the 17 iron-complexes in this study with reasonable accuracy.

Regarding the compounds studied, it is evident that the shape of the experimental UV–vis spectrum directly influences the ability of TD-DFT calculations to simulate this same spectrum with high similarity. Compounds 1, 2, and 3, for example, have simple experimental spectra with few distinct bands, resulting in an average similarity of over 97% (Figure 9).

**Figure 9:**
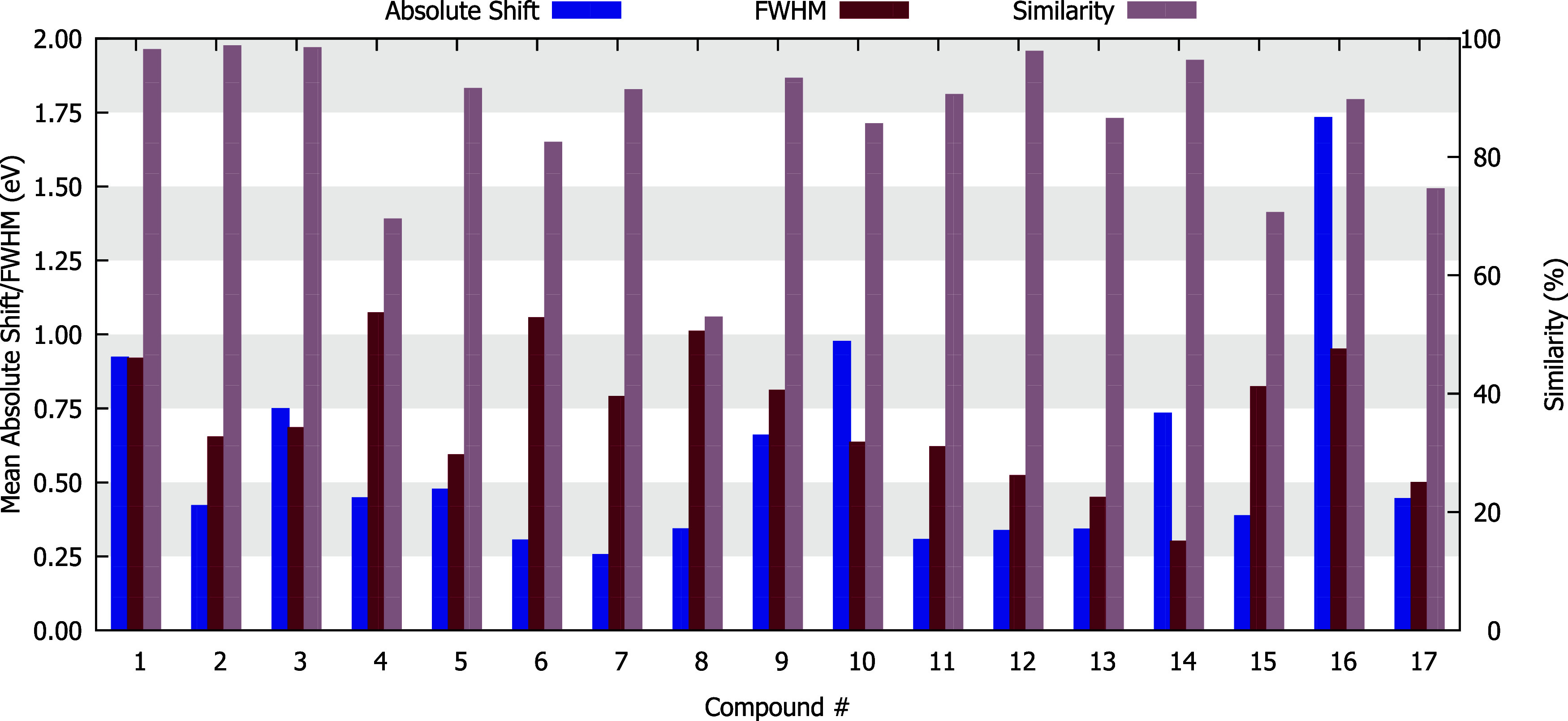
Average absolute shift, full width at half-maximum (FWHM), and similarity values obtained for every compound studied using TD-DFT in this work.

Complexes 4, 15, and 17 have experimental UV–vis spectra that contain several bands with different shapes, making them more challenging to simulate with high accuracy. Hence, the values of similarity for these compounds are rather low compared to others. However, compound 8 is an outlier in this analysis. It has an average similarity of only 52.9%, making it the lowest by a large margin. The low average similarity value reflects an inversion in the intensities (oscillator strength) observed in the vast majority of the functionals used. The experimental spectrum of compound 8 exhibits two distinct bands. The higher energy band corresponds to a π → π* transition of the phenanthroline ligand, while the lower energy band corresponds to an MLCT transition.^80^ Specifically for this compound, the range-separated methods showed more satisfactory results compared to other categories. The shape of the spectrum calculated by the CAM-B3LYP, revM11, and ωB97X functionals remained close to the experimental result, while all the other functionals showed inversions in the intensities of the two transitions, causing the MLCT to show a much higher intensity and distorting the shape of the spectrum compared to the experimental one.

Figure 10 shows a comparison between the TD-DFT spectrum calculated using the O3LYP functional, which exhibits one of the best overall performances, and that calculated using the CAM-B3LYP functional for compound 8.

**Figure 10:**
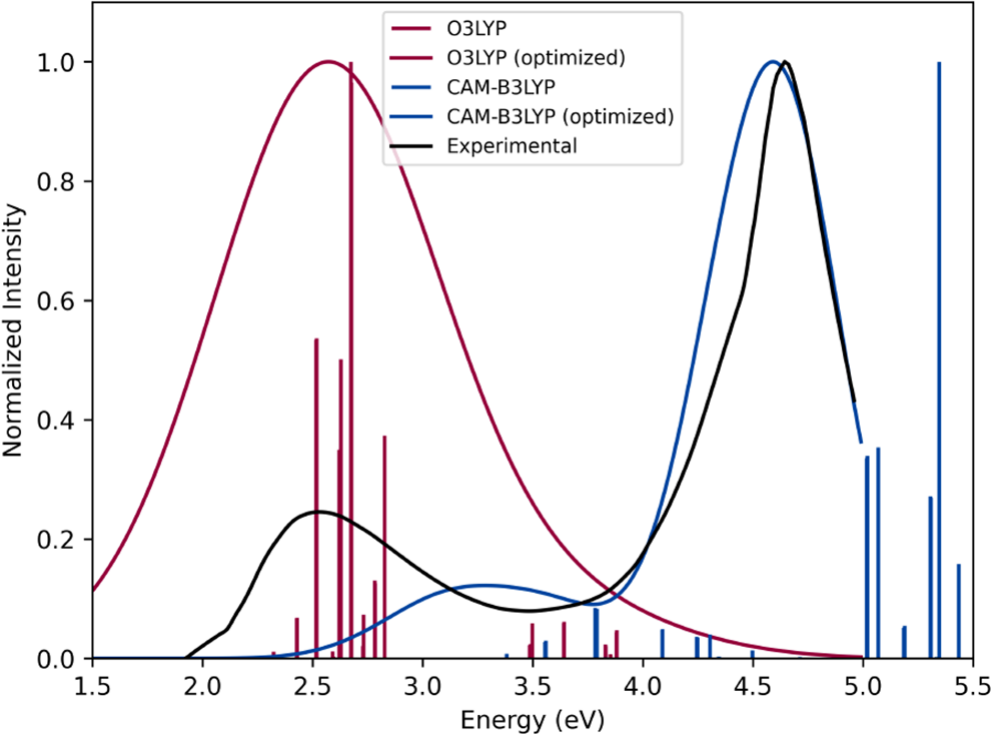
O3LYP/def2-TZVP calculated (red spikes) and optimized (red curve) TD-DFT spectrum and CAM-B3LYP/def2-TZVP calculated (blue spikes) and optimized (blue curve) of compound 8 ([Fe­(phen)_3_]^2+^) in comparison with experimental UV–vis data (black curve).

This same pattern was observed for a series of iron-phenantrolines whose TD-DFT spectrum was calculated using GGA and hybrid methods.^143^ This result shows that, in specific cases, range-separated methods can be the best choice for describing the electronic structure of a compound, particularly in the context of UV–vis spectroscopy. Phenanthroline is a ligand that shows greater electronic delocalization due to the presence of more resonance forms, especially when compared to common coordination ligands such as simple amines or pyridines. The presence of three of these ligands in compound 8 makes this system highly delocalized, which may be why range-separated methods are the most effective for predicting the shape of its electronic spectrum, given the long-range interactions present throughout the molecule. This result, in combination with what has already been presented in this paper, underscores the importance of benchmarking the most diverse types of systems. Depending on the type of property being studied, the use of different functionalities may be more appropriate for a given system based on its characteristics. For TD-DFT calculations of mononuclear iron complexes, the results obtained in this work were that the hybrid functionals B97 and B3LYP are the best when it comes to the complete description of the electronic spectrum, both in terms of energies and the shape of the spectrum. However, a good alternative is the O3LYP functional, which, despite having slightly lower performance in terms of spectral shape, shows greater accuracy in calculated energy values. Regarding the shape of the spectrum, the meta-GGA revM06-L functional was the most outstanding among the functionals studied, offering a relatively low computational cost and the highest median similarity and the second highest average similarity. Despite not having a good overall performance, the high cost of the range-separated functionals may be what is needed to calculate TD-DFT spectra of highly conjugated structures, as is the case with compound 8, indicating that the correction for short and long-range interactions introduced in this class of functionals is essential for describing this specific type of structure.

## 5 Conclusions

In this work, we discuss the performance of widely available standard approaches that are implemented in various electronic structure codes. Certainly, the performance of some methods could be fine-tuned (convergence parameters, TD-DFT without TDA, relativistic effects, etc.) but this would increase the computational cost of the calculations and would not correspond to the calculations routinely performed. For geometry optimizations, taking into account only the bonds involving the metal center, the best functional is TPSSh­(D4). However, unconventional geometries (those that differ from the more common octahedral geometry for Fe­(II), for example) associated with the characteristics of the ligands can still be particularly difficult to determine with acceptable accuracy, as observed for compound 13. For the prediction of UV–vis absorption spectra via TD-DFT, the hybrid functional O3LYP emerged as the best descriptor for vertical excitation energies, yielding the lowest average absolute shift (0.42 eV). The meta-GGA functional revM06-L is a highlight at reproducing the overall shape of the experimental spectra with remarkable fidelity and computational efficiency (87.1% of average similarity and 93.2% of median similarity). This divergence highlights a critical point: the “best” functional is dependent on the specific property of interest and must be chosen taking this property into account.

## Supplementary Material



## Notes

All optimized Cartesian coordinates; compounds 1–17 names are listed in Table S1; deviations calculated for geometry optimizations for all compounds and methods are described in Tables S2–S36; calculated, optimized and electronic absorption spectra for all compounds and TD-DFT functionals are shown in Figures S1–S30; absolute shift values, full width at half-maximum (FWHM) and similarity values are available in Tables S37–S68 (PDF)
