# Supplementary material for: Benchmarking Structures and UV–Vis Spectra of Iron Complexes Against Experimental Data
Source: J Phys Chem A. 2025 Oct 29;129(45):10481–95. doi: 10.1021/acs.jpca.5c06391 (PMC12621257; doi:10.1021/acs.jpca.5c06391)
Supplement: Supplementary file 1 [file jp5c06391_si_001.pdf]

Supporting Information: Benchmarking Structures and UV-Vis Spectra of Iron Complexes Against Experimental Data †

Renan R. Bertoloni,‡ Vania M. Ramos,¶ Ana Paula de Lima Batista,*,¶ and Antonio G. S. de Oliveira-Filho*,‡,§

‡*Departamento de Química, Faculdade de Filosofia, Ciências e Letras de Ribeirão Preto, Universidade de São Paulo, 14040-901, Ribeirão Preto, SP, Brazil*

¶*Departamento de Química, Grupo Computacional de Catálise e Espectroscopia (GCCE), Universidade Federal de São Carlos (UFSCar), 13565-905, São Carlos, SP, Brazil*

§*Instituto de Química de São Carlos, Universidade de São Paulo, 13560-970, São Carlos, SP, Brazil*

E-mail: aplbatista@ufscar.br; antoniogsof@iqsc.usp.br

†Prepared for J. Phys. Chem. October 20, 2025.

Contents

List of Figures **S-2**

List of Tables **S-6**

1 Molecular Structure Calculations **S-16**

1.1 Template for calculations S-16

1.2 Organized by molecule S-17

1.3 Organized by method S-35

2 TD-DFT Calculations **S-52**

2.1 Template for calculations S-52

2.2 Organized by molecule S-53

2.3 Organized by method S-88

References **S-115**

List of Figures

S1 Calculated and optimized TD-DFT spectra of compound 1 in comparison with experimental UV-Vis data. Calculations were made simulating a gaseous medium. S-54

S2 Calculated and optimized TD-DFT spectra of compound 2 in comparison with experimental UV-Vis data. Calculations were made in isopentane using the CPCM model. S-56

S3 Calculated and optimized TD-DFT spectra of compound 3 in comparison with experimental UV-Vis data. Calculations were made in DMSO using the CPCM model. S-58

S4 Calculated and optimized TD-DFT spectra of compound 4 in comparison with experimental UV-Vis data. Calculations were made in DMSO using the ACN model. S-60
S5 Calculated and optimized TD-DFT spectra of compound 5 in comparison with experimental UV-Vis data. Calculations were made in ACN using the CPCM model. S-62
S6 Calculated and optimized TD-DFT spectra of compound 6 in comparison with experimental UV-Vis data. Calculations were made in ACN using the CPCM model. S-64
S7 Calculated and optimized TD-DFT spectra of compound 7 in comparison with experimental UV-Vis data. Calculations were made in ACN using the CPCM model. S-66
S8 Calculated and optimized TD-DFT spectra of compound 8 in comparison with experimental UV-Vis data. Calculations were made in H_2O using the CPCM model. S-68
S9 Calculated and optimized TD-DFT spectra of compound 9 in comparison with experimental UV-Vis data. Calculations were made in ACN using the CPCM model. S-70
S10 Calculated and optimized TD-DFT spectra of compound 10 in comparison with experimental UV-Vis data. Calculations were made in ACN using the CPCM model. S-72
S11 Calculated and optimized TD-DFT spectra of compound 11 in comparison with experimental UV-Vis data. Calculations were made in ACN using the CPCM model. S-74
S12 Calculated and optimized TD-DFT spectra of compound 12 in comparison with experimental UV-Vis data. Calculations were made in DCM using the CPCM model. S-76

S13 Calculated and optimized TD-DFT spectra of compound 13 in comparison with experimental UV-Vis data. Calculations were made in H_2O using the CPCM model. S-78

S14 Calculated and optimized TD-DFT spectra of compound 14 in comparison with experimental UV-Vis data. Calculations were made in DCM using the CPCM model. S-80

S15 Calculated and optimized TD-DFT spectra of compound 15 in comparison with experimental UV-Vis data. Calculations were made in H_2O using the CPCM model. S-82

S16 Calculated and optimized TD-DFT spectra of compound 16 in comparison with experimental UV-Vis data. Calculations were made in CH_3OH using the CPCM model. S-84

S17 Calculated and optimized TD-DFT spectra of compound 17 in comparison with experimental UV-Vis data. Calculations were made in H_2O using the CPCM model. S-86

S18 Calculated and optimized TPSS/def2-TZVP TD-DFT spectra for all compounds in comparison with experimental UV-Vis data. Labels correspond to the respective compound. S-89

S19 Calculated and optimized r^{2}SCAN/def2-TZVP TD-DFT spectra for all compounds in comparison with experimental UV-Vis data. Labels correspond to the respective compound. S-91

S20 Calculated and optimized revM06L/def2-TZVP TD-DFT spectra for all compounds in comparison with experimental UV-Vis data. Labels correspond to the respective compound. S-93

S21 Calculated and optimized TPSSh/def2-TZVP TD-DFT spectra for all compounds in comparison with experimental UV-Vis data. Labels correspond to the respective compound. S-95

S22 Calculated and optimized O3LYP/def2-TZVP TD-DFT spectra for all compounds in comparison with experimental UV-Vis data. Labels correspond to the respective compound. S-97
S23 Calculated and optimized B97/def2-TZVP TD-DFT spectra for all compounds in comparison with experimental UV-Vis data. Labels correspond to the respective compound. S-99
S24 Calculated and optimized B3LYP(G)/def2-TZVP TD-DFT spectra for all compounds in comparison with experimental UV-Vis data. Labels correspond to the respective compound. S-101
S25 Calculated and optimized revM11/def2-TZVP TD-DFT spectra for all compounds in comparison with experimental UV-Vis data. Labels correspond to the respective compound. S-103
S26 Calculated and optimized PBE0/def2-TZVP TD-DFT spectra for all compounds in comparison with experimental UV-Vis data. Labels correspond to the respective compound. S-105
S27 Calculated and optimized MN15/def2-TZVP TD-DFT spectra for all compounds in comparison with experimental UV-Vis data. Labels correspond to the respective compound. S-107
S28 Calculated and optimized ωPBE/def2-TZVP TD-DFT spectra for all compounds in comparison with experimental UV-Vis data. Labels correspond to the respective compound. S-109
S29 Calculated and optimized CAM-B3LYP/def2-TZVP TD-DFT spectra for all compounds in comparison with experimental UV-Vis data. Labels correspond to the respective compound. S-111
S30 Calculated and optimized ωB97X/def2-TZVP TD-DFT spectra for all compounds in comparison with experimental UV-Vis data. Labels correspond to the respective compound. S-113

List of Tables

S1 Number, name, medium in which the UV-Vis spectrum was recorded, spin multiplicity and respective references for all the compounds studied in this work. S-15

S2 Calculated errors for compound 1 using various DFT functionals with the def2-TZVP basis set, as well as composite methods. Errors are reported in comparison to experimental X-ray diffraction data. Values are presented in ascending order of MUE. S-17

S3 Calculated errors for compound 2 using various DFT functionals with the def2-TZVP basis set, as well as composite methods. Errors are reported in comparison to experimental X-ray diffraction data. Values are presented in ascending order of MUE. S-18

S4 Calculated errors for compound 3 using various DFT functionals with the def2-TZVP basis set, as well as composite methods. Errors are reported in comparison to experimental X-ray diffraction data. Values are presented in ascending order of MUE. S-19

S5 Calculated errors for compound 4 using various DFT functionals with the def2-TZVP basis set, as well as composite methods. Errors are reported in comparison to experimental X-ray diffraction data. Values are presented in ascending order of MUE. S-20

S6 Calculated errors for compound 5 using various DFT functionals with the def2-TZVP basis set, as well as composite methods. Errors are reported in comparison to experimental X-ray diffraction data. Values are presented in ascending order of MUE. S-21

S7 Calculated errors for compound 6 using various DFT functionals with the def2-TZVP basis set, as well as composite methods. Errors are reported in comparison to experimental X-ray diffraction data. Values are presented in ascending order of MUE. S-22

S8 Calculated errors for compound 7 using various DFT functionals with the def2-TZVP basis set, as well as composite methods. Errors are reported in comparison to experimental X-ray diffraction data. Values are presented in ascending order of MUE. S-23

S9 Calculated errors for compound 8 using various DFT functionals with the def2-TZVP basis set, as well as composite methods. Errors are reported in comparison to experimental X-ray diffraction data. Values are presented in ascending order of MUE. S-24

S10 Calculated errors for compound 9 using various DFT functionals with the def2-TZVP basis set, as well as composite methods. Errors are reported in comparison to experimental X-ray diffraction data. Values are presented in ascending order of MUE. S-25

S11 Calculated errors for compound 10 using various DFT functionals with the def2-TZVP basis set, as well as composite methods. Errors are reported in comparison to experimental X-ray diffraction data. Values are presented in ascending order of MUE. S-26

S12 Calculated errors for compound 11 using various DFT functionals with the def2-TZVP basis set, as well as composite methods. Errors are reported in comparison to experimental X-ray diffraction data. Values are presented in ascending order of MUE. S-27

S13 Calculated errors for compound 12 using various DFT functionals with the def2-TZVP basis set, as well as composite methods. Errors are reported in comparison to experimental X-ray diffraction data. Values are presented in ascending order of MUE. S-28
S14 Calculated errors for compound 13 using various DFT functionals with the def2-TZVP basis set, as well as composite methods. Errors are reported in comparison to experimental X-ray diffraction data. Values are presented in ascending order of MUE. S-29
S15 Calculated errors for compound 14 using various DFT functionals with the def2-TZVP basis set, as well as composite methods. Errors are reported in comparison to experimental X-ray diffraction data. Values are presented in ascending order of MUE. S-30
S16 Calculated errors for compound 15 using various DFT functionals with the def2-TZVP basis set, as well as composite methods. Errors are reported in comparison to experimental X-ray diffraction data. Values are presented in ascending order of MUE. S-31
S17 Calculated errors for compound 16 using various DFT functionals with the def2-TZVP basis set, as well as composite methods. Errors are reported in comparison to experimental X-ray diffraction data. Values are presented in ascending order of MUE. S-32
S18 Calculated errors for compound 17 using various DFT functionals with the def2-TZVP basis set, as well as composite methods. Errors are reported in comparison to experimental X-ray diffraction data. Values are presented in ascending order of MUE. S-33

S19 Average errors per molecule calculated using density functional theory (DFT) functionals with the def2-TZVP basis set and composite methods. Errors are averaged across molecular geometries and benchmarked against experimental X-ray diffraction data. S-34

S20 Calculated errors obtained with GFN1-xTB method. Errors are reported in comparison to experimental X-ray diffraction data. Values are presented in ascending order of MUE. S-35

S21 Calculated errors obtained with HF-3c composite method. Errors are reported in comparison to experimental X-ray diffraction data. Values are presented in ascending order of MUE. S-36

S22 Calculated errors obtained with PBEh-3c composite method. Errors are reported in comparison to experimental X-ray diffraction data. Values are presented in ascending order of MUE. S-37

S23 Calculated errors obtained with r^{2}SCAN-3c composite method. Errors are reported in comparison to experimental X-ray diffraction data. Values are presented in ascending order of MUE. S-38

S24 Calculated errors obtained with BP86(D4) functional and def2-TZVP basis set. Errors are reported in comparison to experimental X-ray diffraction data. Values are presented in ascending order of MUE. S-39

S25 Calculated errors obtained with PBE(D4) functional and def2-TZVP basis set. Errors are reported in comparison to experimental X-ray diffraction data. Values are presented in ascending order of MUE. S-40

S26 Calculated errors obtained with revPBE(D4) functional and def2-TZVP basis set. Errors are reported in comparison to experimental X-ray diffraction data. Values are presented in ascending order of MUE. S-41

S27 Calculated errors obtained with OPBE(D4) functional and def2-TZVP basis set. Errors are reported in comparison to experimental X-ray diffraction data. Values are presented in ascending order of MUE. S-42
S28 Calculated errors obtained with B97(D4) functional and def2-TZVP basis set. Errors are reported in comparison to experimental X-ray diffraction data. Values are presented in ascending order of MUE. S-43
S29 Calculated errors obtained with B3LYP/G(D4) functional and def2-TZVP basis set. Errors are reported in comparison to experimental X-ray diffraction data. Values are presented in ascending order of MUE. S-44
S30 Calculated errors obtained with r^2SCAN functional and def2-TZVP basis set. Errors are reported in comparison to experimental X-ray diffraction data. Values are presented in ascending order of MUE. S-45
S31 Calculated errors obtained with TPSS(D4) functional and def2-TZVP basis set. Errors are reported in comparison to experimental X-ray diffraction data. Values are presented in ascending order of MUE. S-46
S32 Calculated errors obtained with MN15 functional and def2-TZVP basis set. Errors are reported in comparison to experimental X-ray diffraction data. Values are presented in ascending order of MUE. S-47
S33 Calculated errors obtained with revM11 functional and def2-TZVP basis set. Errors are reported in comparison to experimental X-ray diffraction data. Values are presented in ascending order of MUE. S-48
S34 Calculated errors obtained with TPSSh(D4) functional and def2-TZVP basis set. Errors are reported in comparison to experimental X-ray diffraction data. Values are presented in ascending order of MUE. S-49
S35 Calculated errors obtained with ωB97X(D4) functional and def2-TZVP basis set. Errors are reported in comparison to experimental X-ray diffraction data. Values are presented in ascending order of MUE. S-50

S36 Average errors per method calculated. Errors are averaged across molecular geometries and benchmarked against experimental X-ray diffraction data. . . S-51
S37 Calculated errors for the TD-DFT spectra of compound 1 in comparison with experimental UV-Vis data. Calculations were made simulating a gaseous medium. S-53
S38 Calculated errors for the TD-DFT spectra of compound 2 in comparison with experimental UV-Vis data. Calculations were made in isopentane using the CPCM model. S-55
S39 Calculated errors for the TD-DFT spectra of compound 3 in comparison with experimental UV-Vis data. Calculations were made in DMSO using the CPCM model. S-57
S40 Calculated errors for the TD-DFT spectra of compound 4 in comparison with experimental UV-Vis data. Calculations were made in ACN using the CPCM model. S-59
S41 Calculated errors for the TD-DFT spectra of compound 5 in comparison with experimental UV-Vis data. Calculations were made in ACN using the CPCM model. S-61
S42 Calculated errors for the TD-DFT spectra of compound 6 in comparison with experimental UV-Vis data. Calculations were made in ACN using the CPCM model. S-63
S43 Calculated errors for the TD-DFT spectra of compound 7 in comparison with experimental UV-Vis data. Calculations were made in ACN using the CPCM model. S-65
S44 Calculated errors for the TD-DFT spectra of compound 8 in comparison with experimental UV-Vis data. Calculations were made in H_2O using the CPCM model. S-67

S45 Calculated errors for TD-DFT spectra of compound 9 in comparison with experimental UV-Vis data. Calculations were made in ACN using the CPCM model. S-69
S46 Calculated errors for the TD-DFT spectra of compound 10 in comparison with experimental UV-Vis data. Calculations were made in ACN using the CPCM model. S-71
S47 Calculated errors for the TD-DFT spectra of compound 11 in comparison with experimental UV-Vis data. Calculations were made in ACN using the CPCM model. S-73
S48 Calculated errors for the TD-DFT spectra of compound 12 in comparison with experimental UV-Vis data. Calculations were made in DCM using the CPCM model. S-75
S49 Calculated errors for the TD-DFT spectra of compound 13 in comparison with experimental UV-Vis data. Calculations were made in H_2O using the CPCM model. S-77
S50 Calculated errors for the TD-DFT spectra of compound 14 in comparison with experimental UV-Vis data. Calculations were made in DCM using the CPCM model. S-79
S51 Calculated errors for the TD-DFT spectra of compound 15 in comparison with experimental UV-Vis data. Calculations were made in H_2O using the CPCM model. S-81
S52 Calculated errors for the TD-DFT spectra of compound 16 in comparison with experimental UV-Vis data. Calculations were made in CH_3OH using the CPCM model. S-83
S53 Calculated errors for the TD-DFT spectra of compound 17 in comparison with experimental UV-Vis data. Calculations were made in H_2O using the CPCM model. S-85

S54 Calculated average errors for TD-DFT spectra of all compounds in comparison with experimental UV-Vis data. S-87
S55 Calculated errors for the TD-DFT spectra of all compounds obtained with the TPSS/def2-TZVP method in comparison with experimental UV-Vis data. S-88
S56 Calculated errors for the TD-DFT spectra of all compounds obtained with the r^{2}SCAN/def2-TZVP method in comparison with experimental UV-Vis data. S-90
S57 Calculated errors for the TD-DFT spectra of all compounds obtained with the revM06L/def2-TZVP method in comparison with experimental UV-Vis data. S-92
S58 Calculated errors for the TD-DFT spectra of all compounds obtained with the TPSSh/def2-TZVP method in comparison with experimental UV-Vis data.S-94
S59 Calculated errors for the TD-DFT spectra of all compounds obtained with the O3LYP/def2-TZVP method in comparison with experimental UV-Vis data.S-96
S60 Calculated errors for the TD-DFT spectra of all compounds obtained with the B97/def2-TZVP method in comparison with experimental UV-Vis data. S-98
S61 Calculated errors for the TD-DFT spectra of all compounds obtained with the B3LYP(G)/def2-TZVP method in comparison with experimental UV-Vis data. S-100
S62 Calculated errors for the TD-DFT spectra of all compounds obtained with the revM11/def2-TZVP method in comparison with experimental UV-Vis data.S-102
S63 Calculated errors for the TD-DFT spectra of all compounds obtained with the PBE0/def2-TZVP method in comparison with experimental UV-Vis data. S-104
S64 Calculated errors for TD-DFT spectra of all compounds obtained with the MN15/def2-TZVP method in comparison with experimental UV-Vis data. . S-106
S65 Calculated errors for TD-DFT spectra of all compounds obtained with the ωPBE/def2-TZVP method in comparison with experimental UV-Vis data. . S-108

S66 Calculated errors for TD-DFT spectra of all compounds obtained with the CAM-B3LYP/def2-TZVP method in comparison with experimental UV-Vis data. S-110
S67 Calculated errors for the TD-DFT spectra of all compounds obtained with the ωB97X/def2-TZVP method in comparison with experimental UV-Vis data. S-112
S68 Average calculated errors for the TD-DFT spectra obtained with every method in comparison with experimental UV-Vis data. S-114

Table S1: Number, name, medium in which the UV-Vis spectrum was recorded, spin multiplicity and respective references for all the compounds studied in this work.

Compound	Name	Medium	Spin Multiplicity	Ref.
1	pentacarbonyliron(0)	Gaseous	1	S1
2	bis(cyclopentadienyl)iron(II)	Isopentane	1	S2–S4
3	carbonyl-cyclopentadienyl-triphenylphosphane-iron(II)	Dimethyl Sulfoxide	1	S5
4	2,2′-bipyridine-tetracyano-iron(II)	Acetonitrile	1	S6,S7
5	tris(2,2′-bipyridine)-iron(II)	Acetonitrile	1	S6,S8
6	bis(2,2′:6′,2′;-terpyridine)-iron(II)	Acetonitrile	1	S9,S10
7	bis(6,6′′-difluoro-2,2′:6′,2′′-terpyridine)-iron(II)	Acetonitrile	1	S11
8	tris(1,10-phenanthroline)-iron(II)	Water	1	S12,S13
9	bis[2,6-bis(pyrazol-1-yl)benzonitrile]-iron(II)	Acetonitrile	1	S14
10	dichloro-N,N′-dimethyl-N,N′-bis(4-nitro-pyridin-2-ylmethyl)-ethane-1,2-diamine-iron(II)	Acetonitrile	5	S15
11	diacetonitrile-[(1E,1′E)-1,1′-(phenylazanediyl)bis(3,1-phenylene)-bis(ethan-1-one)-O,O-dimethyldioxime]-iron(II)	Acetonitrile	1	S16
12	benzenethiolate-2-((bis(pyridin-2-ylmethyl)amino)methyl) -6-formyl-4-methylphenolate-iron(II)	Dichloromethane	1	S17
13	carbonyl-1,1-di(pyridin-2-yl)-N,N-bis(pyridin-2-ylmethyl)-methanamine-iron(II)	Water	1	S18
14	dicyano-(5,5,7,12,12,14-hexamethyl-1,4,8,11 -tetraazacyclotetradeca-1,3,8,10-tetraene)-iron(II)	Dichloromethane	1	S19
15	N-(2-((carboxylatomethyl)-(2-oxidobenzyl)amino)ethyl)-N-(pyridin-2-ylmethyl)glycinate-iron(III)	Water	6	S20
16	4,6,10-trihydroxy-1,4,6,10-tetraazaadamantane-iron(IV)	Methanol	3	S21
17	(1R,3R)-5,6,14,15,20,21-hexaoxo-1,3,4,7,8,10,12,13,16,17,19,22-dodecaazatetracyclo[8.8.4.$1^{3,17}$.$1^{8,12}$] tetracosane-4,7,13,16,19,22-hexaide-iron(IV)	Water	3	S22

1 Molecular Structure Calculations

1.1 Template for calculations

All geometry optimizations were carried out using the following template for ORCA input files:

```
! "Functional/Method" D4 def2-TZVP Opt NumFreq


%maxcore 8000

%pal nprocs 8 end


*xyzfile "experimental_data.xyz" C S


# For HF-3c, PBEh-3c and r2SCAN-3c, no basis set was included

# C stands for the charge of the complex

# S stands for the spin multiplicity of the complex
```

1.2 Organized by molecule

Table S2: Calculated errors for compound 1 using various DFT functionals with the def2-TZVP basis set, as well as composite methods. Errors are reported in comparison to experimental X-ray diffraction data. Values are presented in ascending order of MUE.

**Method**	RMSE	MUE	MSE
MN15	0.0805	0.0181	-0.0027
B3LYP/G(D4)	0.0827	0.0192	-0.0001
TPSS(D4)	0.0808	0.0192	-0.0084
revPBE(D4)	0.0792	0.0196	-0.0114
BP86(D4)	0.0811	0.0207	-0.0130
TPSSh(D4)	0.0810	0.0209	-0.0113
B97(D4)	0.0790	0.0212	-0.0147
PBE(D4)	0.0812	0.0221	-0.0154
ωB97X(D4)	0.0813	0.0222	-0.0082
revM11	0.0802	0.0232	-0.0135
r^2SCAN-3c	0.0799	0.0246	-0.0182
GFN1-xTB	0.0811	0.0265	-0.0265
r^2SCAN	0.0800	0.0268	-0.0219
PBEh-3c	0.0878	0.0312	-0.0240
HF-3c	1.7443	0.0444	0.0291
OPBE(D4)	0.0878	0.0492	-0.0492

Table S3: Calculated errors for compound 2 using various DFT functionals with the def2-TZVP basis set, as well as composite methods. Errors are reported in comparison to experimental X-ray diffraction data. Values are presented in ascending order of MUE.

**Method**	RMSE	MUE	MSE
revPBE(D4)	0.2062	0.0208	0.0006
ωB97X(D4)	0.3631	0.0214	0.0036
r^2SCAN-3c	0.3659	0.0220	0.0004
PBEh-3c	0.2695	0.0226	0.0123
BP86(D4)	0.3672	0.0227	-0.0031
PBE(D4)	0.3672	0.0228	-0.0037
B97(D4)	0.3665	0.0230	-0.0044
r^2SCAN	0.3662	0.0232	-0.0063
MN15	0.3636	0.0233	-0.0031
TPSS(D4)	0.3670	0.0237	-0.0076
TPSSh(D4)	0.3659	0.0237	-0.0079
revM11	0.2319	0.0240	-0.0078
B3LYP/G(D4)	0.3644	0.0250	0.0246
OPBE(D4)	0.3746	0.0550	-0.0550
HF-3c	0.3837	0.1123	0.1123
GFN1-xTB	0.3844	0.1144	0.1144

Table S4: Calculated errors for compound 3 using various DFT functionals with the def2-TZVP basis set, as well as composite methods. Errors are reported in comparison to experimental X-ray diffraction data. Values are presented in ascending order of MUE.

**Method**	RMSE	MUE	MSE
revM11	0.4555	0.0206	0.0001
MN15	0.4521	0.0206	0.0032
TPSSh(D4)	0.5061	0.0304	0.0025
ωB97X(D4)	0.5235	0.0315	0.0063
TPSS(D4)	0.5044	0.0329	0.0033
PBE(D4)	0.5257	0.0392	0.0099
r^2SCAN	0.4987	0.0398	0.0147
revPBE(D4)	0.5126	0.0406	0.0090
PBEh-3c	0.5918	0.0406	0.0333
BP86(D4)	0.5493	0.0425	0.0140
r^2SCAN-3c	0.5083	0.0432	0.0219
B97(D4)	0.5523	0.0499	0.0072
B3LYP/G(D4)	0.5516	0.0523	0.0408
OPBE(D4)	0.5030	0.0545	-0.0544
GFN1-xTB	0.5946	0.1175	0.0619
HF-3c	0.5895	0.1603	0.1603

Table S5: Calculated errors for compound 4 using various DFT functionals with the def2-TZVP basis set, as well as composite methods. Errors are reported in comparison to experimental X-ray diffraction data. Values are presented in ascending order of MUE.

**Method**	RMSE	MUE	MSE
PBE(D4)	0.1452	0.0125	0.0107
BP86(D4)	0.1463	0.0143	0.0139
revPBE(D4)	0.1462	0.0163	0.0163
B97(D4)	0.1434	0.0205	0.0168
TPSS(D4)	0.1503	0.0234	0.0197
r^2SCAN	0.1483	0.0252	-0.0111
r^2SCAN-3c	0.1414	0.0286	-0.0040
TPSSh(D4)	0.1572	0.0290	0.0239
MN15	0.1755	0.0357	0.0098
GFN1-xTB	0.1821	0.0360	-0.0360
revM11	0.1895	0.0373	0.0373
B3LYP/G(D4)	0.1674	0.0401	0.0366
PBEh-3c	0.1775	0.0422	0.0333
ωB97X(D4)	0.1968	0.0437	0.0437
OPBE(D4)	0.5236	0.0589	-0.0236
HF-3c	0.2494	0.1279	0.1279

Table S6: Calculated errors for compound 5 using various DFT functionals with the def2-TZVP basis set, as well as composite methods. Errors are reported in comparison to experimental X-ray diffraction data. Values are presented in ascending order of MUE.

**Method**	RMSE	MUE	MSE
r^2SCAN	0.2021	0.0024	-0.0024
TPSSh(D4)	0.2300	0.0042	-0.0042
r^2SCAN-3c	0.2196	0.0054	0.0054
PBE(D4)	0.2171	0.0113	-0.0113
TPSS(D4)	0.2291	0.0137	-0.0137
MN15	0.2118	0.0146	0.0146
BP86(D4)	0.2289	0.0160	-0.0160
revPBE(D4)	0.2302	0.0179	-0.0179
B97(D4)	0.2322	0.0236	-0.0236
B3LYP/G(D4)	0.2234	0.0270	0.0270
ωB97X(D4)	0.2303	0.0326	0.0326
PBEh-3c	0.2848	0.0429	0.0429
revM11	0.2088	0.0442	0.0442
OPBE(D4)	0.2673	0.0456	-0.0456
HF-3c	0.2195	0.0481	0.0481
GFN1-xTB	0.2893	0.0528	-0.0528

Table S7: Calculated errors for compound 6 using various DFT functionals with the def2-TZVP basis set, as well as composite methods. Errors are reported in comparison to experimental X-ray diffraction data. Values are presented in ascending order of MUE.

**Method**	RMSE	MUE	MSE
MN15	0.0955	0.0069	0.0031
r^2SCAN-3c	0.0965	0.0089	-0.0075
B3LYP/G(D4)	0.1051	0.0131	0.0131
r^2SCAN	0.0979	0.0141	-0.0141
TPSSh(D4)	0.1014	0.0149	-0.0149
ωB97X(D4)	0.1150	0.0206	0.0206
PBE(D4)	0.1016	0.0214	-0.0214
TPSS(D4)	0.1015	0.0220	-0.0220
B97(D4)	0.1035	0.0241	-0.0241
BP86(D4)	0.1045	0.0244	-0.0244
revPBE(D4)	0.1058	0.0251	-0.0251
PBEh-3c	0.1177	0.0278	0.0278
revM11	0.0999	0.0310	0.0310
OPBE(D4)	0.1030	0.0350	-0.0350
HF-3c	0.1063	0.0463	0.0463
GFN1-xTB	0.1170	0.0486	-0.0486

Table S8: Calculated errors for compound 7 using various DFT functionals with the def2-TZVP basis set, as well as composite methods. Errors are reported in comparison to experimental X-ray diffraction data. Values are presented in ascending order of MUE.

**Method**	RMSE	MUE	MSE
r^2SCAN-3c	0.1171	0.0054	-0.0017
r^2SCAN	0.1155	0.0090	-0.0088
MN15	0.1180	0.0099	0.0098
TPSSh(D4)	0.1152	0.0100	-0.0100
PBE(D4)	0.1187	0.0170	-0.0170
TPSS(D4)	0.1194	0.0195	-0.0195
B3LYP/G(D4)	0.1190	0.0203	0.0203
BP86(D4)	0.1192	0.0209	-0.0209
revPBE(D4)	0.1206	0.0216	-0.0216
GFN1-xTB	0.1273	0.0250	-0.0250
B97(D4)	0.1211	0.0259	-0.0259
ωB97X(D4)	0.1151	0.0270	0.0270
PBEh-3c	0.1255	0.0315	0.0315
revM11	0.1211	0.0370	0.0370
HF-3c	0.1252	0.0453	0.0453
OPBE(D4)	0.1311	0.0579	-0.0579

Table S9: Calculated errors for compound 8 using various DFT functionals with the def2-TZVP basis set, as well as composite methods. Errors are reported in comparison to experimental X-ray diffraction data. Values are presented in ascending order of MUE.

**Method**	RMSE	MUE	MSE
MN15	0.1360	0.0062	-0.0001
r^2SCAN-3c	0.1270	0.0101	-0.0101
B3LYP/G(D4)	0.1164	0.0108	0.0108
ωB97X(D4)	0.1166	0.0165	0.0165
r^2SCAN	0.1203	0.0166	-0.0166
TPSSh(D4)	0.1337	0.0203	-0.0203
PBEh-3c	0.1367	0.0252	0.0252
PBE(D4)	0.1339	0.0272	-0.0272
revM11	0.1265	0.0287	0.0287
TPSS(D4)	0.1424	0.0290	-0.0290
BP86(D4)	0.1419	0.0323	-0.0323
revPBE(D4)	0.1439	0.0336	-0.0336
HF-3c	0.1349	0.0337	0.0337
B97(D4)	0.1439	0.0377	-0.0377
GFN1-xTB	0.1632	0.0542	-0.0542
OPBE(D4)	0.1729	0.0552	-0.0552

Table S10: Calculated errors for compound 9 using various DFT functionals with the def2-TZVP basis set, as well as composite methods. Errors are reported in comparison to experimental X-ray diffraction data. Values are presented in ascending order of MUE.

**Method**	RMSE	MUE	MSE
r^2SCAN	0.1723	0.0053	-0.0016
TPSSh(D4)	0.1768	0.0055	-0.0030
r^2SCAN-3c	0.1720	0.0078	0.0078
TPSS(D4)	0.1793	0.0126	-0.0126
PBE(D4)	0.1768	0.0126	-0.0126
GFN1-xTB	0.1711	0.0146	-0.0100
MN15	0.1850	0.0158	0.0158
BP86(D4)	0.1766	0.0158	-0.0158
revPBE(D4)	0.1786	0.0163	-0.0163
B97(D4)	0.1721	0.0219	-0.0219
B3LYP/G(D4)	0.1734	0.0271	0.0271
ωB97X(D4)	0.1815	0.0351	0.0351
PBEh-3c	0.1873	0.0383	0.0383
OPBE(D4)	0.1829	0.0395	-0.0395
revM11	0.1831	0.0441	0.0441
HF-3c	0.1828	0.0622	0.0622

Table S11: Calculated errors for compound 10 using various DFT functionals with the def2-TZVP basis set, as well as composite methods. Errors are reported in comparison to experimental X-ray diffraction data. Values are presented in ascending order of MUE.

**Method**	RMSE	MUE	MSE
HF-3c	0.1513	0.0396	-0.0175
B3LYP/G(D4)	0.1108	0.0611	0.0335
TPSSh(D4)	0.1476	0.0659	-0.0106
PBEh-3c	0.1036	0.0686	0.0409
r^2SCAN-3c	0.1493	0.0693	-0.0061
TPSS(D4)	0.1625	0.0771	-0.0233
OPBE(D4)	0.2324	0.0884	-0.0655
BP86(D4)	0.1613	0.0912	-0.0139
B97(D4)	0.1793	0.1120	-0.1120
revPBE(D4)	0.1657	0.1162	-0.1160
PBE(D4)	0.1540	0.1168	-0.1133
r^2SCAN	0.1784	0.1178	-0.1178
revM11	0.1823	0.1423	-0.1423
MN15	0.1802	0.1453	-0.1453
ωB97X(D4)	0.2428	0.1522	-0.1522
GFN1-xTB	0.1686	0.2630	0.0724

Table S12: Calculated errors for compound 11 using various DFT functionals with the def2-TZVP basis set, as well as composite methods. Errors are reported in comparison to experimental X-ray diffraction data. Values are presented in ascending order of MUE.

**Method**	RMSE	MUE	MSE
MN15	0.2300	0.0112	0.0097
B3LYP/G(D4)	0.2308	0.0162	0.0162
r^2SCAN	0.2153	0.0163	-0.0103
r^2SCAN-3c	0.2153	0.0167	0.0019
PBEh-3c	0.2264	0.0170	0.0170
TPSSh(D4)	0.2132	0.0182	-0.0036
TPSS(D4)	0.2093	0.0227	-0.0090
ωB97X(D4)	0.2434	0.0240	0.0240
PBE(D4)	0.2073	0.0244	-0.0111
BP86(D4)	0.2023	0.0247	-0.0113
revPBE(D4)	0.2107	0.0254	-0.0099
B97(D4)	0.2128	0.0265	-0.0097
revM11	0.2445	0.0270	0.0270
OPBE(D4)	0.2040	0.0347	-0.0327
GFN1-xTB	0.2889	0.0449	-0.0396
HF-3c	0.2332	0.0574	0.0574

Table S13: Calculated errors for compound 12 using various DFT functionals with the def2-TZVP basis set, as well as composite methods. Errors are reported in comparison to experimental X-ray diffraction data. Values are presented in ascending order of MUE.

**Method**	RMSE	MUE	MSE
HF-3c	1.0476	0.0934	-0.1085
PBEh-3c	0.5423	0.1121	-0.1270
B3LYP/G(D4)	1.0860	0.1317	-0.1282
ωB97X(D4)	1.0962	0.12862	-0.1278
MN15	1.1096	0.1408	-0.1469
r^2SCAN	0.9862	0.1781	-0.1603
TPSSh(D4)	1.1233	0.1648	-0.1583
PBE(D4)	1.0940	0.1671	-0.1646
revM11	1.9294	0.1430	-0.1754
GFN1-xTB	1.1303	0.1744	-0.1628
r^2SCAN-3c	1.2848	0.1781	-0.2063
TPSS(D4)	2.0983	0.1791	-0.2117
revPBE(D4)	2.0416	0.1901	-0.2141
BP86(D4)	2.0050	0.1928	-0.2163
B97(D4)	2.0320	0.1982	-0.2227
OPBE(D4)	2.0922	0.2370	-0.2622

Table S14: Calculated errors for compound 13 using various DFT functionals with the def2-TZVP basis set, as well as composite methods. Errors are reported in comparison to experimental X-ray diffraction data. Values are presented in ascending order of MUE.

**Method**	RMSE	MUE	MSE
GFN1-xTB	0.0436	0.0133	-0.0132
TPSS(D4)	0.0522	0.0139	0.0092
r^2SCAN	0.0381	0.0143	0.0079
TPSSh(D4)	0.0468	0.0153	0.0153
MN15	0.0656	0.0164	0.0164
revPBE(D4)	0.0517	0.0173	0.0079
BP86(D4)	0.0480	0.0174	0.0081
PBE(D4)	0.0420	0.0182	0.0084
r^2SCAN-3c	0.0483	0.0189	0.0154
B97(D4)	0.0488	0.0208	0.0084
OPBE(D4)	0.0761	0.0344	-0.0091
B3LYP/G(D4)	0.0443	0.0348	0.0348
ωB97X(D4)	0.0482	0.0348	0.0348
revM11	0.0519	0.0369	0.0369
PBEh-3c	0.0509	0.0418	0.0418
HF-3c	0.0844	0.0814	0.0814

Table S15: Calculated errors for compound 14 using various DFT functionals with the def2-TZVP basis set, as well as composite methods. Errors are reported in comparison to experimental X-ray diffraction data. Values are presented in ascending order of MUE.

**Method**	RMSE	MUE	MSE
MN15	0.1200	0.0049	-0.0008
r^2SCAN-3c	0.1495	0.0061	-0.0026
TPSSh(D4)	0.1771	0.0078	-0.0069
r^2SCAN	0.1447	0.0085	-0.0085
TPSS(D4)	0.1671	0.0092	-0.0089
PBEh-3c	0.1838	0.0145	0.0145
revM11	0.1391	0.0178	0.0178
BP86(D4)	0.1716	0.0204	-0.0104
PBE(D4)	0.1765	0.0233	-0.0104
B97(D4)	0.1717	0.0234	-0.0145
revPBE(D4)	0.1816	0.0238	-0.0115
B3LYP/G(D4)	0.1985	0.0350	0.0350
ωB97X(D4)	0.1645	0.0376	0.0376
GFN1-xTB	0.5390	0.0599	-0.0583
HF-3c	0.2578	0.0616	0.0616
OPBE(D4)	0.2254	0.0625	-0.0625

Table S16: Calculated errors for compound 15 using various DFT functionals with the def2-TZVP basis set, as well as composite methods. Errors are reported in comparison to experimental X-ray diffraction data. Values are presented in ascending order of MUE.

**Method**	RMSE	MUE	MSE
OPBE(D4)	0.2837	0.0255	0.0021
ωB97X(D4)	0.3172	0.0310	0.0050
TPSSh(D4)	0.2844	0.0328	0.0161
B97(D4)	0.2934	0.0357	0.0272
TPSS(D4)	0.2824	0.0366	0.0263
revM11	0.2902	0.0372	0.0170
r^2SCAN-3c	0.2740	0.0381	0.0306
r^2SCAN	0.2838	0.0390	0.0245
B3LYP/G(D4)	0.2983	0.0420	0.0262
MN15	0.3130	0.0427	0.0235
revPBE(D4)	0.2894	0.0437	0.0402
BP86(D4)	0.2803	0.0448	0.0372
PBE(D4)	0.2832	0.0458	0.0402
PBEh-3c	0.2865	0.0513	0.0099
HF-3c	0.3100	0.0651	-0.0563
GFN1-xTB	0.4844	0.2625	0.2625

Table S17: Calculated errors for compound 16 using various DFT functionals with the def2-TZVP basis set, as well as composite methods. Errors are reported in comparison to experimental X-ray diffraction data. Values are presented in ascending order of MUE.

**Method**	RMSE	MUE	MSE
PBE(D4)	0.0959	0.0625	-0.0123
B3LYP/G(D4)	0.0941	0.0636	-0.0086
r^2SCAN-3c	0.2390	0.0643	-0.0144
BP86(D4)	0.0938	0.0645	-0.0157
revPBE(D4)	0.2348	0.0654	-0.0151
B97(D4)	0.2274	0.0683	-0.0187
TPSS(D4)	0.2470	0.0701	-0.0276
r^2SCAN	0.2398	0.0713	-0.0262
ωB97X(D4)	0.1832	0.0731	-0.0193
TPSSh(D4)	0.2619	0.0747	-0.0320
revM11	0.3165	0.0796	-0.0082
MN15	0.3123	0.0845	-0.0389
GFN1-xTB	0.2459	0.0867	0.0274
PBEh-3c	0.1687	0.0911	0.0425
OPBE(D4)	0.2576	0.0971	-0.0615
HF-3c	0.3886	0.1236	0.0434

Table S18: Calculated errors for compound 17 using various DFT functionals with the def2-TZVP basis set, as well as composite methods. Errors are reported in comparison to experimental X-ray diffraction data. Values are presented in ascending order of MUE.

**Method**	RMSE	MUE	MSE
TPSSh(D4)	0.1221	0.0074	-0.0024
revM11	0.1083	0.0078	-0.0043
TPSS(D4)	0.1239	0.0083	0.0033
ωB97X(D4)	0.1111	0.0098	-0.0094
r^{2}SCAN	0.1195	0.0101	0.0073
MN15	0.1225	0.0108	-0.0108
revPBE(D4)	0.1227	0.0114	0.0112
BP86(D4)	0.1151	0.0131	0.0131
B97(D4)	0.1183	0.0138	0.0138
PBE(D4)	0.1147	0.0141	0.0141
PBEh-3c	0.1010	0.0143	0.0143
B3LYP/G(D4)	0.1064	0.0155	0.0155
r^{2}SCAN-3c	0.1209	0.0196	0.0196
GFN1-xTB	0.1174	0.0205	0.0205
OPBE(D4)	0.1422	0.0354	-0.0354
HF-3c	0.1427	0.0587	-0.0083

Table S19: Average errors per molecule calculated using density functional theory (DFT) functionals with the def2-TZVP basis set and composite methods. Errors are averaged across molecular geometries and benchmarked against experimental X-ray diffraction data.

**Molecules**	RMSE	MUE	MSE
1	0.1855	0.0256	-0.0131
2	0.3442	0.0362	0.0106
3	0.5262	0.0510	0.0209
4	0.1900	0.0370	0.0197
5	0.2328	0.0251	0.0017
6	0.1045	0.0240	-0.0060
7	0.1206	0.0240	-0.0023
8	0.1369	0.0273	-0.0126
9	0.1782	0.0234	0.0061
10	0.1669	0.1079	-0.0556
11	0.2242	0.0255	0.0010
12	1.4187	0.1618	-0.1574
13	0.0526	0.0269	0.0190
14	0.1980	0.0260	-0.0018
15	0.3034	0.0546	0.0333
16	0.2254	0.0775	-0.0116
17	0.1193	0.0169	0.0039

1.3 Organized by method

Table S20: Calculated errors obtained with GFN1-xTB method. Errors are reported in comparison to experimental X-ray diffraction data. Values are presented in ascending order of MUE.

**Compound**	RMSE	MUE	MSE
13	0.0436	0.0133	-0.0132
9	0.1711	0.0146	-0.0100
17	0.1174	0.0205	0.0205
7	0.1273	0.0250	-0.0250
1	0.0811	0.0265	-0.0265
4	0.1821	0.0360	-0.0360
11	0.2889	0.0449	-0.0396
6	0.1170	0.0486	-0.0486
5	0.2893	0.0528	-0.0528
8	0.1632	0.0542	-0.0542
14	0.5390	0.0599	-0.0583
16	0.2459	0.0867	0.0274
2	0.3844	0.1144	0.1144
3	0.5946	0.1175	0.0619
12	1.1303	0.1744	-0.1628
15	0.4844	0.2625	0.2625
10	0.1686	0.2630	0.0724

Table S21: Calculated errors obtained with HF-3c composite method. Errors are reported in comparison to experimental X-ray diffraction data. Values are presented in ascending order of MUE.

**Compound**	RMSE	MUE	MSE
8	0.1349	0.0337	0.0337
10	0.1513	0.0396	-0.0175
1	1.7443	0.0444	0.0291
7	0.1252	0.0453	0.0453
6	0.1063	0.0463	0.0463
5	0.2195	0.0481	0.0481
11	0.2332	0.0574	0.0574
17	0.1427	0.0587	-0.0083
14	0.2578	0.0616	0.0616
9	0.1828	0.0622	0.0622
15	0.3100	0.0651	-0.0563
13	0.0844	0.0814	0.0814
12	1.0476	0.0934	-0.1085
2	0.3837	0.1123	0.1123
16	0.3886	0.1236	0.0434
4	0.2494	0.1279	0.1279
3	0.5895	0.1603	0.1603

Table S22: Calculated errors obtained with PBEh-3c composite method. Errors are reported in comparison to experimental X-ray diffraction data. Values are presented in ascending order of MUE.

**Compound**	RMSE	MUE	MSE
17	0.1010	0.0143	0.0143
14	0.1838	0.0145	0.0145
11	0.2264	0.0170	0.0170
2	0.2695	0.0226	0.0123
8	0.1367	0.0252	0.0252
6	0.1177	0.0278	0.0278
1	0.0878	0.0312	-0.0240
7	0.1255	0.0315	0.0315
9	0.1873	0.0383	0.0383
3	0.5918	0.0406	0.0333
13	0.0509	0.0418	0.0418
4	0.1775	0.0422	0.0333
5	0.2848	0.0429	0.0429
15	0.2865	0.0513	0.0099
10	0.1036	0.0686	0.0409
16	0.1687	0.0911	0.0425
12	0.5423	0.1121	-0.1270

Table S23: Calculated errors obtained with r^2SCAN-3c composite method. Errors are reported in comparison to experimental X-ray diffraction data. Values are presented in ascending order of MUE.

**Compound**	RMSE	MUE	MSE
7	0.1171	0.0054	-0.0017
5	0.2196	0.0054	0.0054
14	0.1495	0.0061	-0.0026
9	0.1720	0.0078	0.0078
6	0.0965	0.0089	-0.0075
8	0.1270	0.0101	-0.0101
11	0.2153	0.0167	0.0019
13	0.0483	0.0189	0.0154
17	0.1209	0.0196	0.0196
2	0.3659	0.0220	0.0004
1	0.0799	0.0246	-0.0182
4	0.1414	0.0286	-0.0040
15	0.2740	0.0381	0.0306
3	0.5083	0.0432	0.0219
16	0.2390	0.0643	-0.0144
10	0.1493	0.0693	-0.0061
12	1.2848	0.1781	-0.2063

Table S24: Calculated errors obtained with BP86(D4) functional and def2-TZVP basis set. Errors are reported in comparison to experimental X-ray diffraction data. Values are presented in ascending order of MUE.

**Compound**	RMSE	MUE	MSE
17	0.1151	0.0131	0.0131
4	0.1463	0.0143	0.0139
9	0.1766	0.0158	-0.0158
5	0.2289	0.0160	-0.0160
13	0.0480	0.0174	0.0081
14	0.1716	0.0204	-0.0104
1	0.0811	0.0207	-0.0130
7	0.1192	0.0209	-0.0209
2	0.3672	0.0227	-0.0031
6	0.1045	0.0244	-0.0244
11	0.2023	0.0247	-0.0113
8	0.1419	0.0323	-0.0323
3	0.5493	0.0425	0.0140
15	0.2803	0.0448	0.0372
16	0.0938	0.0645	-0.0157
10	0.1613	0.0912	-0.0139
12	2.0050	0.1928	-0.2163

Table S25: Calculated errors obtained with PBE(D4) functional and def2-TZVP basis set. Errors are reported in comparison to experimental X-ray diffraction data. Values are presented in ascending order of MUE.

**Compound**	RMSE	MUE	MSE
5	0.2171	0.0113	-0.0113
4	0.1452	0.0125	0.0107
9	0.1768	0.0126	-0.0126
17	0.1147	0.0141	0.0141
7	0.1187	0.0170	-0.0170
13	0.0420	0.0182	0.0084
6	0.1016	0.0214	-0.0214
1	0.0812	0.0221	-0.0154
2	0.3672	0.0228	-0.0037
14	0.1765	0.0233	-0.0104
11	0.2073	0.0244	-0.0111
8	0.1339	0.0272	-0.0272
3	0.5257	0.0392	0.0099
15	0.2832	0.0458	0.0402
16	0.0959	0.0625	-0.0123
10	0.1540	0.1168	-0.1133
12	1.0940	0.1671	-0.1646

Table S26: Calculated errors obtained with revPBE(D4) functional and def2-TZVP basis set. Errors are reported in comparison to experimental X-ray diffraction data. Values are presented in ascending order of MUE.

**Compound**	RMSE	MUE	MSE
17	0.1227	0.0114	0.0112
4	0.1462	0.0163	0.0163
9	0.1786	0.0163	-0.0163
13	0.0517	0.0173	0.0079
5	0.2302	0.0179	-0.0179
1	0.0792	0.0196	-0.0114
2	0.2062	0.0208	0.0006
7	0.1206	0.0216	-0.0216
14	0.1816	0.0238	-0.0115
6	0.1058	0.0251	-0.0251
11	0.2107	0.0254	-0.0099
8	0.1439	0.0336	-0.0336
3	0.5126	0.0406	0.0090
15	0.2894	0.0437	0.0402
16	0.2348	0.0654	-0.0151
10	0.1657	0.1162	-0.1160
12	2.0416	0.1901	-0.2141

Table S27: Calculated errors obtained with OPBE(D4) functional and def2-TZVP basis set. Errors are reported in comparison to experimental X-ray diffraction data. Values are presented in ascending order of MUE.

**Compound**	RMSE	MUE	MSE
15	0.2837	0.0255	0.0021
13	0.0761	0.0344	-0.0091
11	0.2040	0.0347	-0.0327
6	0.1030	0.0350	-0.0350
17	0.1422	0.0354	-0.0354
9	0.1829	0.0395	-0.0395
5	0.2673	0.0456	-0.0456
1	0.0878	0.0492	-0.0492
3	0.5030	0.0545	-0.0544
2	0.3746	0.0550	-0.0550
8	0.1729	0.0552	-0.0552
7	0.1311	0.0579	-0.0579
4	0.5236	0.0589	-0.0236
14	0.2254	0.0625	-0.0625
10	0.2324	0.0884	-0.0655
16	0.2576	0.0971	-0.0615
12	2.0922	0.2370	-0.2622

Table S28: Calculated errors obtained with B97(D4) functional and def2-TZVP basis set. Errors are reported in comparison to experimental X-ray diffraction data. Values are presented in ascending order of MUE.

**Compound**	RMSE	MUE	MSE
17	0.1183	0.0138	0.0138
4	0.1434	0.0205	0.0168
13	0.0488	0.0208	0.0084
1	0.0790	0.0212	-0.0147
9	0.1721	0.0219	-0.0219
2	0.3665	0.0230	-0.0044
14	0.1717	0.0234	-0.0145
5	0.2322	0.0236	-0.0236
6	0.1035	0.0241	-0.0241
7	0.1211	0.0259	-0.0259
11	0.2128	0.0265	-0.0097
15	0.2934	0.0357	0.0272
8	0.1439	0.0377	-0.0377
3	0.5523	0.0499	0.0072
16	0.2274	0.0683	-0.0187
10	0.1793	0.1120	-0.1120
12	2.0320	0.1982	-0.2227

Table S29: Calculated errors obtained with B3LYP/G(D4) functional and def2-TZVP basis set. Errors are reported in comparison to experimental X-ray diffraction data. Values are presented in ascending order of MUE.

**Compound**	RMSE	MUE	MSE
8	0.1164	0.0108	0.0108
6	0.1051	0.0131	0.0131
17	0.1064	0.0155	0.0155
11	0.2308	0.0162	0.0162
1	0.0827	0.0192	-0.0001
7	0.1190	0.0203	0.0203
2	0.3644	0.0250	0.0246
5	0.2234	0.0270	0.0270
9	0.1734	0.0271	0.0271
13	0.0443	0.0348	0.0348
14	0.1985	0.0350	0.0350
4	0.1674	0.0401	0.0366
15	0.2983	0.0420	0.0262
3	0.5516	0.0523	0.0408
10	0.1108	0.0611	0.0335
16	0.0941	0.0636	-0.0086
12	1.0860	0.1317	-0.1282

Table S30: Calculated errors obtained with r^2SCAN functional and def2-TZVP basis set. Errors are reported in comparison to experimental X-ray diffraction data. Values are presented in ascending order of MUE.

**Compound**	RMSE	MUE	MSE
5	0.2021	0.0024	-0.0024
9	0.1723	0.0053	-0.0016
14	0.1447	0.0085	-0.0085
7	0.1155	0.0090	-0.0088
17	0.1195	0.0101	0.0073
6	0.0979	0.0141	-0.0141
13	0.0381	0.0143	0.0079
11	0.2153	0.0163	-0.0103
8	0.1203	0.0166	-0.0166
2	0.3662	0.0232	-0.0063
4	0.1483	0.0252	-0.0111
1	0.0800	0.0268	-0.0219
15	0.2838	0.0390	0.0245
3	0.4987	0.0398	0.0147
16	0.2398	0.0713	-0.0262
10	0.1784	0.1178	-0.1178
12	0.9862	0.1572	-0.1603

Table S31: Calculated errors obtained with TPSS(D4) functional and def2-TZVP basis set. Errors are reported in comparison to experimental X-ray diffraction data. Values are presented in ascending order of MUE.

**Compound**	RMSE	MUE	MSE
17	0.1239	0.0083	0.0033
14	0.1671	0.0092	-0.0089
9	0.1793	0.0126	-0.0126
5	0.2291	0.0137	-0.0137
13	0.0522	0.0139	0.0092
1	0.0808	0.0192	-0.0084
7	0.1194	0.0195	-0.0195
6	0.1015	0.0220	-0.0220
11	0.2093	0.0227	-0.0090
4	0.1503	0.0234	0.0197
2	0.3670	0.0237	-0.0076
8	0.1424	0.0290	-0.0290
3	0.5044	0.0329	0.0033
15	0.2824	0.0366	0.0263
16	0.2470	0.0701	-0.0276
10	0.1625	0.0771	-0.0233
12	2.0983	0.1791	-0.2117

Table S32: Calculated errors obtained with MN15 functional and def2-TZVP basis set. Errors are reported in comparison to experimental X-ray diffraction data. Values are presented in ascending order of MUE.

**Compound**	RMSE	MUE	MSE
14	0.1200	0.0049	-0.0008
8	0.1360	0.0062	-0.0001
6	0.0955	0.0069	0.0031
7	0.1180	0.0099	0.0098
17	0.1225	0.0108	-0.0108
11	0.2300	0.0112	0.0097
5	0.2118	0.0146	0.0146
9	0.1850	0.0158	0.0158
13	0.0656	0.0164	0.0164
1	0.0805	0.0181	-0.0027
3	0.4521	0.0206	0.0032
2	0.3636	0.0233	-0.0031
4	0.1755	0.0357	0.0098
15	0.3130	0.0427	0.0235
16	0.3123	0.0845	-0.0389
12	1.1096	0.1408	-0.1469
10	0.1802	0.1453	-0.1453

Table S33: Calculated errors obtained with revM11 functional and def2-TZVP basis set. Errors are reported in comparison to experimental X-ray diffraction data. Values are presented in ascending order of MUE.

**Compound**	RMSE	MUE	MSE
17	0.1083	0.0078	-0.0043
14	0.1391	0.0178	0.0178
3	0.4555	0.0206	0.0001
1	0.0802	0.0232	-0.0135
2	0.2319	0.0240	-0.0078
11	0.2445	0.0270	0.0270
8	0.1265	0.0287	0.0287
6	0.0999	0.0310	0.0310
13	0.0519	0.0369	0.0369
7	0.1211	0.0370	0.0370
15	0.2902	0.0372	0.0170
4	0.1895	0.0373	0.0373
9	0.1831	0.0441	0.0441
5	0.2088	0.0442	0.0442
16	0.3165	0.0796	-0.0082
10	0.1823	0.1423	-0.1423
12	1.9294	0.1430	-0.1754

Table S34: Calculated errors obtained with TPSSh(D4) functional and def2-TZVP basis set. Errors are reported in comparison to experimental X-ray diffraction data. Values are presented in ascending order of MUE.

**Compound**	RMSE	MUE	MSE
5	0.2300	0.0042	-0.0042
9	0.1768	0.0055	-0.0030
17	0.1221	0.0074	-0.0024
14	0.1771	0.0078	-0.0069
7	0.1152	0.0100	-0.0100
6	0.1014	0.0149	-0.0149
13	0.0468	0.0153	0.0153
11	0.2132	0.0182	-0.0036
8	0.1337	0.0203	-0.0203
1	0.0810	0.0209	-0.0113
2	0.3659	0.0237	-0.0079
4	0.1572	0.0290	0.0239
3	0.5061	0.0304	0.0025
15	0.2844	0.0328	0.0161
10	0.1476	0.0659	-0.0106
16	0.2619	0.0747	-0.0320
12	1.1233	0.1648	-0.1583

Table S35: Calculated errors obtained with ωB97X(D4) functional and def2-TZVP basis set. Errors are reported in comparison to experimental X-ray diffraction data. Values are presented in ascending order of MUE.

**Compound**	RMSE	MUE	MSE
17	0.1111	0.0098	-0.0094
8	0.1166	0.0165	0.0165
6	0.1150	0.0206	0.0206
2	0.3631	0.0214	0.0036
1	0.0813	0.0222	-0.0082
11	0.2434	0.0240	0.0240
7	0.1151	0.0270	0.0270
15	0.3172	0.0310	0.0050
3	0.5235	0.0315	0.0063
5	0.2303	0.0326	0.0326
13	0.0482	0.0348	0.0348
9	0.1815	0.0351	0.0351
14	0.1645	0.0376	0.0376
4	0.1968	0.0437	0.0437
16	0.1832	0.0731	-0.0193
12	1.0962	0.1286	-0.1278
10	0.2428	0.1522	-0.1522

Table S36: Average errors per method calculated. Errors are averaged across molecular geometries and benchmarked against experimental X-ray diffraction data.

**Methods**	RMSE	MUE	MSE
GFN1-xTB	0.3017	0.0832	0.0019
HF-3c	0.3736	0.0742	0.0423
PBEh-3c	0.2142	0.0419	0.0161
r^2SCAN-3c	0.2535	0.0334	-0.0099
BP86(D4)	0.2937	0.0399	-0.0180
PBE(D4)	0.2374	0.0387	-0.0198
revPBE(D4)	0.2954	0.0415	-0.0240
OPBE(D4)	0.3447	0.0627	-0.0554
B97(D4)	0.3057	0.0439	-0.0269
B3LYP/G(D4)	0.2396	0.0373	0.0132
r^2SCAN	0.2357	0.0351	-0.0207
TPSS(D4)	0.3069	0.0361	-0.0195
MN15	0.2512	0.0357	-0.0143
revM11	0.2917	0.0460	-0.0018
TPSSh(D4)	0.2496	0.0321	-0.0134
ωB97X(D4)	0.2547	0.0436	-0.0018

2 TD-DFT Calculations

2.1 Template for calculations

All TD-DFT calculations were carried out using the following template for ORCA input files:

```
! "Functional" def2-TZVP

! CPCM("Solvent")


%tddft

nroots 40

maxdim 5

end


%scf maxiter 800 end

%maxcore 8000

%pal nprocs 8 end


*xyzfile "optimized_geometry_solvent.xyz" C S


# C stands for the charge of the complex

# S stands for the spin multiplicity of the complex
```

2.2 Organized by molecule

Table S37: Calculated errors for the TD-DFT spectra of compound 1 in comparison with experimental UV-Vis data. Calculations were made simulating a gaseous medium.

**Method**	shift (eV)	FWHM (eV)	sim (%)
B97	-0.77	0.85	99.3
B3LYP/G	-0.74	0.85	99.3
O3LYP	-0.56	0.92	99.2
PBE0	-0.94	0.86	98.9
r^2SCAN	-0.76	0.96	98.7
revM06L	-1.45	1.14	96.1
TPSS	-0.40	1.03	98.6
MN15	-0.95	0.93	99.2
revM11	-1.42	0.86	94.6
TPSSh	-0.65	0.93	99.3
ωPBE	-0.92	0.86	99.0
CAM-B3LYP	-1.14	0.82	96.9
ωB97X	-1.30	0.89	95.3

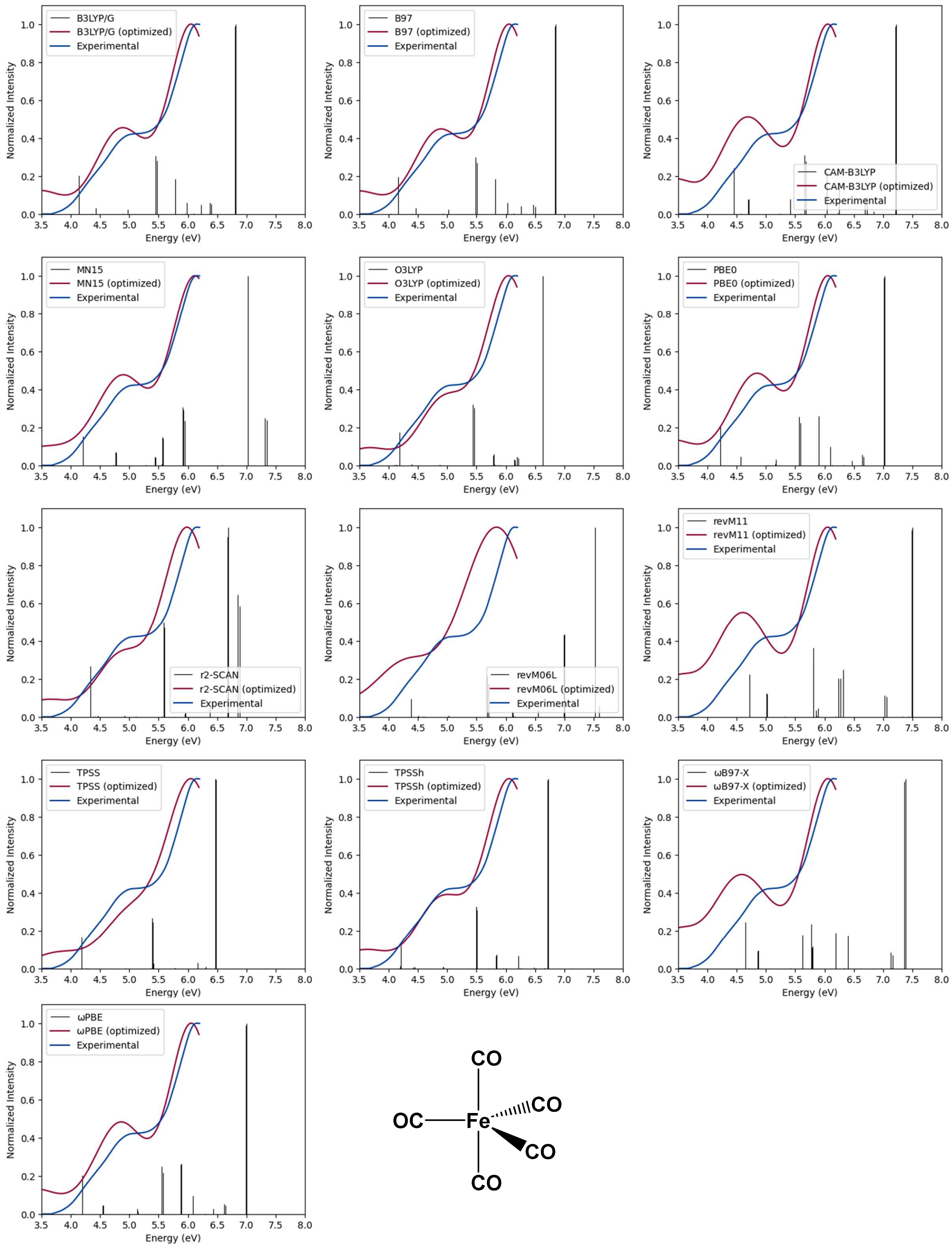

Figure S1: Calculated and optimized TD-DFT spectra of compound 1 in comparison with experimental UV-Vis data. Calculations were made simulating a gaseous medium.

Table S38: Calculated errors for the TD-DFT spectra of compound 2 in comparison with experimental UV-Vis data. Calculations were made in isopentane using the CPCM model.

**Method**	shift (eV)	FWHM (eV)	sim (%)
B97	0.39	0.60	98.7
B3LYP/G	0.40	0.60	98.6
O3LYP	0.71	0.72	99.0
PBE0	0.13	0.57	98.9
r^2SCAN	-0.82	0.72	99.0
revM06L	0.62	0.73	99.1
TPSS	-0.28	0.73	97.9
MN15	0.04	0.70	98.8
revM11	-0.59	0.63	98.5
TPSSh	-0.76	0.79	99.5
ωPBE	0.17	0.57	98.9
CAM-B3LYP	-0.15	0.57	98.3
ωB97X	-0.37	0.55	98.2

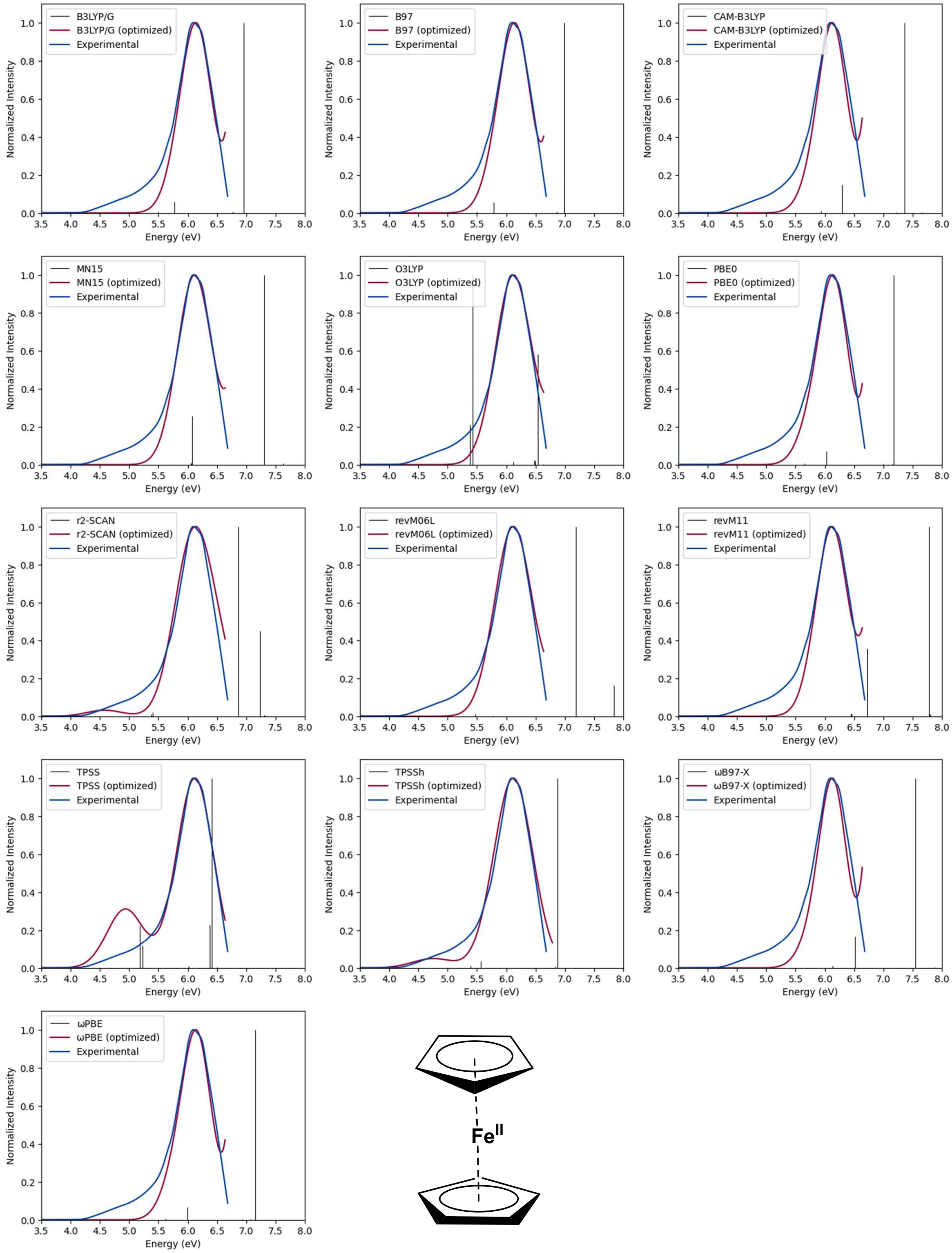

Figure S2: Calculated and optimized TD-DFT spectra of compound 2 in comparison with experimental UV-Vis data. Calculations were made in isopentane using the CPCM model.

Table S39: Calculated errors for the TD-DFT spectra of compound 3 in comparison with experimental UV-Vis data. Calculations were made in DMSO using the CPCM model.

**Method**	shift (eV)	FWHM (eV)	sim (%)
B97	-0.34	0.93	99.1
B3LYP/G	-0.32	0.99	99.1
O3LYP	-0.19	0.39	97.9
PBE0	-0.77	0.69	97.7
r^2SCAN	0.29	0.65	99.6
revM06L	-0.06	0.84	99.8
TPSS	0.45	0.59	99.6
MN15	-0.81	0.84	98.1
revM11	-2.22	0.60	98.7
TPSSh	-0.26	0.34	97.6
ωPBE	-0.64	0.66	97.7
CAM-B3LYP	-1.27	0.87	97.8
ωB97X	-2.10	0.48	96.2

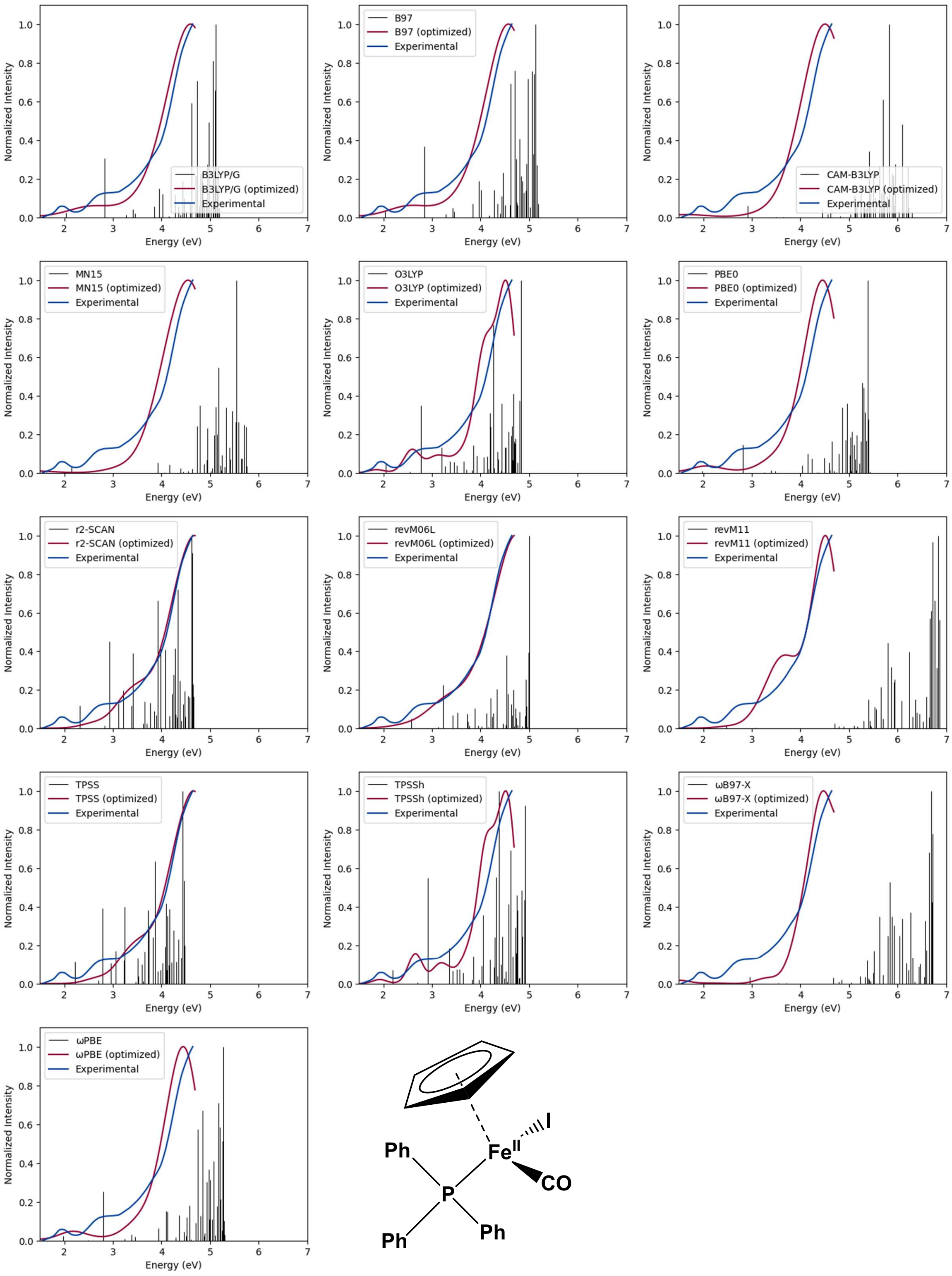

Figure S3: Calculated and optimized TD-DFT spectra of compound 3 in comparison with experimental UV-Vis data. Calculations were made in DMSO using the CPCM model.

Table S40: Calculated errors for the TD-DFT spectra of compound 4 in comparison with experimental UV-Vis data. Calculations were made in ACN using the CPCM model.

**Method**	shift (eV)	FWHM (eV)	sim (%)
B97	-0.39	0.83	69.6
B3LYP/G	-0.37	1.18	68.9
O3LYP	-0.29	0.63	74.1
PBE0	-0.44	1.18	67.4
r^2SCAN	-0.25	1.18	75.6
revM06L	-0.42	1.18	75.6
TPSS	-0.12	1.18	76.3
MN15	-0.46	1.18	64.8
revM11	-0.91	1.18	63.2
TPSSh	-0.35	0.67	73.2
ωPBE	-0.42	1.18	67.8
CAM-B3LYP	-0.59	1.18	64.5
ωB97X	-0.79	1.18	61.7

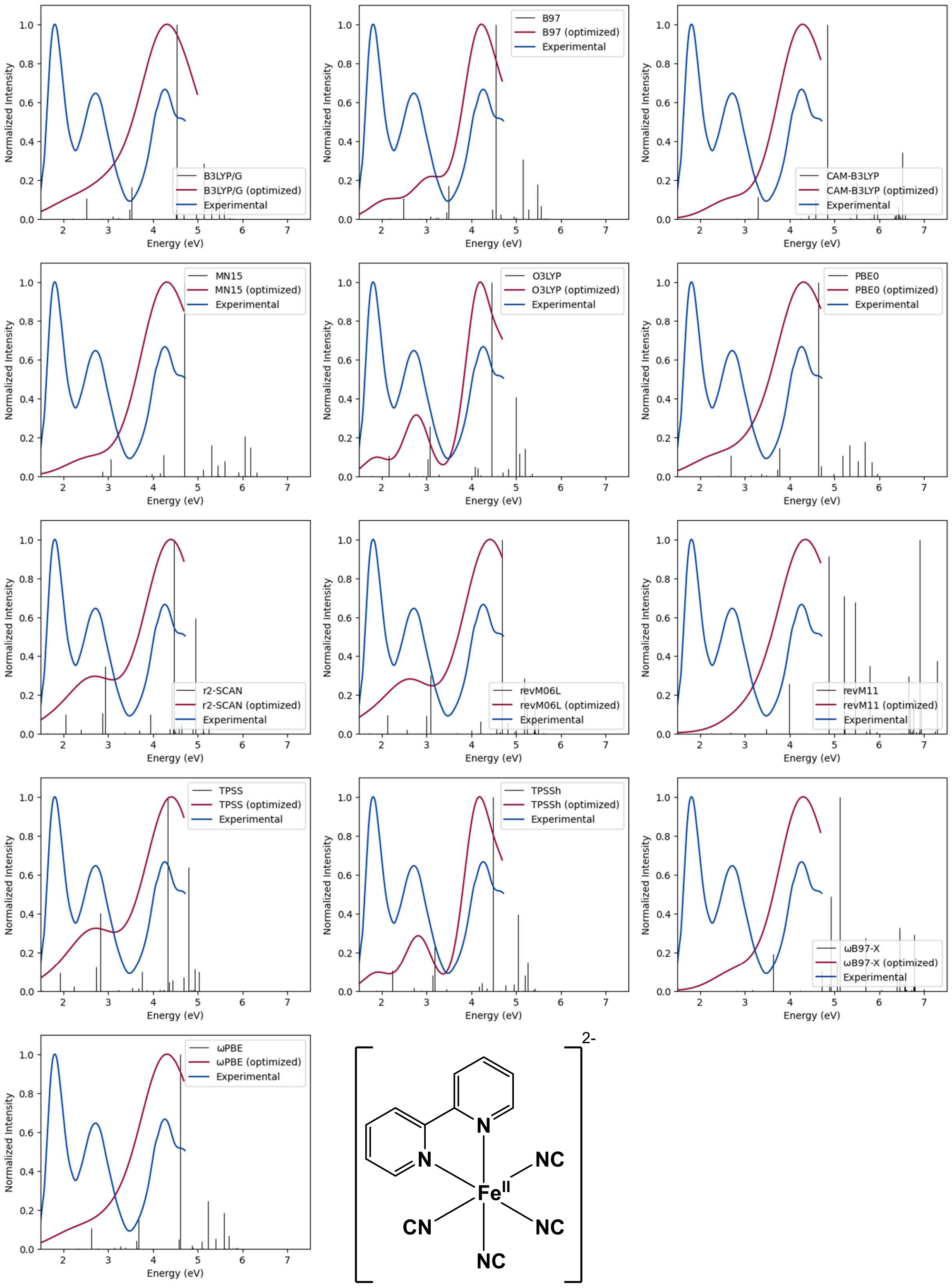

Figure S4: Calculated and optimized TD-DFT spectra of compound 4 in comparison with experimental UV-Vis data. Calculations were made in DMSO using the ACN model.

Table S41: Calculated errors for the TD-DFT spectra of compound 5 in comparison with experimental UV-Vis data. Calculations were made in ACN using the CPCM model.

**Method**	shift (eV)	FWHM (eV)	sim (%)
B97	-0.25	0.58	96.1
B3LYP/G	-0.23	0.61	95.5
O3LYP	-0.12	0.57	86.9
PBE0	-0.32	0.70	95.1
r^2SCAN	0.95	0.61	86.5
revM06L	-0.39	0.72	96.1
TPSS	1.09	0.58	87.6
MN15	-0.50	0.67	93.2
revM11	-0.60	0.44	90.8
TPSSh	-0.17	0.56	88.7
ωPBE	-0.30	0.68	95.0
CAM-B3LYP	-0.73	0.61	92.2
ωB97X	-0.55	0.37	85.5

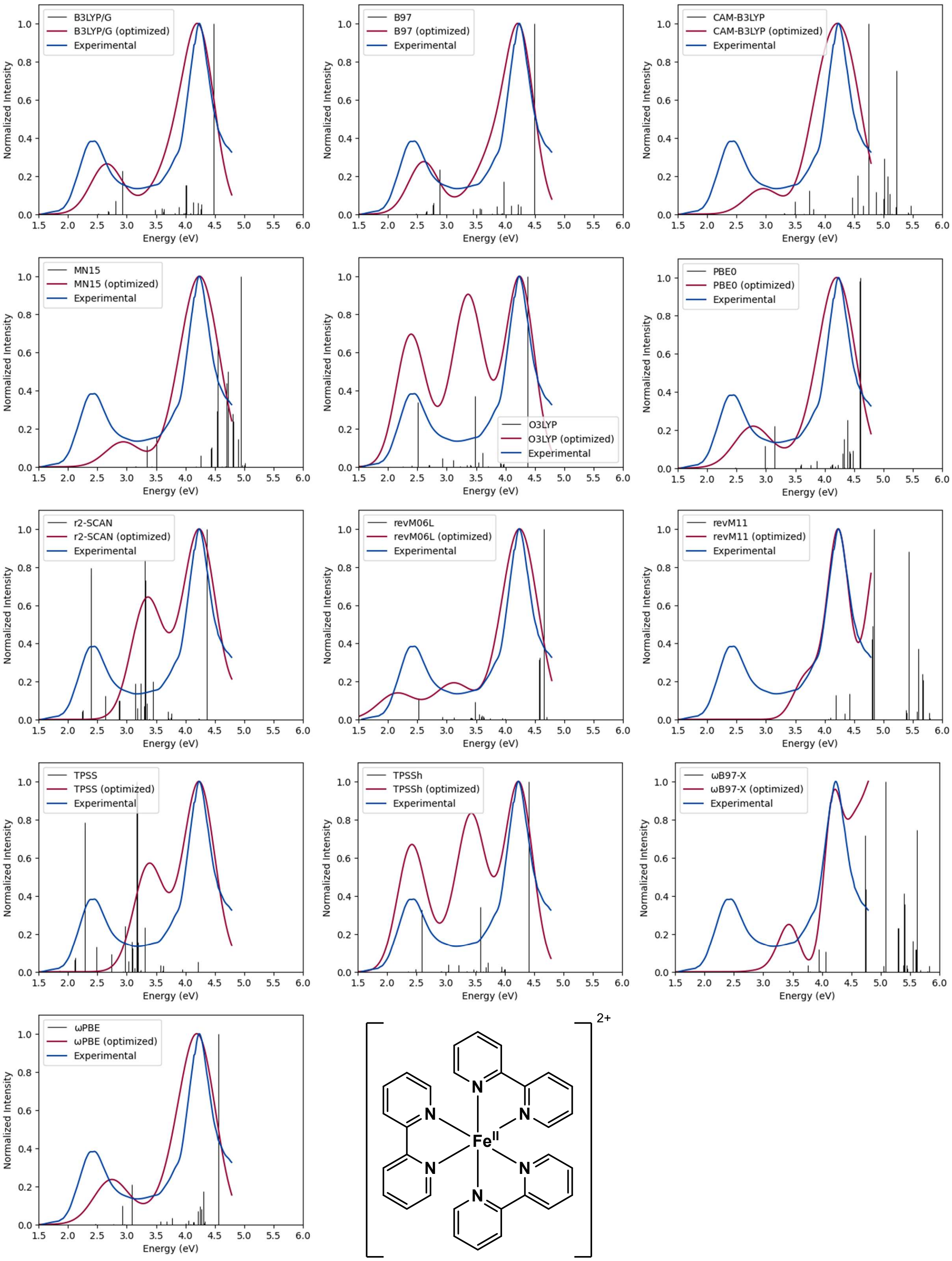

Figure S5: Calculated and optimized TD-DFT spectra of compound 5 in comparison with experimental UV-Vis data. Calculations were made in ACN using the CPCM model.

Table S42: Calculated errors for the TD-DFT spectra of compound 6 in comparison with experimental UV-Vis data. Calculations were made in ACN using the CPCM model.

**Method**	shift (eV)	FWHM (eV)	sim (%)
B97	-0.23	1.18	83.1
B3LYP/G	-0.23	1.18	82.8
O3LYP	-0.03	1.18	82.3
PBE0	-0.41	1.18	82.2
r^2SCAN	0.04	1.09	81.5
revM06L	-0.16	1.07	82.7
TPSS	0.15	1.18	80.4
MN15	-0.34	1.18	82.8
revM11	-0.78	0.41	83.2
TPSSh	-0.07	1.18	82.7
ωPBE	-0.35	1.18	81.7
CAM-B3LYP	-0.52	1.18	82.6
ωB97X	-0.71	0.40	82.4

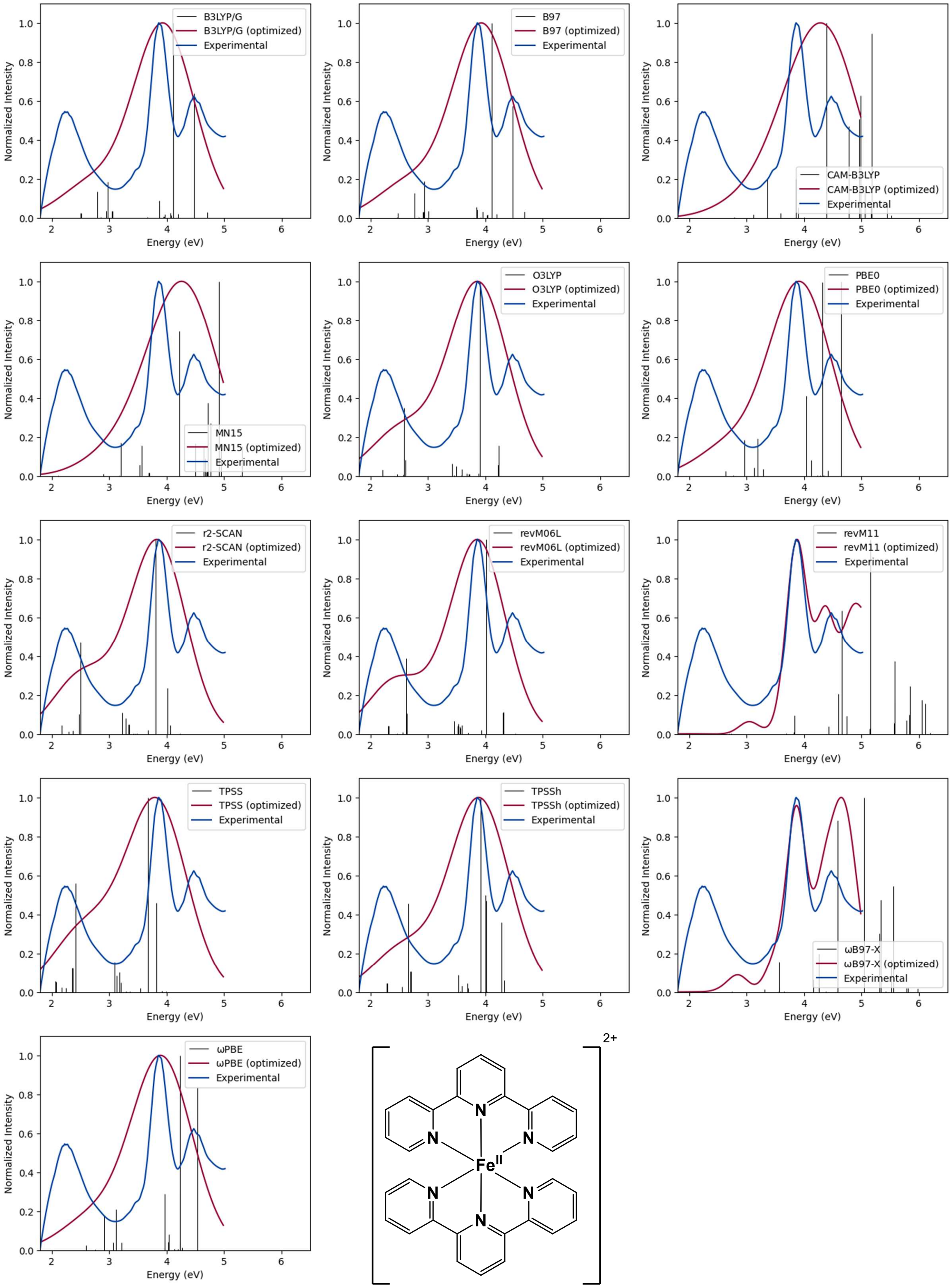

Figure S6: Calculated and optimized TD-DFT spectra of compound 6 in comparison with experimental UV-Vis data. Calculations were made in ACN using the CPCM model.

Table S43: Calculated errors for the TD-DFT spectra of compound 7 in comparison with experimental UV-Vis data. Calculations were made in ACN using the CPCM model.

**Method**	shift (eV)	FWHM (eV)	sim (%)
B97	-0.09	0.81	93.3
B3LYP/G	-0.08	0.58	93.3
O3LYP	0.03	1.01	88.8
PBE0	-0.21	0.49	95.1
r^2SCAN	0.01	1.17	80.6
revM06L	-0.11	0.84	86.5
TPSS	0.15	1.16	80.2
MN15	-0.37	0.48	97.3
revM11	-0.83	0.88	96.0
TPSSh	0.01	0.99	89.9
ωPBE	-0.15	0.49	93.8
CAM-B3LYP	-0.53	0.53	96.5
ωB97X	-0.74	0.82	95.7

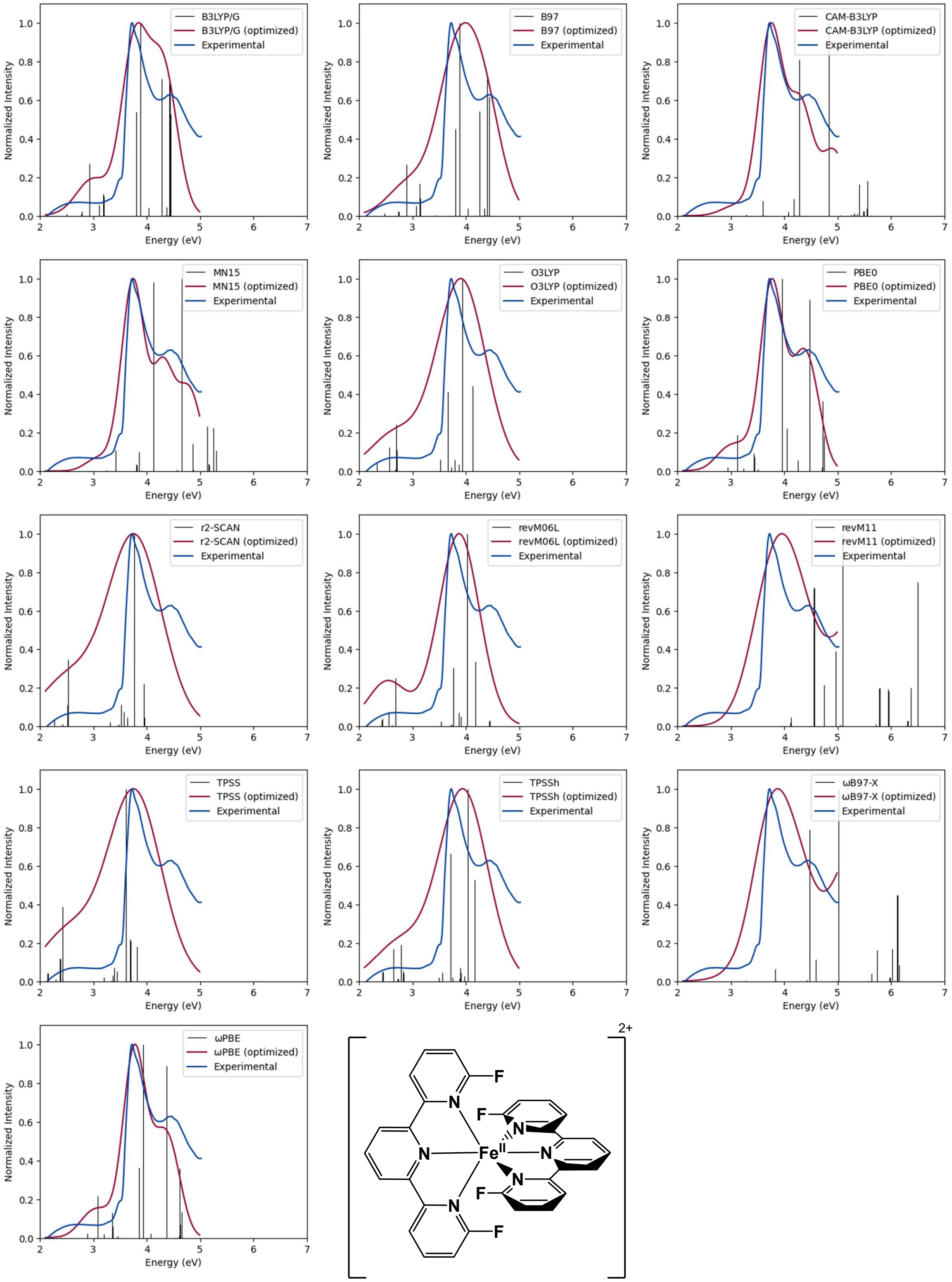

Figure S7: Calculated and optimized TD-DFT spectra of compound 7 in comparison with experimental UV-Vis data. Calculations were made in ACN using the CPCM model.

Table S44: Calculated errors for the TD-DFT spectra of compound 8 in comparison with experimental UV-Vis data. Calculations were made in H_2O using the CPCM model.

**Method**	shift (eV)	FWHM (eV)	sim (%)
B97	-0.43	1.18	33.8
B3LYP/G	-0.47	1.18	33.9
O3LYP	-0.08	1.18	32.1
PBE0	0.29	1.18	49.5
r^2SCAN	0.08	1.18	34.6
revM06L	-0.05	1.18	36.6
TPSS	0.18	1.18	32.7
MN15	-0.15	0.52	82.8
revM11	-0.67	0.45	94.7
TPSSh	-0.15	1.18	33.9
ωPBE	-0.62	1.18	33.7
CAM-B3LYP	-0.65	0.57	95.7
ωB97X	-0.62	0.96	93.3

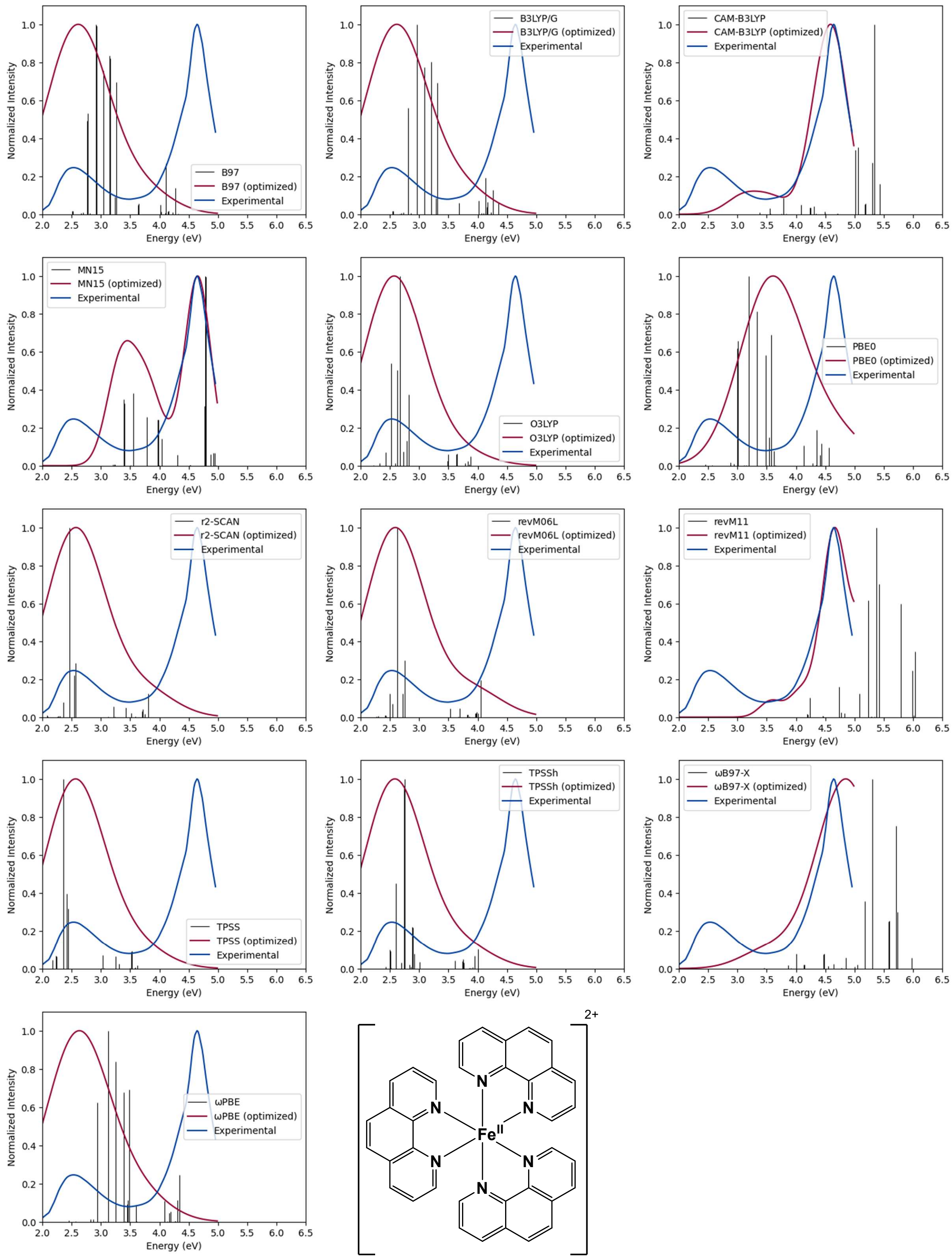

Figure S8: Calculated and optimized TD-DFT spectra of compound 8 in comparison with experimental UV-Vis data. Calculations were made in H_2O using the CPCM model.

Table S45: Calculated errors for TD-DFT spectra of compound 9 in comparison with experimental UV-Vis data. Calculations were made in ACN using the CPCM model.

**Method**	shift (eV)	FWHM (eV)	sim (%)
B97	0.23	1.18	86.4
B3LYP/G	0.24	1.06	87.2
O3LYP	0.97	0.53	98.3
PBE0	0.85	0.63	89.1
r^2SCAN	1.10	0.64	97.7
revM06L	0.93	0.64	97.5
TPSS	1.20	0.63	96.4
MN15	-0.20	0.69	92.4
revM11	-0.48	0.62	92.2
TPSSh	0.91	0.46	98.7
ωPBE	0.89	1.18	90.0
CAM-B3LYP	-0.17	0.97	91.2
ωB97X	-0.39	0.43	93.8

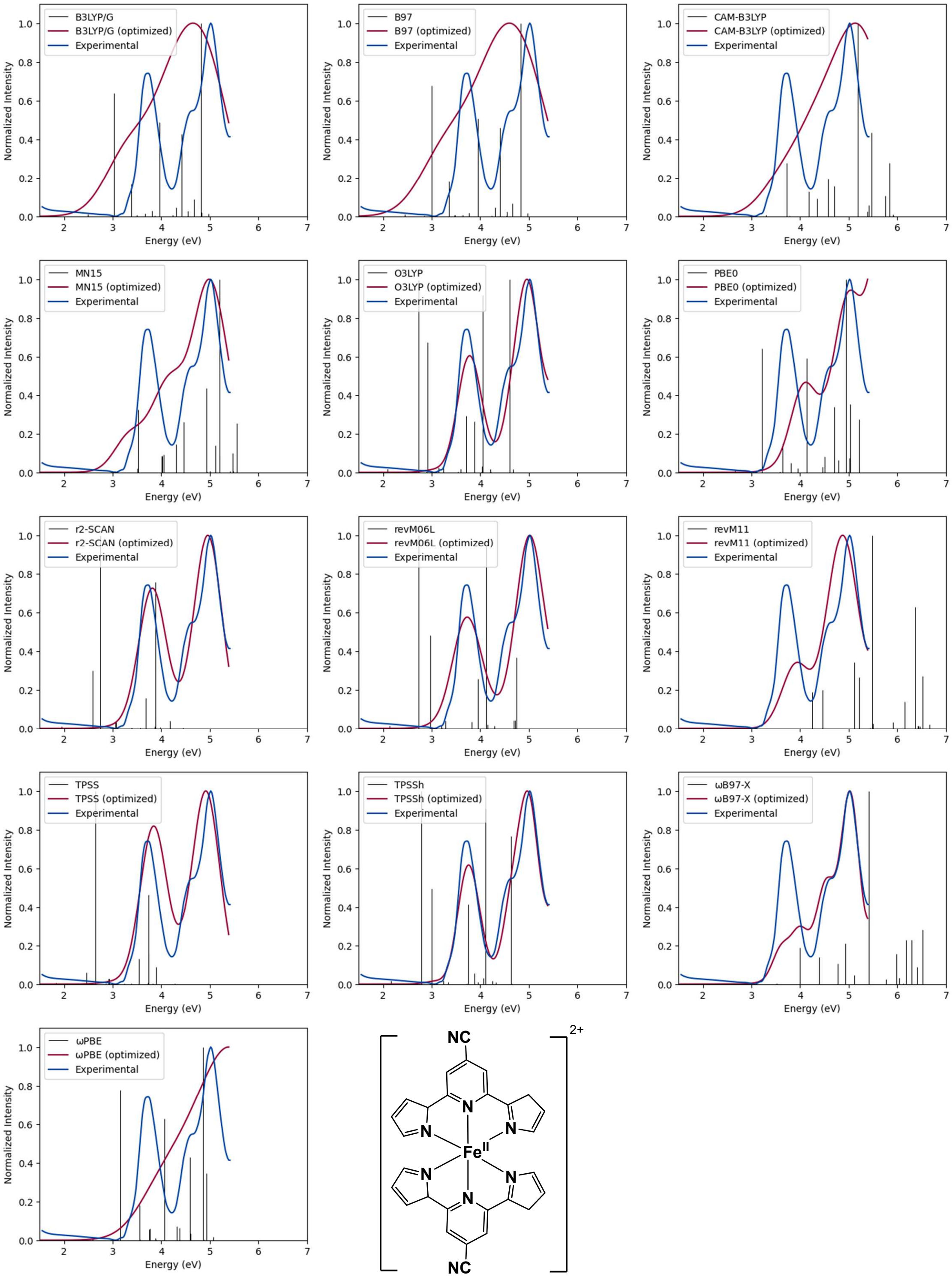

Figure S9: Calculated and optimized TD-DFT spectra of compound 9 in comparison with experimental UV-Vis data. Calculations were made in ACN using the CPCM model.

Table S46: Calculated errors for the TD-DFT spectra of compound 10 in comparison with experimental UV-Vis data. Calculations were made in ACN using the CPCM model.

**Method**	shift (eV)	FWHM (eV)	sim (%)
B97	0.71	0.64	98.4
B3LYP/G	0.64	0.62	98.1
O3LYP	1.00	0.64	97.0
PBE0	0.35	0.63	98.3
r^2SCAN	0.95	0.59	97.3
revM06L	1.04	0.49	96.0
TPSS	0.98	0.59	96.6
MN15	-0.44	0.73	71.8
revM11	-3.06	1.18	56.6
TPSSh	0.90	0.60	97.1
ωPBE	0.53	0.64	98.0
CAM-B3LYP	-0.61	0.60	59.9
ωB97X	-1.49	0.30	46.8

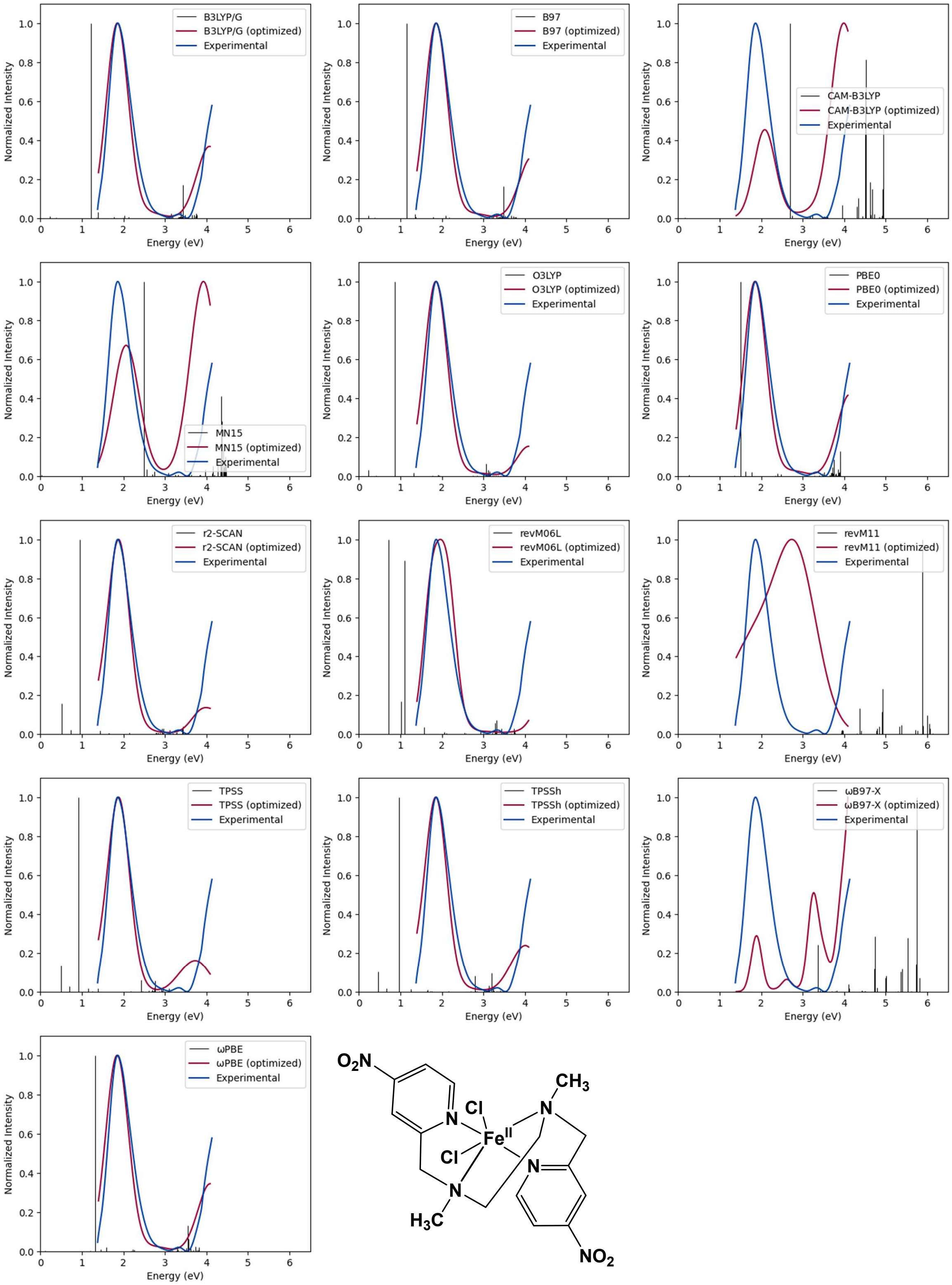

Figure S10: Calculated and optimized TD-DFT spectra of compound 10 in comparison with experimental UV-Vis data. Calculations were made in ACN using the CPCM model.

Table S47: Calculated errors for the TD-DFT spectra of compound 11 in comparison with experimental UV-Vis data. Calculations were made in ACN using the CPCM model.

**Method**	shift (eV)	FWHM (eV)	sim (%)
B97	-0.05	0.44	90.0
B3LYP/G	-0.05	0.44	90.1
O3LYP	0.13	0.52	89.6
PBE0	-0.20	0.43	90.8
r^2SCAN	0.25	0.53	90.0
revM06L	-0.09	0.54	90.4
TPSS	0.44	1.18	90.2
MN15	-0.31	0.43	91.7
revM11	-0.68	1.18	89.1
TPSSh	0.11	0.49	89.9
ωPBE	-0.72	1.18	88,1
CAM-B3LYP	-0.50	0.44	92.4
ωB97X	-0.70	0.43	91.9

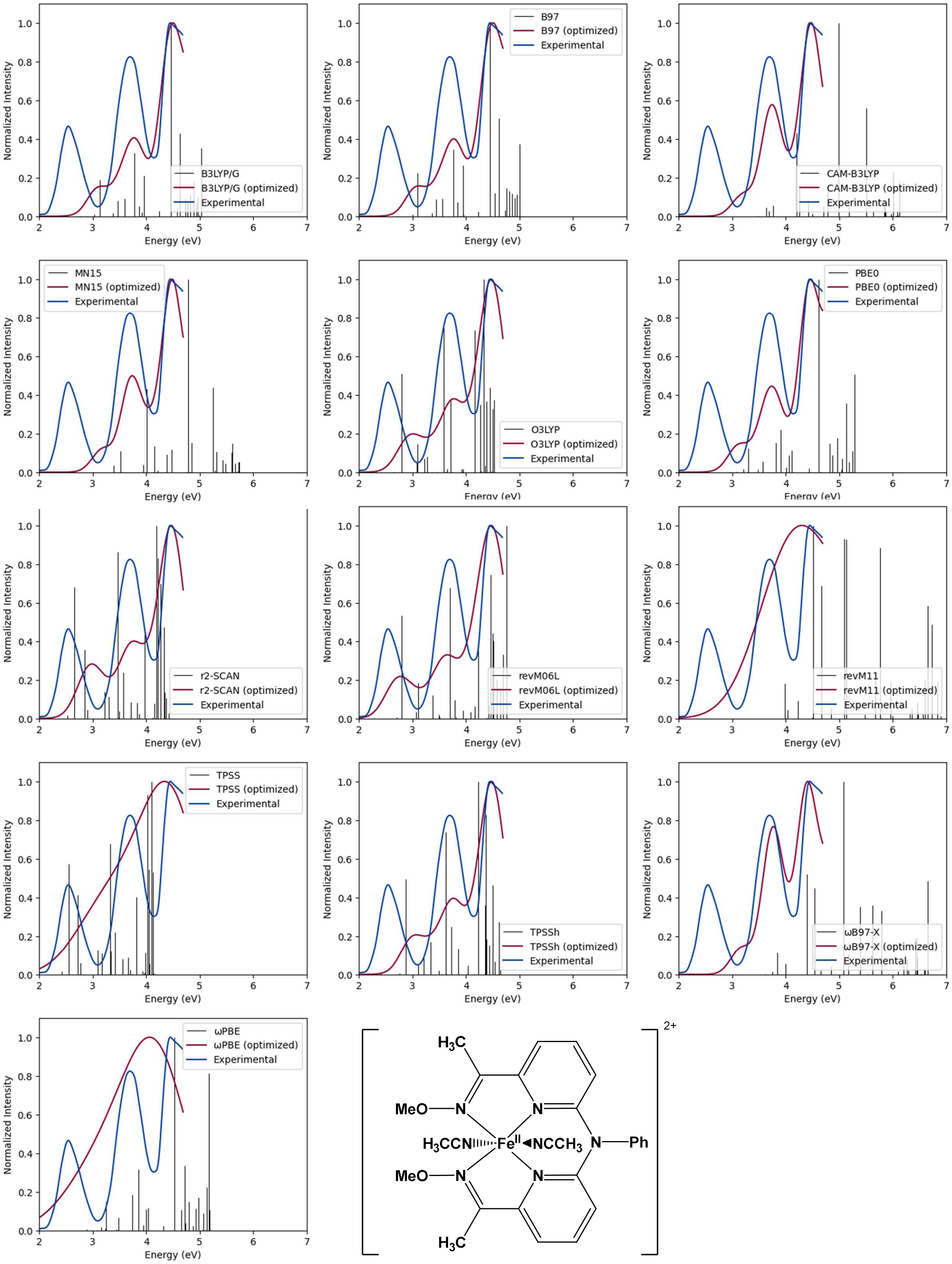

Figure S11: Calculated and optimized TD-DFT spectra of compound 11 in comparison with experimental UV-Vis data. Calculations were made in ACN using the CPCM model.

Table S48: Calculated errors for the TD-DFT spectra of compound 12 in comparison with experimental UV-Vis data. Calculations were made in DCM using the CPCM model.

**Method**	shift (eV)	FWHM (eV)	sim (%)
B97	-0.20	0.42	97.5
B3LYP/G	-0.22	0.44	97.7
O3LYP	0.26	0.46	97.7
PBE0	-0.37	0.44	98.1
r^2SCAN	0.38	0.50	97.4
revM06L	0.03	0.46	95.2
TPSS	0.58	0.51	98.0
MN15	-0.31	0.79	99.2
revM11	-0.45	0.66	98.3
TPSSh	0.12	0.43	97.5
ωPBE	-0.31	0.43	98.7
CAM-B3LYP	-0.64	0.71	98.8
ωB97X	-0.49	0.51	97.0

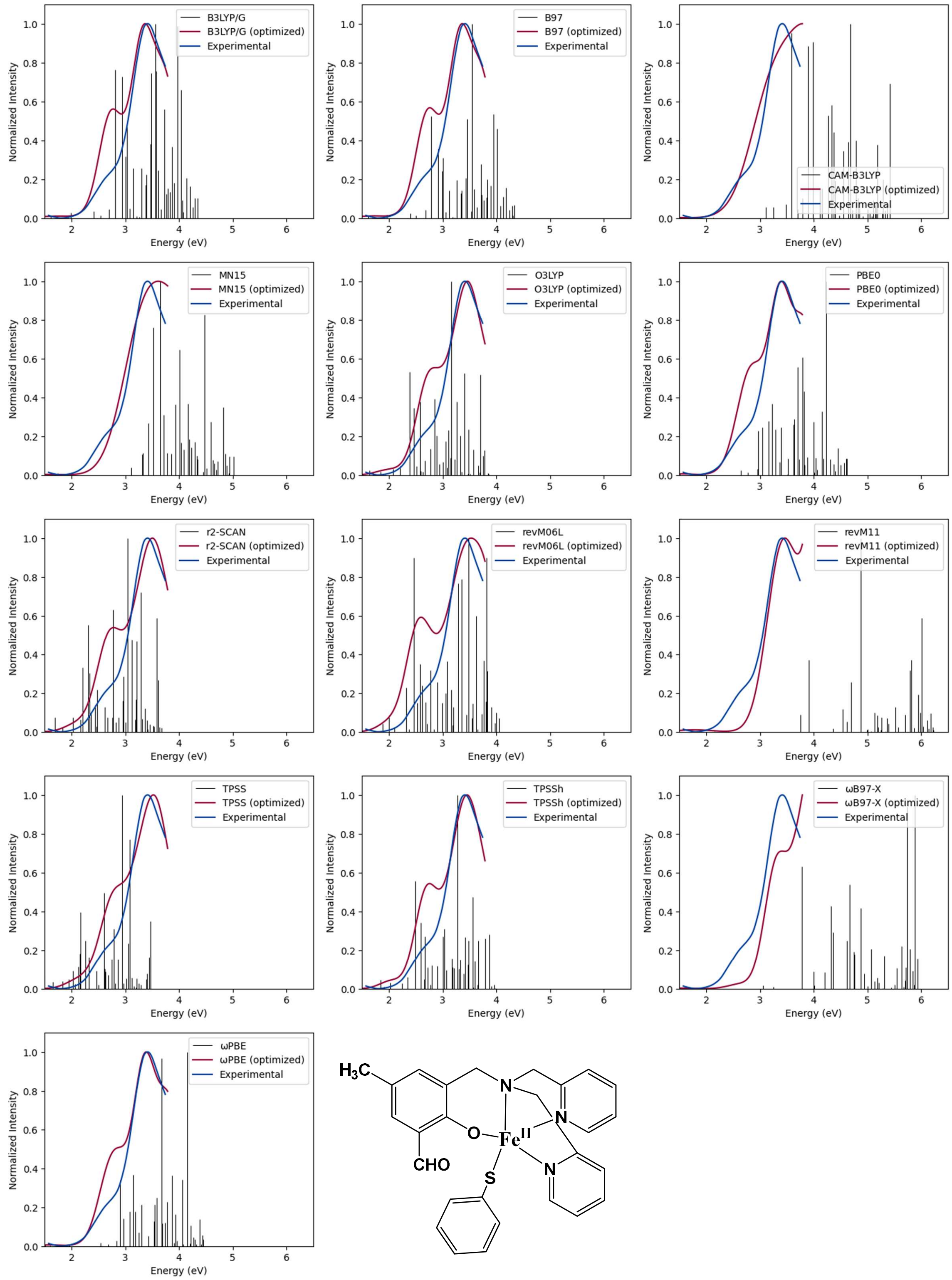

Figure S12: Calculated and optimized TD-DFT spectra of compound 12 in comparison with experimental UV-Vis data. Calculations were made in DCM using the CPCM model.

Table S49: Calculated errors for the TD-DFT spectra of compound 13 in comparison with experimental UV-Vis data. Calculations were made in H_2O using the CPCM model.

**Method**	shift (eV)	FWHM (eV)	sim (%)
B97	-0.51	0.35	91.1
B3LYP/G	-0.56	0.31	90.1
O3LYP	0.03	0.47	92.1
PBE0	-0.24	0.39	73.0
r^2SCAN	0.28	0.45	92.4
revM06L	-0.01	0.44	92.5
TPSS	0.46	0.44	92.2
MN15	1.23	0.50	92.5
revM11	0.71	0.59	91.0
TPSSh	-0.09	0.46	92.2
ωPBE	-0.14	0.35	72.5
CAM-B3LYP	0.07	0.40	73.2
ωB97X	0.10	0.67	78.6

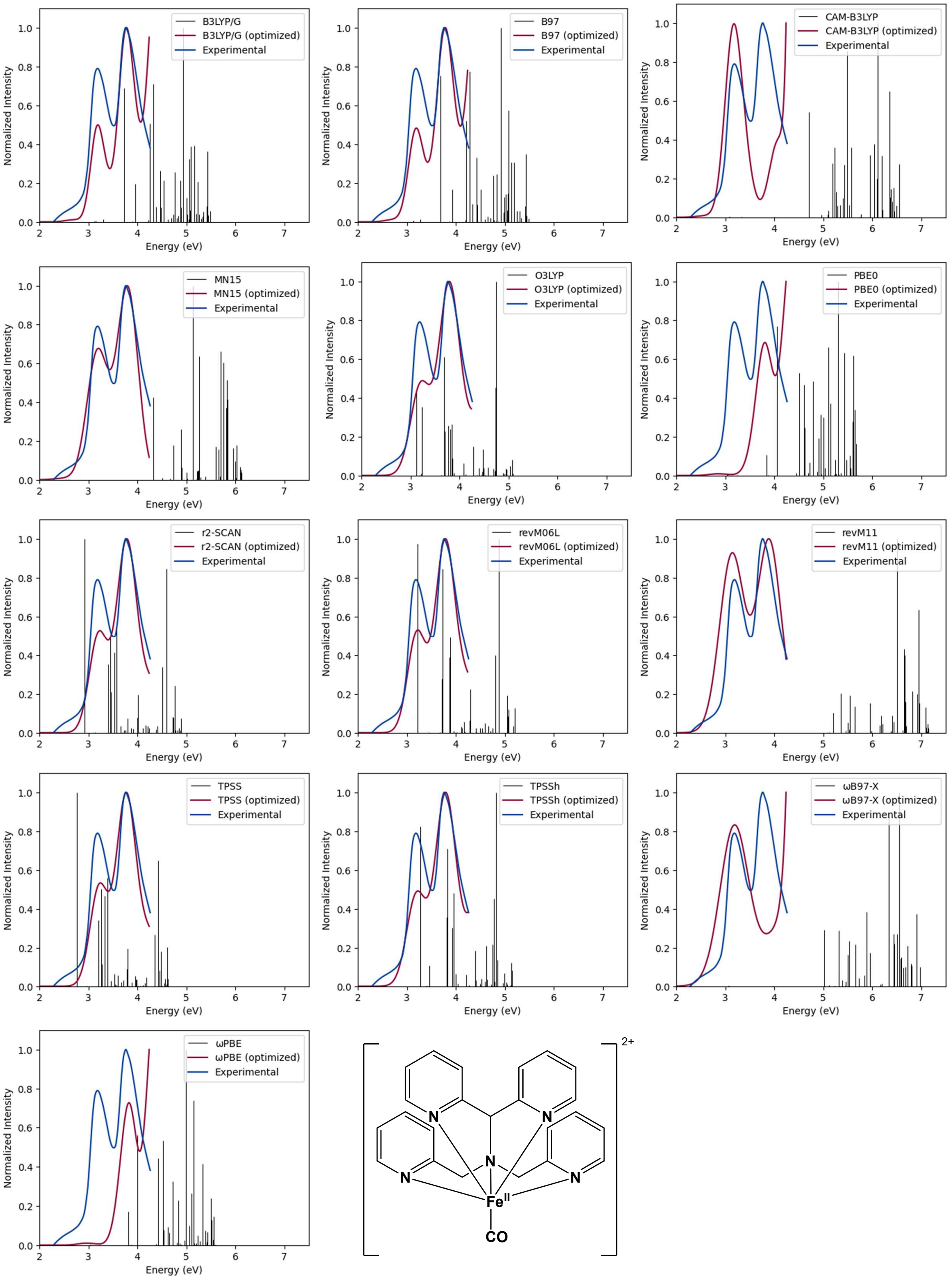

Figure S13: Calculated and optimized TD-DFT spectra of compound 13 in comparison with experimental UV-Vis data. Calculations were made in H_2O using the CPCM model.

Table S50: Calculated errors for the TD-DFT spectra of compound 14 in comparison with experimental UV-Vis data. Calculations were made in DCM using the CPCM model.

**Method**	shift (eV)	FWHM (eV)	sim (%)
B97	-0.64	0.30	96.8
B3LYP/G	-0.66	0.30	96.8
O3LYP	-0.48	0.29	96.2
PBE0	-0.77	0.30	96.9
r^2SCAN	-0.46	0.31	93.9
revM06L	-0.53	0.30	96.2
TPSS	-0.38	0.31	93.9
MN15	-0.88	0.30	96.9
revM11	-1.32	0.30	96.8
TPSSh	-0.54	0.29	96.1
ωPBE	-0.75	0.30	96.8
CAM-B3LYP	-1.00	0.30	96.9
ωB97X	-1.11	0.30	96.9

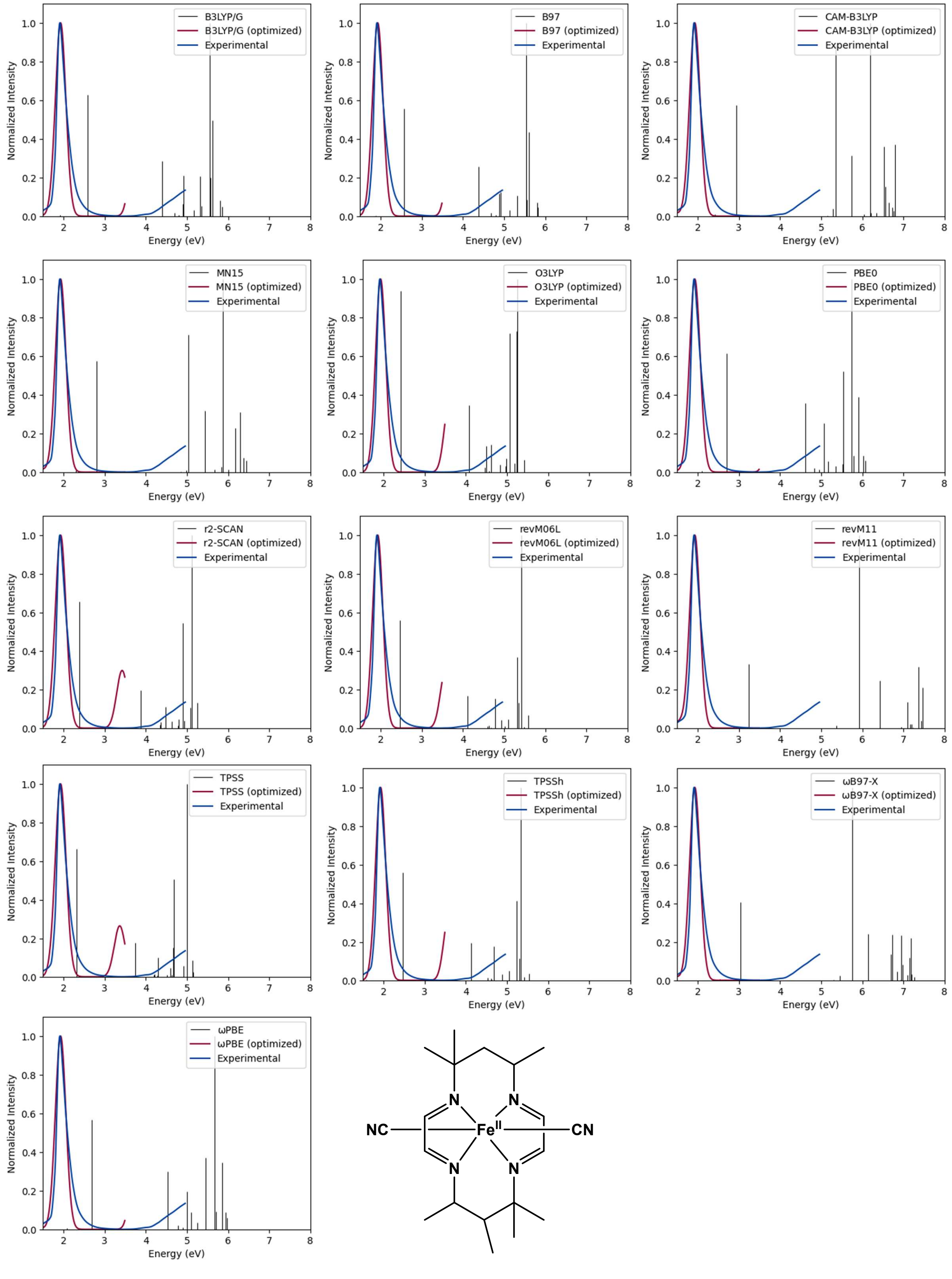

Figure S14: Calculated and optimized TD-DFT spectra of compound 14 in comparison with experimental UV-Vis data. Calculations were made in DCM using the CPCM model.

Table S51: Calculated errors for the TD-DFT spectra of compound 15 in comparison with experimental UV-Vis data. Calculations were made in H_2O using the CPCM model.

**Method**	shift (eV)	FWHM (eV)	sim (%)
B97	0.17	0.64	71.8
B3LYP/G	0.20	0.65	71.3
O3LYP	0.43	0.78	72.1
PBE0	-0.09	0.61	71.1
r^2SCAN	0.52	0.83	73.3
revM06L	0.20	0.87	73.0
TPSS	0.64	0.87	72.7
MN15	0.33	0.51	70.2
revM11	-1.12	1.18	66.5
TPSSh	0.39	0.79	72.0
ωPBE	0.05	0.61	72.2
CAM-B3LYP	-0.37	1.18	66.3
ωB97X	-0.52	1.18	64.2

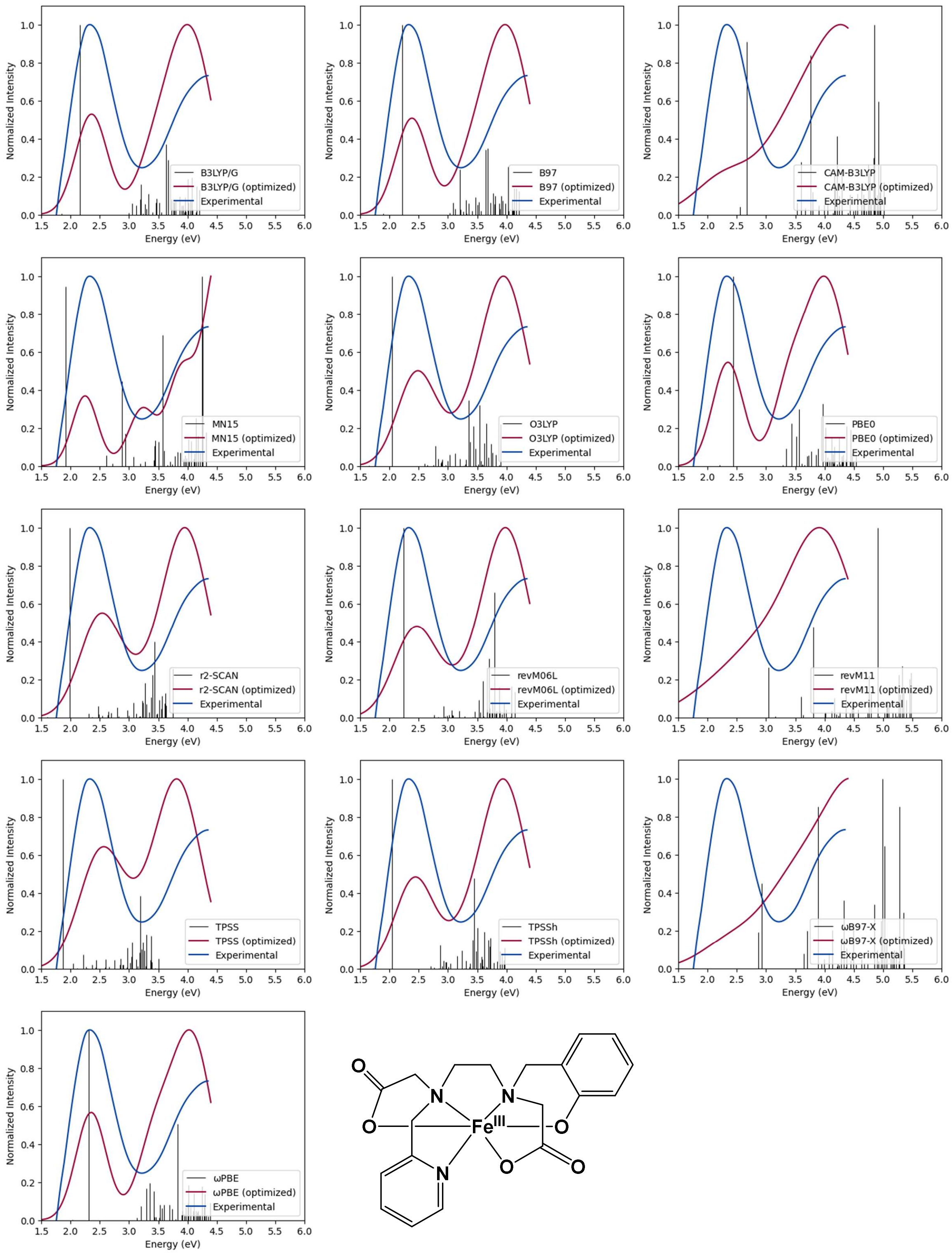

Figure S15: Calculated and optimized TD-DFT spectra of compound 15 in comparison with experimental UV-Vis data. Calculations were made in H_2O using the CPCM model.

Table S52: Calculated errors for the TD-DFT spectra of compound 16 in comparison with experimental UV-Vis data. Calculations were made in CH_3OH using the CPCM model.

**Method**	shift (eV)	FWHM (eV)	sim (%)
B97	-1.76	0.76	92.6
B3LYP/G	-1.69	0.86	92.5
O3LYP	-1.45	1.18	90.3
PBE0	-1.80	1.18	92.2
r^2SCAN	-1.80	1.18	89.2
revM06L	-2.08	0.75	93.2
TPSS	-2.28	1.18	68.4
MN15	-1.10	1.18	88.2
revM11	-1.21	0.37	93.6
TPSSh	-1.93	1.18	90.0
ωPBE	-1.77	0.98	92.4
CAM-B3LYP	-2.04	1.18	91.0
ωB97X	-1.61	0.38	91.1

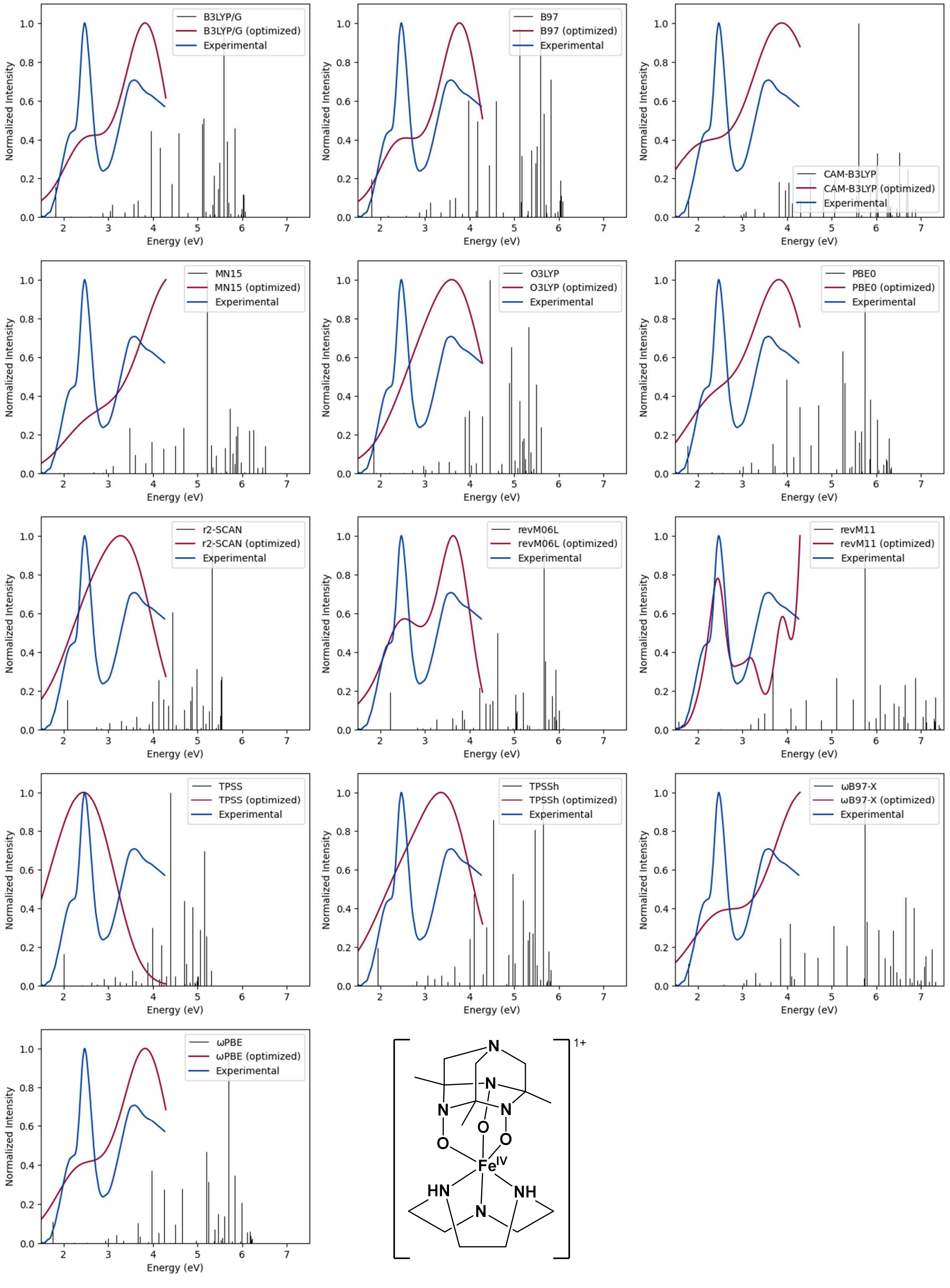

Figure S16: Calculated and optimized TD-DFT spectra of compound 16 in comparison with experimental UV-Vis data. Calculations were made in CH_3OH using the CPCM model.

Table S53: Calculated errors for the TD-DFT spectra of compound 17 in comparison with experimental UV-Vis data. Calculations were made in H_2O using the CPCM model.

**Method**	shift (eV)	FWHM (eV)	sim (%)
B97	-0.42	0.37	78.9
B3LYP/G	-0.42	0.38	77.3
O3LYP	-0.34	0.34	74.9
PBE0	-0.53	0.33	83.9
r^2SCAN	0.09	1.18	56.7
revM06L	-0.62	0.34	74.9
TPSS	0.21	1.18	56.2
MN15	-0.11	0.40	68.5
revM11	-0.60	0.60	74.2
TPSSh	-0.50	0.30	72.2
ωPBE	-0.46	0.35	82.9
CAM-B3LYP	-0.69	0.36	84.2
ωB97X	-0.78	0.35	84.3

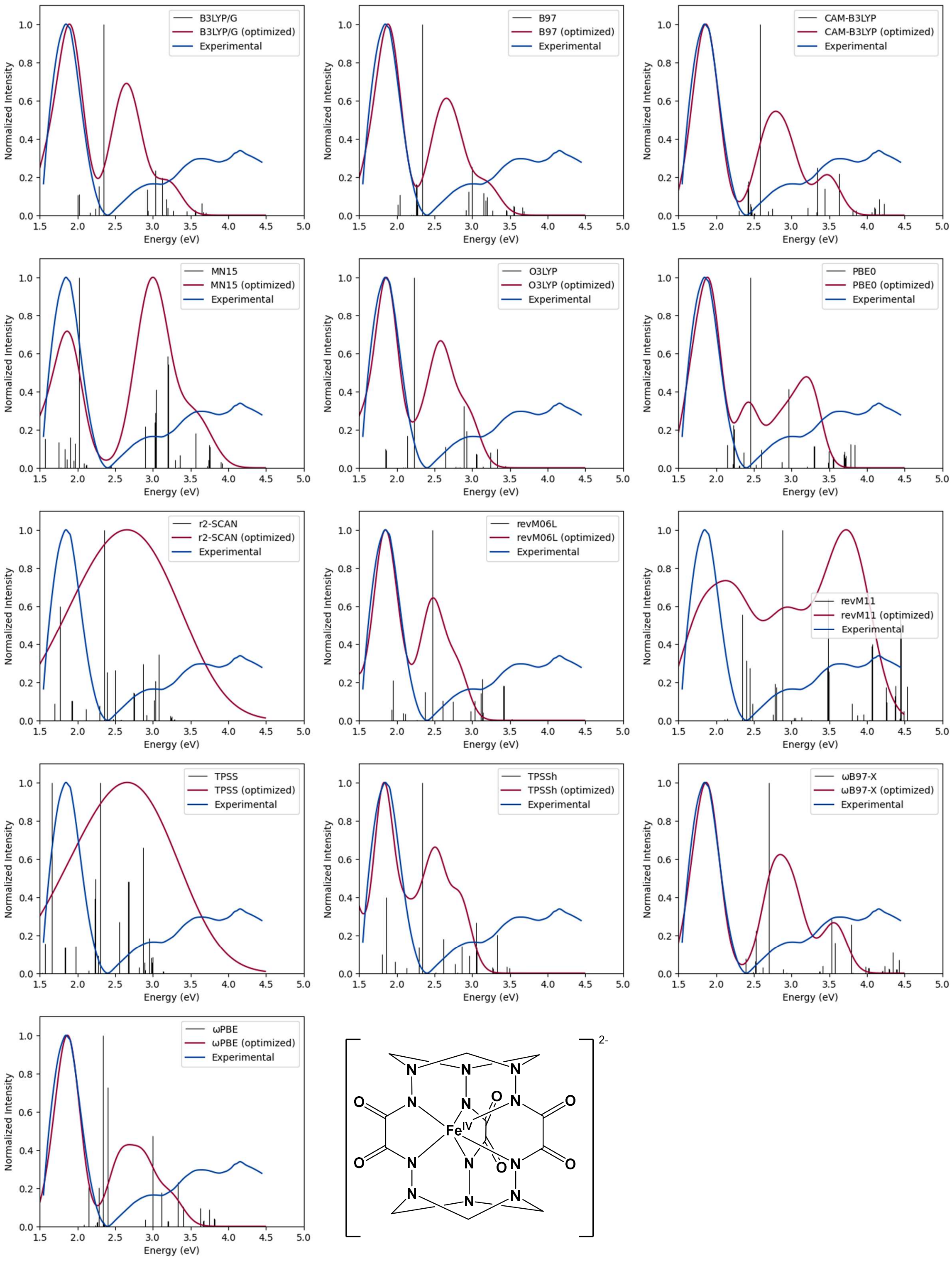

Figure S17: Calculated and optimized TD-DFT spectra of compound 17 in comparison with experimental UV-Vis data. Calculations were made in H_2O using the CPCM model.

Table S54: Calculated average errors for TD-DFT spectra of all compounds in comparison with experimental UV-Vis data.

**Compound**	Absolute Shift (eV)	FWHM (eV)	sim (%)
1	0.92	0.92	98.0
2	0.42	0.65	98.7
3	0.75	0.68	98.4
4	0.45	1.07	69.4
5	0.48	0.59	91.5
6	0.30	1.06	82.4
7	0.26	0.79	91.3
8	0.34	1.01	52.9
9	0.66	0.81	93.2
10	0.98	0.63	85.5
11	0.31	0.62	90.5
12	0.34	0.52	97.8
13	0.34	0.45	86.4
14	0.73	0.30	96.2
15	0.39	0.82	70.5
16	1.73	0.95	89.6
17	0.44	0.50	74.5

2.3 Organized by method

Table S55: Calculated errors for the TD-DFT spectra of all compounds obtained with the TPSS/def2-TZVP method in comparison with experimental UV-Vis data.

**Compound**	shift (eV)	FWHM (eV)	sim (%)
1	-0.40	1.03	98.6
2	-0.28	0.73	97.9
3	0.45	0.59	99.6
4	-0.12	1.18	76.3
5	1.09	0.58	87.6
6	0.15	1.18	80.4
7	0.15	1.16	80.2
8	0.18	1.18	32.7
9	1.20	0.63	96.4
10	0.98	0.59	96.6
11	0.44	1.18	90.2
12	0.58	0.51	98.0
13	0.46	0.44	92.2
14	-0.38	0.31	93.9
15	0.64	0.87	72.7
16	-2.28	1.18	68.4
17	0.21	1.18	56.2

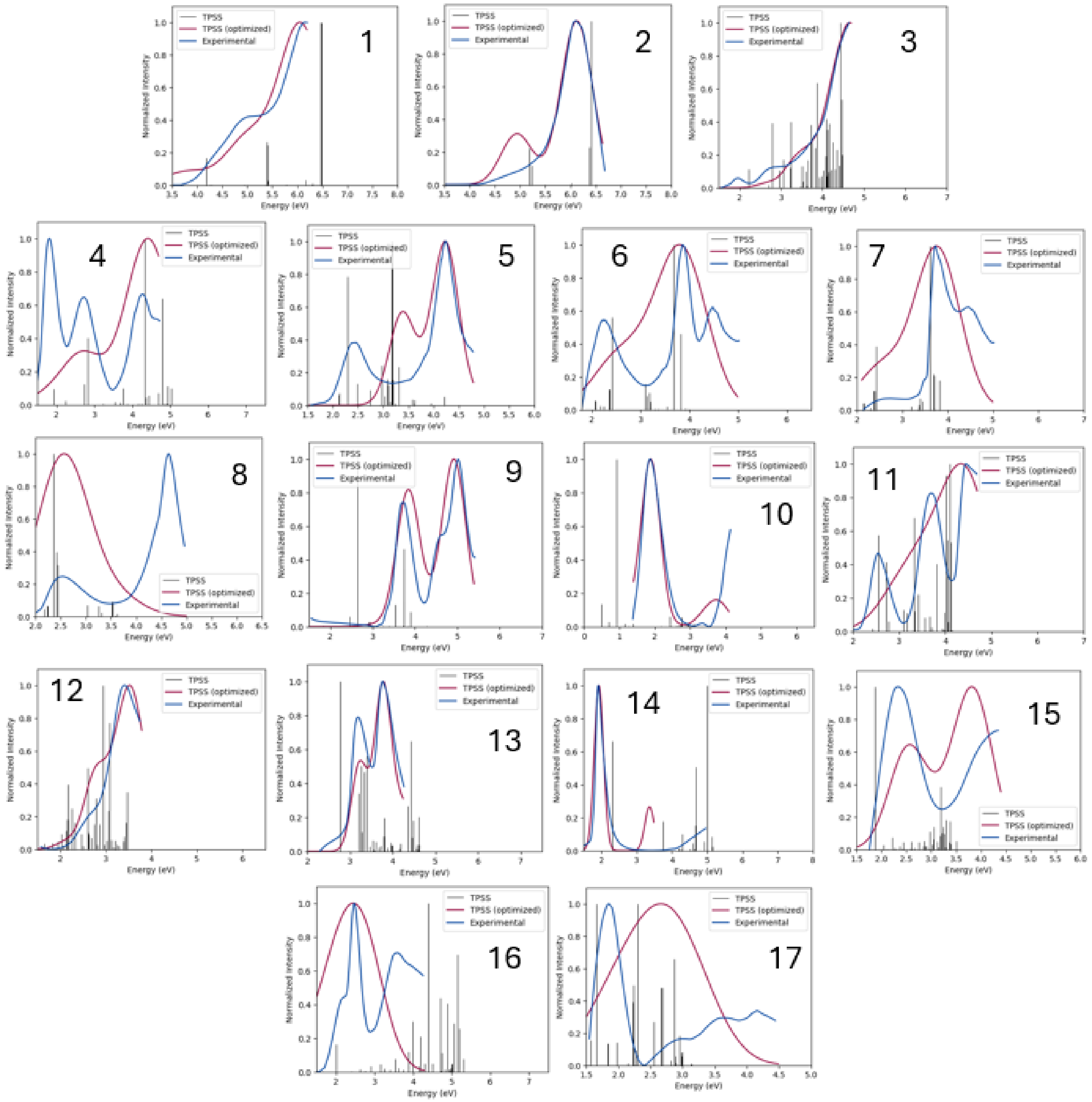

Figure S18: Calculated and optimized TPSS/def2-TZVP TD-DFT spectra for all compounds in comparison with experimental UV-Vis data. Labels correspond to the respective compound.

Table S56: Calculated errors for the TD-DFT spectra of all compounds obtained with the r^2SCAN/def2-TZVP method in comparison with experimental UV-Vis data.

**Compound**	shift (eV)	FWHM (eV)	sim (%)
1	-0.76	0.96	98.7
2	-0.82	0.72	99.0
3	0.29	0.65	99.6
4	-0.25	1.18	75.6
5	0.95	0.61	86.5
6	0.04	1.09	81.5
7	0.01	1.17	80.6
8	0.08	1.18	34.6
9	1.10	0.64	97.7
10	0.95	0.59	97.3
11	0.25	0.53	90.0
12	0.38	0.50	97.4
13	0.28	0.45	92.4
14	-0.46	0.31	93.9
15	0.52	0.83	73.3
16	-1.80	1.18	89.2
17	0.09	1.18	56.7

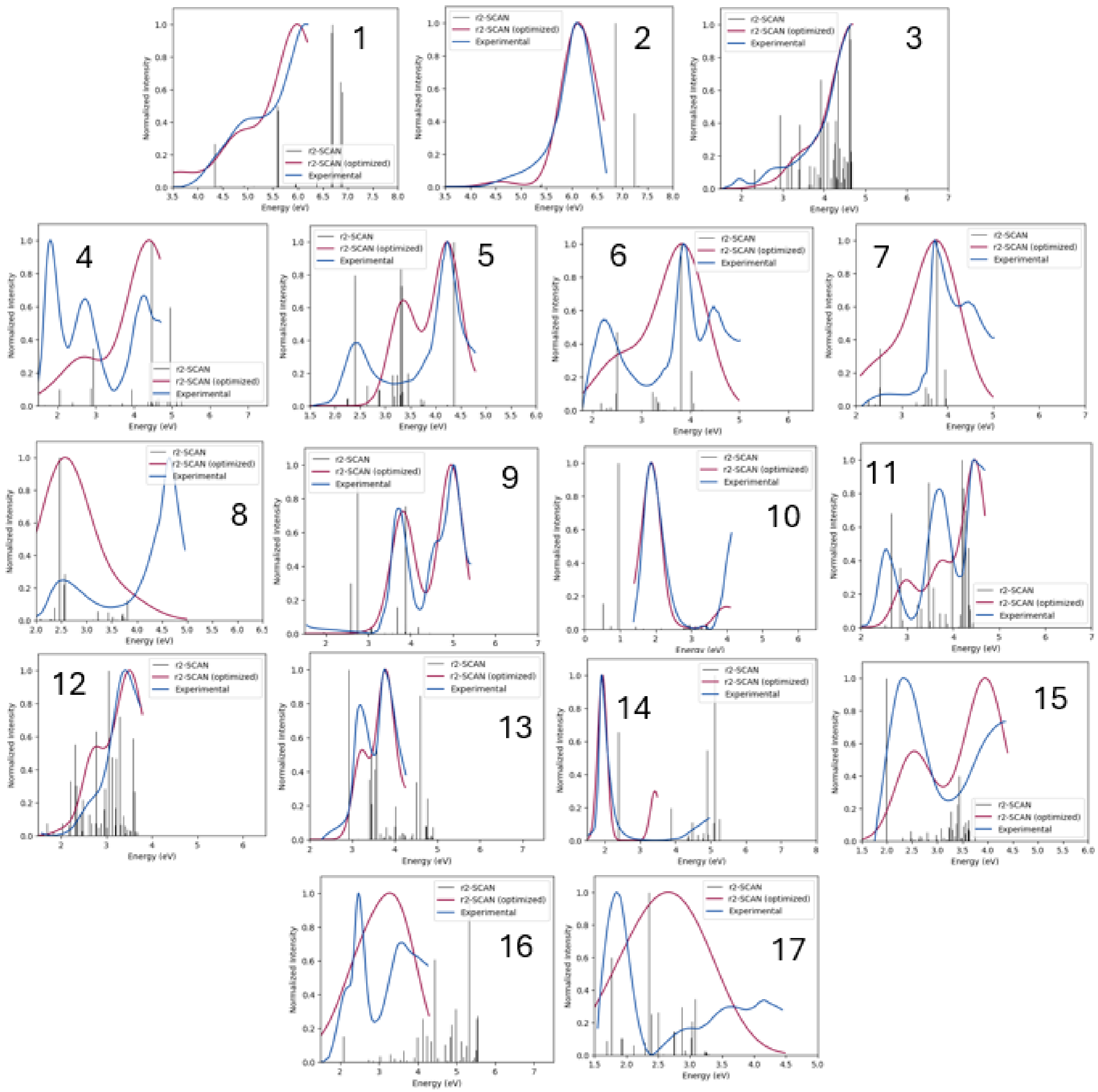

Figure S19: Calculated and optimized r^{2}SCAN/def2-TZVP TD-DFT spectra for all compounds in comparison with experimental UV-Vis data. Labels correspond to the respective compound.

Table S57: Calculated errors for the TD-DFT spectra of all compounds obtained with the revM06L/def2-TZVP method in comparison with experimental UV-Vis data.

**Compound**	shift (eV)	FWHM (eV)	sim (%)
1	-1.45	1.14	96.1
2	0.62	0.73	99.1
3	-0.06	0.84	99.8
4	-0.42	1.18	75.6
5	-0.39	0.72	96.1
6	-0.16	1.07	82.7
7	-0.11	0.84	86.5
8	-0.05	1.18	36.6
9	0.93	0.64	97.5
10	1.04	0.49	96.0
11	-0.09	0.54	90.4
12	0.03	0.46	95.2
13	-0.01	0.44	92.5
14	-0.53	0.30	96.2
15	0.20	0.87	73.0
16	-2.08	0.75	93.2
17	-0.62	0.34	74.9

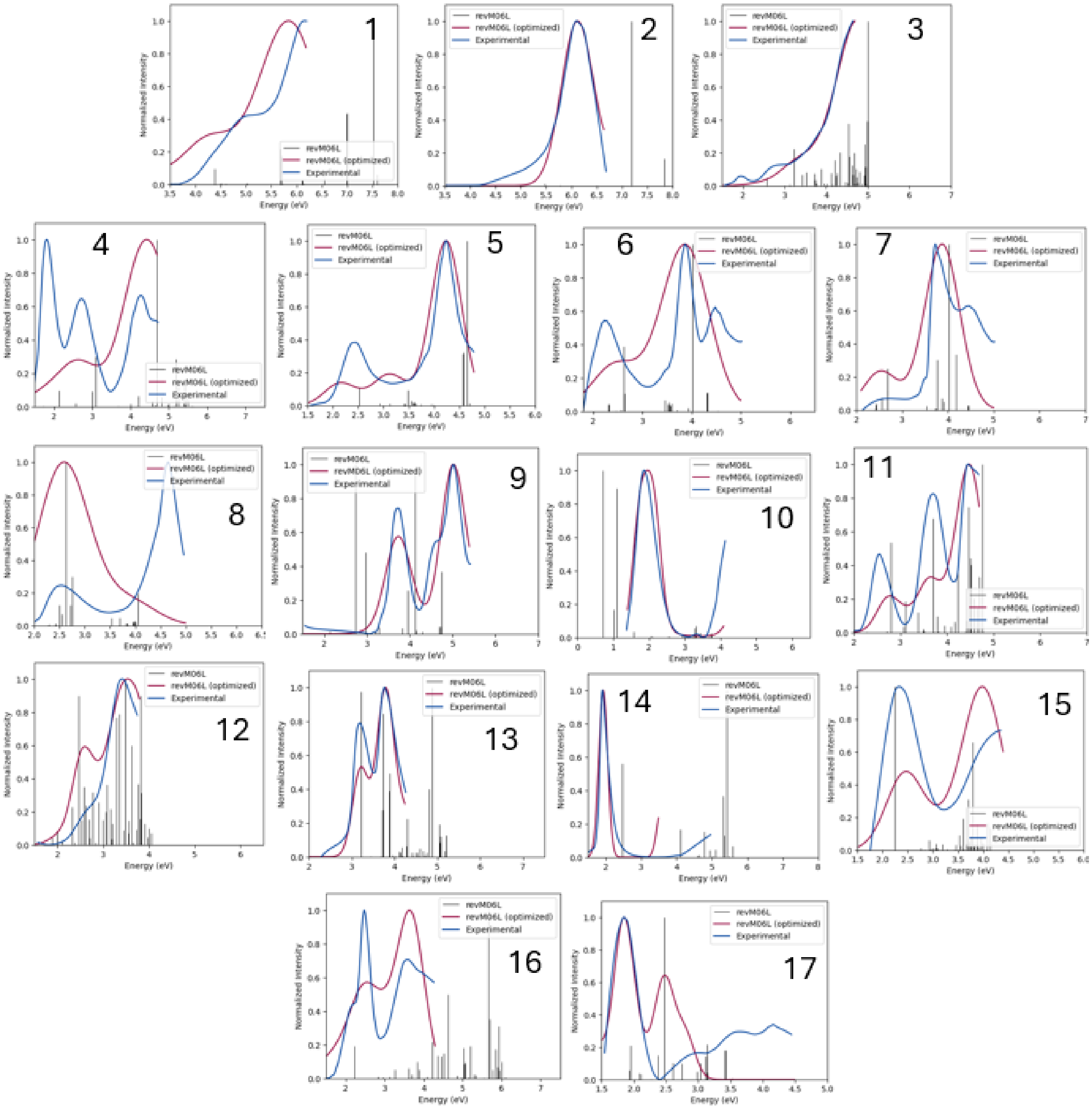

Figure S20: Calculated and optimized revM06L/def2-TZVP TD-DFT spectra for all compounds in comparison with experimental UV-Vis data. Labels correspond to the respective compound.

Table S58: Calculated errors for the TD-DFT spectra of all compounds obtained with the TPSSh/def2-TZVP method in comparison with experimental UV-Vis data.

**Compound**	shift (eV)	FWHM (eV)	sim (%)
1	-0.65	0.93	93.3
2	-0.76	0.79	99.5
3	-0.26	0.34	34.3
4	-0.35	0.67	73.2
5	-0.17	0.56	56.0
6	-0.07	1.18	82.7
7	0.01	0.99	99.4
8	-0.15	1.18	33.9
9	0.91	0.46	46.4
10	0.90	0.60	60.2
11	0.11	0.49	89.9
12	0.12	0.43	42.9
13	-0.09	0.46	92.2
14	-0.54	0.29	96.1
15	0.39	0.79	78.8
16	-1.93	1.18	90.0
17	0.00	0.30	72.2

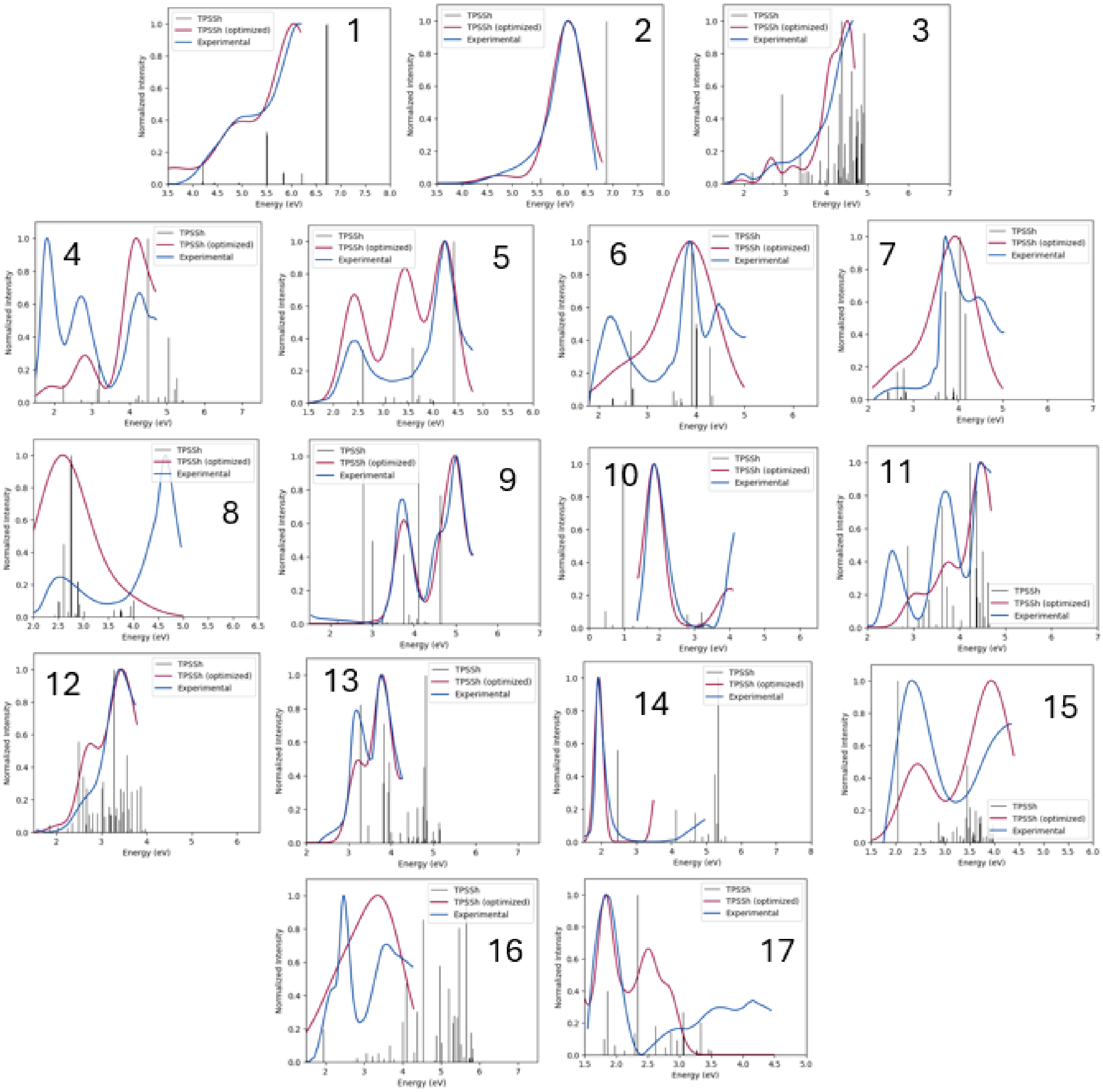

Figure S21: Calculated and optimized TPSSh/def2-TZVP TD-DFT spectra for all compounds in comparison with experimental UV-Vis data. Labels correspond to the respective compound.

Table S59: Calculated errors for the TD-DFT spectra of all compounds obtained with the O3LYP/def2-TZVP method in comparison with experimental UV-Vis data.

**Compound**	shift (eV)	FWHM (eV)	sim (%)
1	-0.56	0.92	99.2
2	0.71	0.72	99.0
3	-0.19	0.39	97.9
4	-0.29	0.63	74.1
5	-0.12	0.57	86.9
6	-0.03	1.18	82.3
7	0.03	1.01	88.8
8	-0.08	1.18	32.1
9	0.97	0.53	98.3
10	1.00	0.64	97.0
11	0.13	0.52	89.6
12	0.26	0.46	97.7
13	0.03	0.47	92.1
14	-0.48	0.29	96.2
15	0.43	0.78	72.1
16	-1.45	1.18	90.3
17	-0.34	0.34	74.9

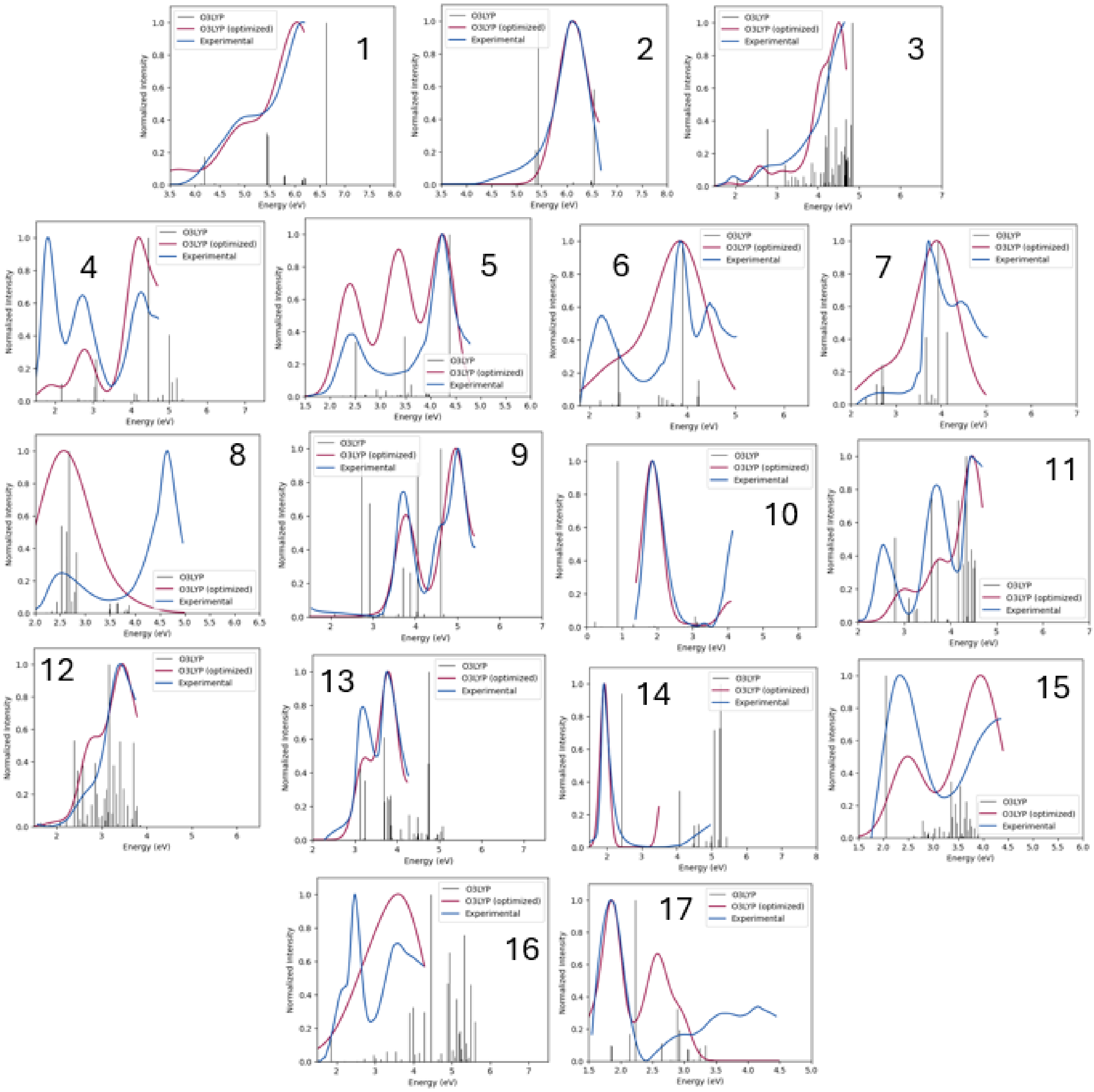

Figure S22: Calculated and optimized O3LYP/def2-TZVP TD-DFT spectra for all compounds in comparison with experimental UV-Vis data. Labels correspond to the respective compound.

Table S60: Calculated errors for the TD-DFT spectra of all compounds obtained with the B97/def2-TZVP method in comparison with experimental UV-Vis data.

**Compound**	shift (eV)	FWHM (eV)	sim (%)
1	-0.77	0.87	99.3
2	0.39	0.60	98.7
3	-0.34	0.93	99.1
4	-0.39	0.83	69.6
5	-0.25	0.58	96.1
6	-0.23	1.18	83.1
7	-0.09	0.81	93.0
8	-0.43	1.18	33.8
9	0.23	1.18	86.4
10	0.71	0.64	98.4
11	-0.05	0.44	90.0
12	-0.20	0.42	97.5
13	-0.51	0.35	91.1
14	-0.64	0.30	96.8
15	0.17	0.65	71.3
16	-1.76	0.76	92.6
17	-0.42	0.37	78.9

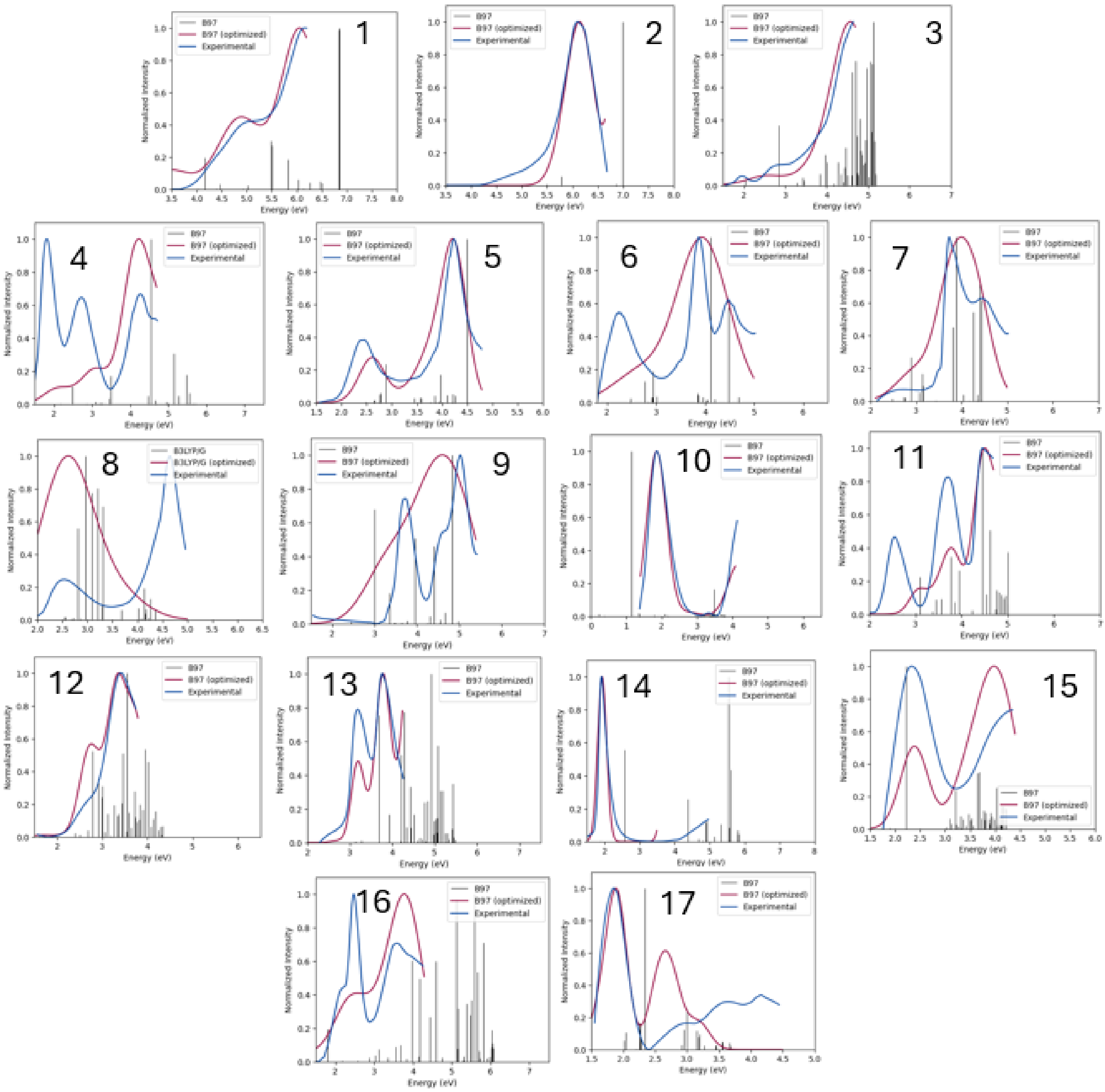

Figure S23: Calculated and optimized B97/def2-TZVP TD-DFT spectra for all compounds in comparison with experimental UV-Vis data. Labels correspond to the respective compound.

Table S61: Calculated errors for the TD-DFT spectra of all compounds obtained with the B3LYP(G)/def2-TZVP method in comparison with experimental UV-Vis data.

**Compound**	shift (eV)	FWHM (eV)	sim (%)
1	-0.74	0.85	99.3
2	0.40	0.60	98.6
3	-0.32	0.99	99.1
4	-0.37	1.18	68.9
5	-0.23	0.61	95.5
6	-0.23	1.18	82.8
7	-0.08	0.58	93.3
8	-0.47	1.18	33.9
9	0.24	1.06	87.2
10	0.64	0.62	98.1
11	-0.05	0.44	90.1
12	-0.22	0.44	97.7
13	-0.56	0.31	90.1
14	-0.66	0.30	96.8
15	0.20	0.64	71.8
16	-1.69	0.86	92.5
17	-0.42	0.38	77.3

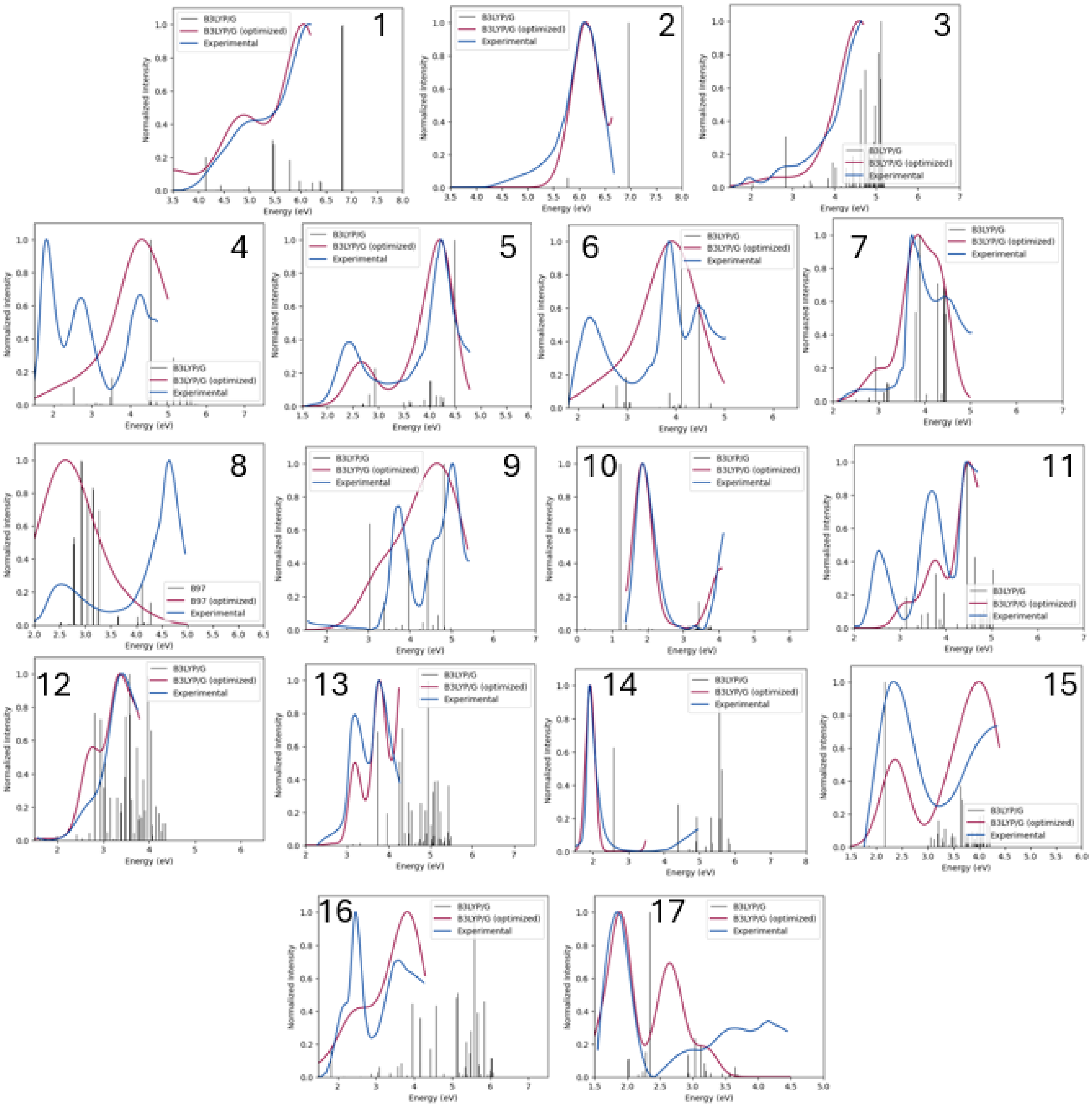

Figure S24: Calculated and optimized B3LYP(G)/def2-TZVP TD-DFT spectra for all compounds in comparison with experimental UV-Vis data. Labels correspond to the respective compound.

Table S62: Calculated errors for the TD-DFT spectra of all compounds obtained with the revM11/def2-TZVP method in comparison with experimental UV-Vis data.

**Compound**	shift (eV)	FWHM (eV)	sim (%)
1	-1.42	0.86	94.6
2	-0.59	0.63	98.5
3	-2.22	0.60	98.7
4	-0.91	1.18	63.2
5	-0.60	0.44	90.8
6	-0.78	0.41	83.2
7	-0.83	0.88	96.0
8	-0.67	0.45	94.7
9	-0.48	0.62	92.2
10	-3.06	1.18	56.6
11	-0.68	1.18	89.1
12	-0.45	0.66	98.3
13	0.71	0.59	91.0
14	-1.32	0.30	96.8
15	-1.12	1.18	66.5
16	-1.21	0.37	93.6
17	-0.60	0.60	74.2

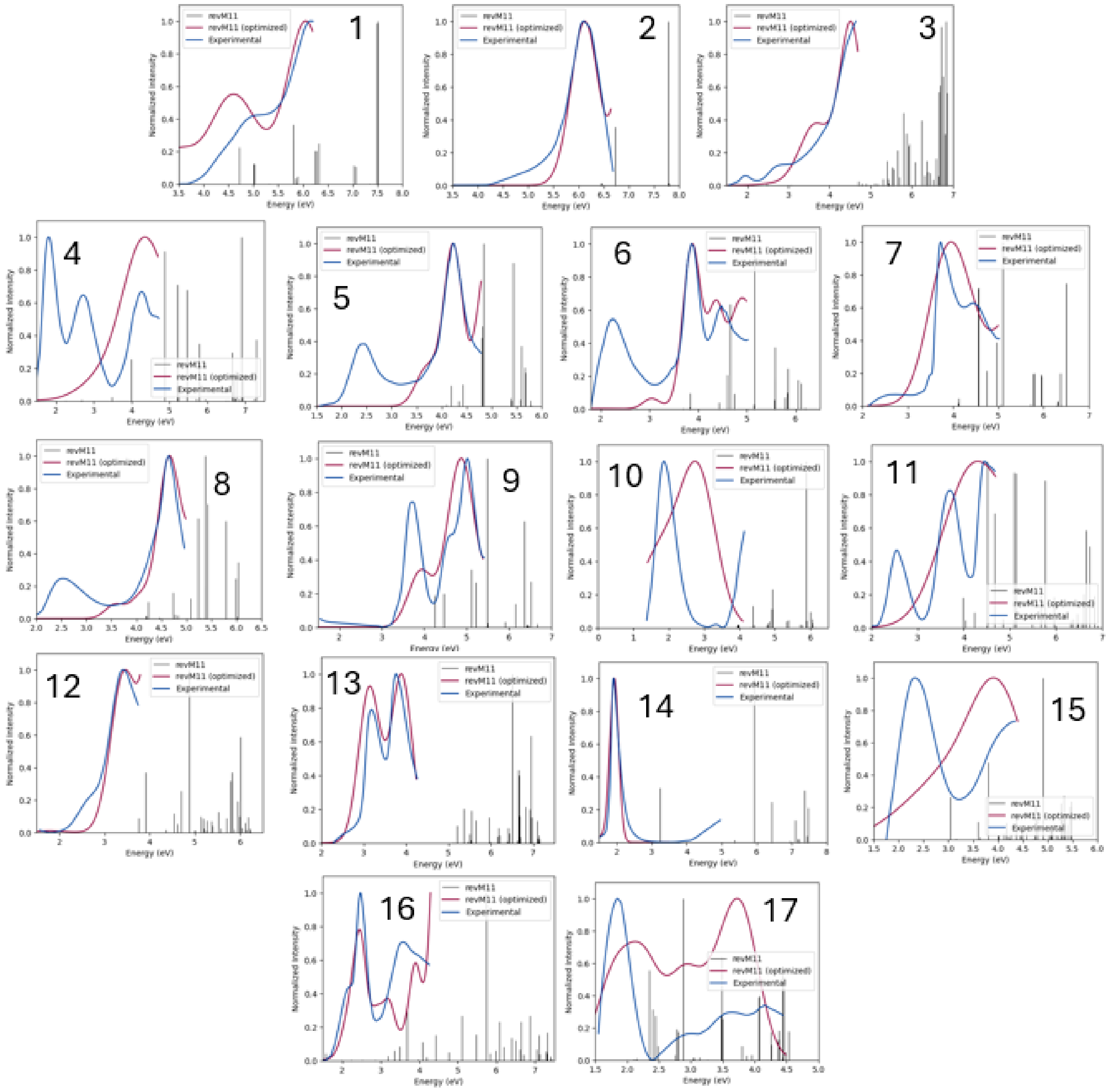

Figure S25: Calculated and optimized revM11/def2-TZVP TD-DFT spectra for all compounds in comparison with experimental UV-Vis data. Labels correspond to the respective compound.

Table S63: Calculated errors for the TD-DFT spectra of all compounds obtained with the PBE0/def2-TZVP method in comparison with experimental UV-Vis data.

**Compound**	shift (eV)	FWHM (eV)	sim (%)
1	-0.94	0.86	98.9
2	0.13	0.57	98.9
3	-0.77	0.69	97.7
4	-0.44	1.18	67.4
5	-0.32	0.70	95.1
6	-0.41	1.18	82.2
7	-0.21	0.49	95.1
8	0.29	1.18	49.5
9	0.85	0.63	89.1
10	0.35	0.63	98.3
11	-0.20	0.43	90.8
12	-0.37	0.44	98.1
13	-0.24	0.39	73.0
14	-0.77	0.30	96.9
15	-0.09	0.61	71.1
16	-1.80	1.18	92.2
17	-0.53	0.33	83.9

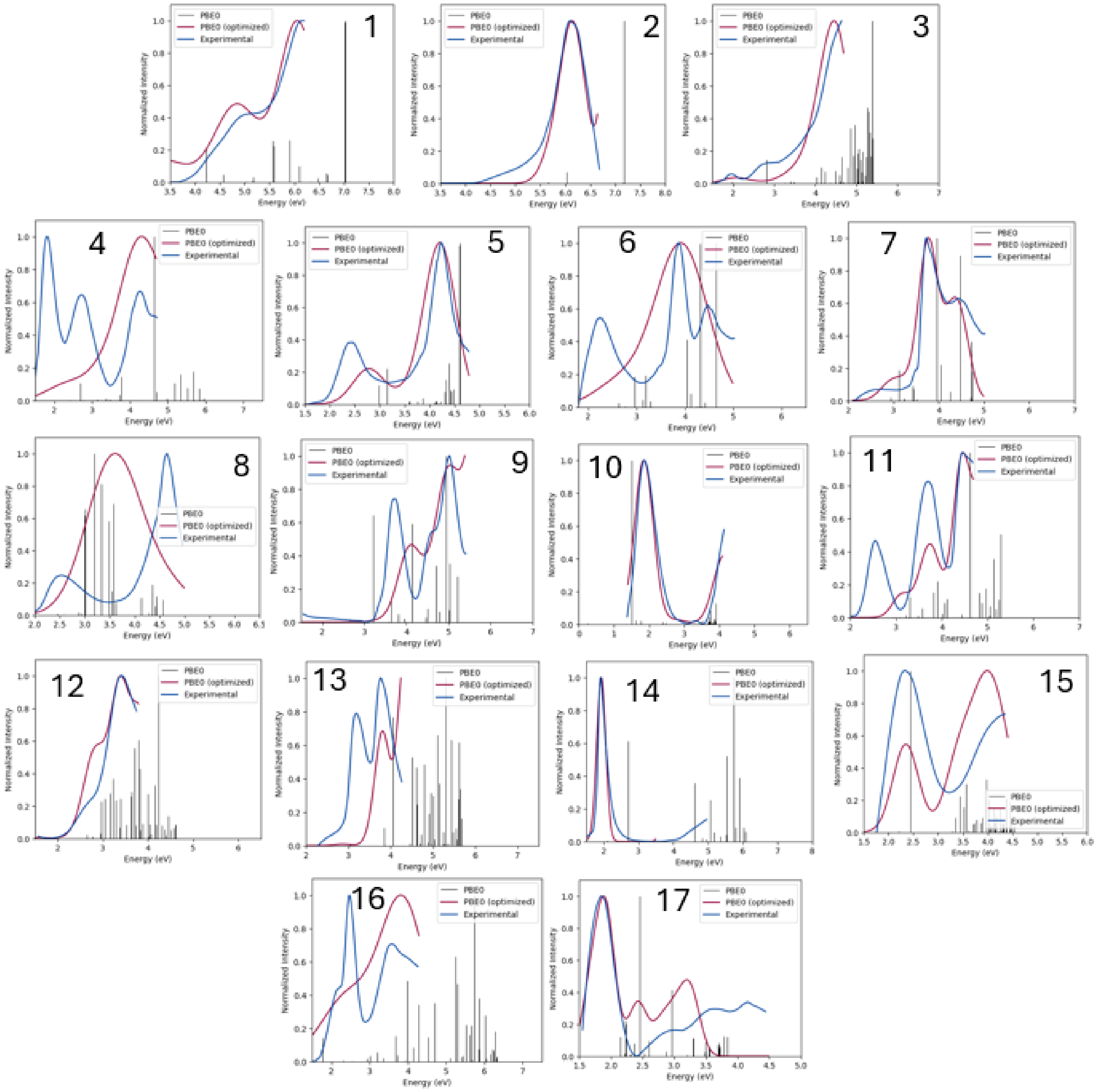

Figure S26: Calculated and optimized PBE0/def2-TZVP TD-DFT spectra for all compounds in comparison with experimental UV-Vis data. Labels correspond to the respective compound.

Table S64: Calculated errors for TD-DFT spectra of all compounds obtained with the MN15/def2-TZVP method in comparison with experimental UV-Vis data.

**Compound**	shift (eV)	FWHM (eV)	sim (%)
1	-0.95	0.93	99.2
2	0.04	0.70	98.8
3	-0.81	0.84	98.1
4	-0.46	1.18	64.8
5	-0.50	0.67	93.2
6	-0.34	1.18	82.8
7	-0.37	0.48	97.3
8	-0.15	0.52	82.8
9	-0.20	0.69	92.4
10	-0.44	0.73	71.8
11	-0.31	0.43	91.7
12	-0.31	0.79	99.2
13	1.23	0.50	92.5
14	-0.88	0.30	96.9
15	0.33	0.51	70.2
16	-1.10	1.18	88.2
17	-0.11	0.40	68.5

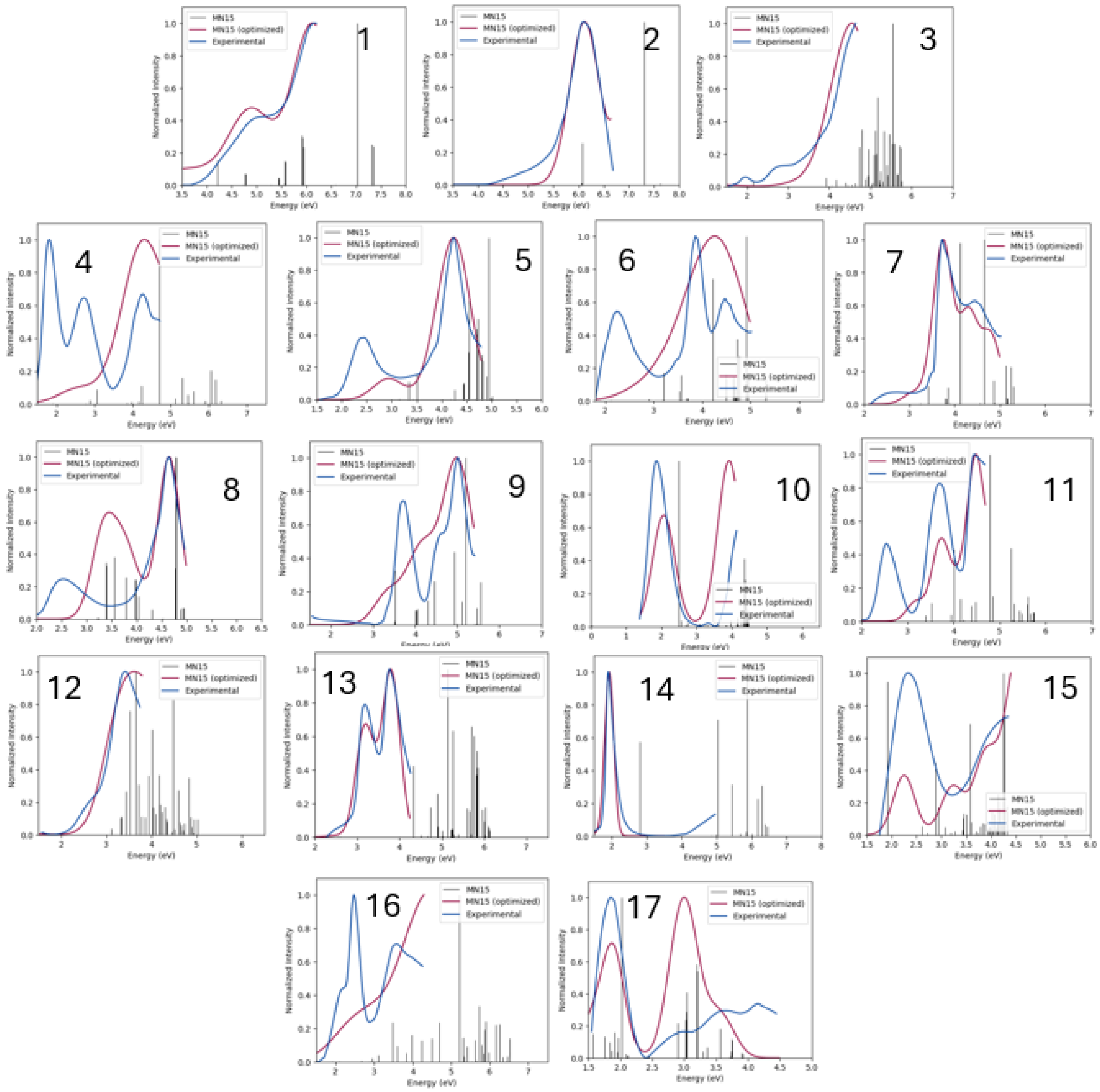

Figure S27: Calculated and optimized MN15/def2-TZVP TD-DFT spectra for all compounds in comparison with experimental UV-Vis data. Labels correspond to the respective compound.

Table S65: Calculated errors for TD-DFT spectra of all compounds obtained with the ωPBE/def2-TZVP method in comparison with experimental UV-Vis data.

**Compound**	shift (eV)	FWHM (eV)	sim (%)
1	-0.92	0.86	99.0
2	0.17	0.57	98.9
3	-0.64	0.66	97.7
4	-0.42	1.18	67.8
5	-0.30	0.68	95.0
6	-0.35	1.18	81.7
7	-0.15	0.49	93.8
8	-0.62	1.18	33.7
9	0.89	2.03	90.9
10	0.53	0.64	98.0
11	-0.72	1.18	88.1
12	-0.31	0.43	98.7
13	-0.14	0.35	72.5
14	-0.75	0.30	96.8
15	0.05	0.61	72.2
16	-1.77	0.98	92.4
17	-0.46	0.35	82.9

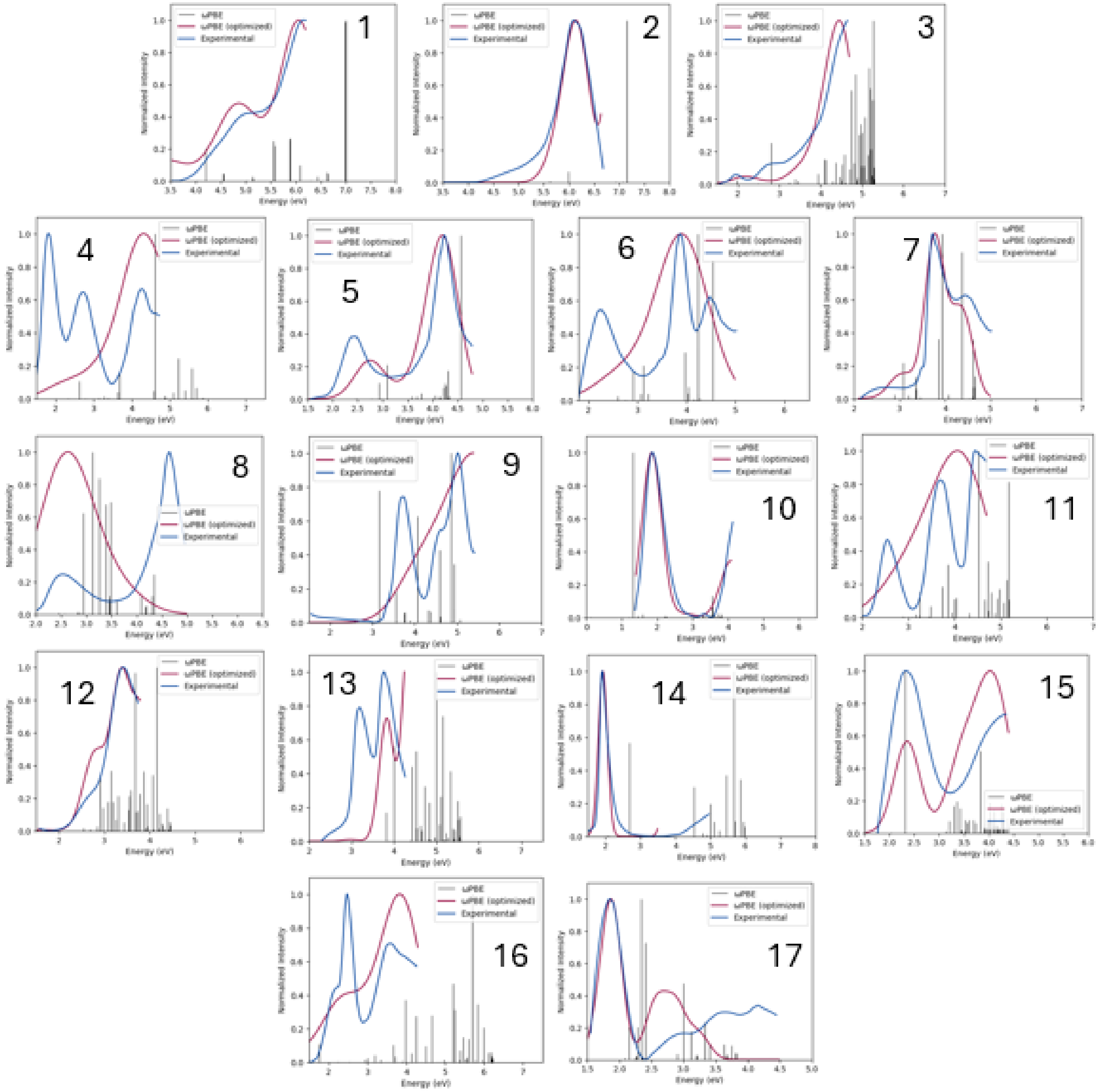

Figure S28: Calculated and optimized ωPBE/def2-TZVP TD-DFT spectra for all compounds in comparison with experimental UV-Vis data. Labels correspond to the respective compound.

Table S66: Calculated errors for TD-DFT spectra of all compounds obtained with the CAM-B3LYP/def2-TZVP method in comparison with experimental UV-Vis data.

**Compound**	shift (eV)	FWHM (eV)	sim (%)
1	-1.14	0.82	96.9
2	-0.15	0.57	98.3
3	-1.27	0.87	97.8
4	-0.59	1.18	64.5
5	-0.73	0.61	92.2
6	-0.52	1.18	82.6
7	-0.53	0.53	96.5
8	-0.65	0.57	95.7
9	-0.17	0.97	91.2
10	-0.61	0.60	59.9
11	-0.50	0.44	92.4
12	-0.64	0.71	98.8
13	0.07	0.40	73.2
14	-1.00	0.30	96.9
15	-0.37	1.18	66.3
16	-2.04	1.18	91.0
17	-0.69	0.36	84.2

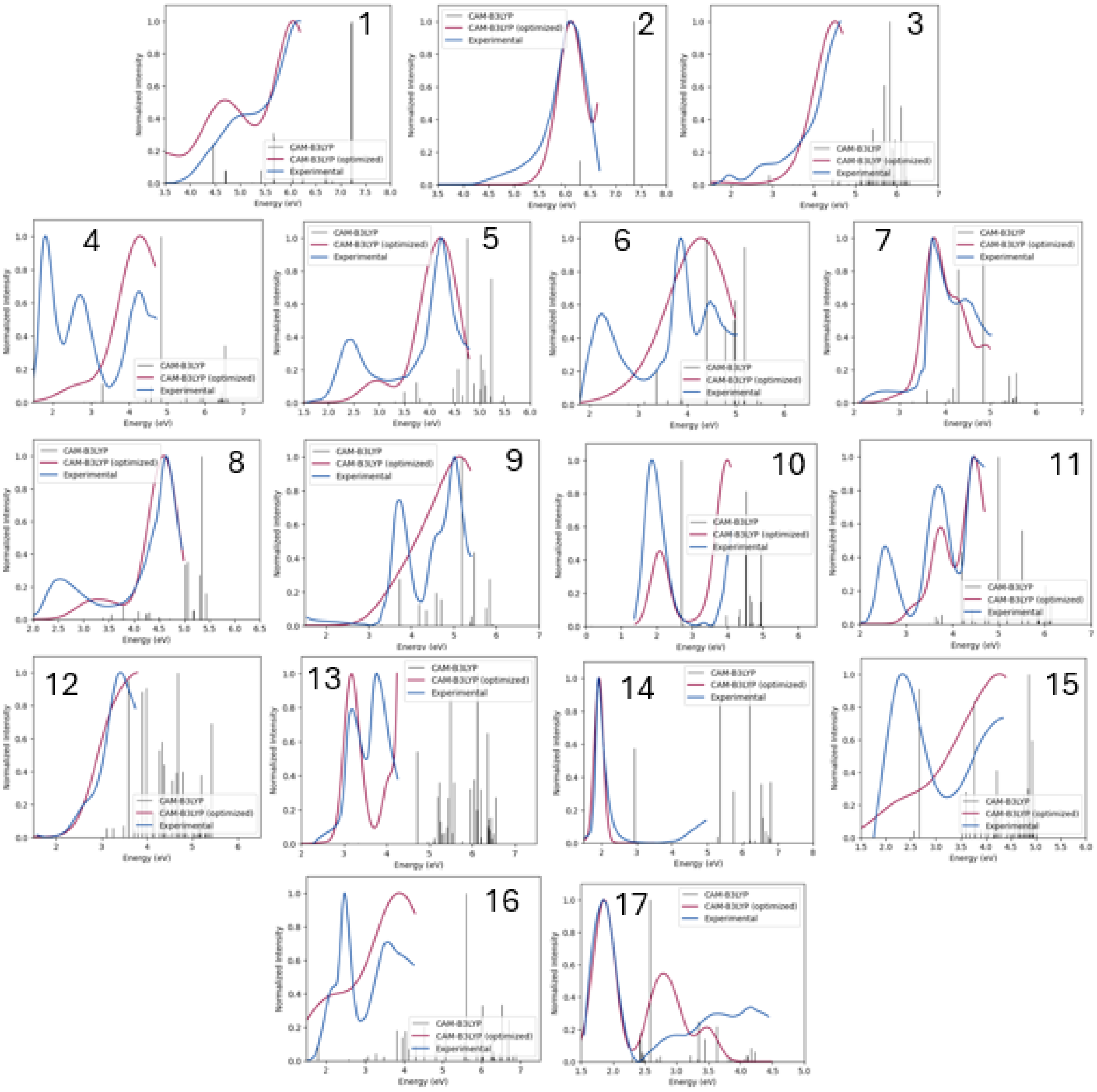

Figure S29: Calculated and optimized CAM-B3LYP/def2-TZVP TD-DFT spectra for all compounds in comparison with experimental UV-Vis data. Labels correspond to the respective compound.

Table S67: Calculated errors for the TD-DFT spectra of all compounds obtained with the ωB97X/def2-TZVP method in comparison with experimental UV-Vis data.

**Compound**	shift (eV)	FWHM (eV)	sim (%)
1	-1.30	0.89	95.3
2	-0.37	0.55	98.2
3	-2.10	0.48	96.2
4	-0.79	1.18	61.7
5	-0.55	0.37	85.5
6	-0.71	0.40	82.4
7	-0.74	0.82	95.7
8	-0.62	0.96	93.3
9	-0.39	0.43	93.8
10	-1.49	0.30	46.8
11	-0.70	0.43	91.9
12	-0.49	0.51	97.0
13	0.10	0.67	78.6
14	-1.11	0.30	96.9
15	-0.52	1.18	64.2
16	-1.61	0.38	91.1
17	-0.78	0.35	84.3

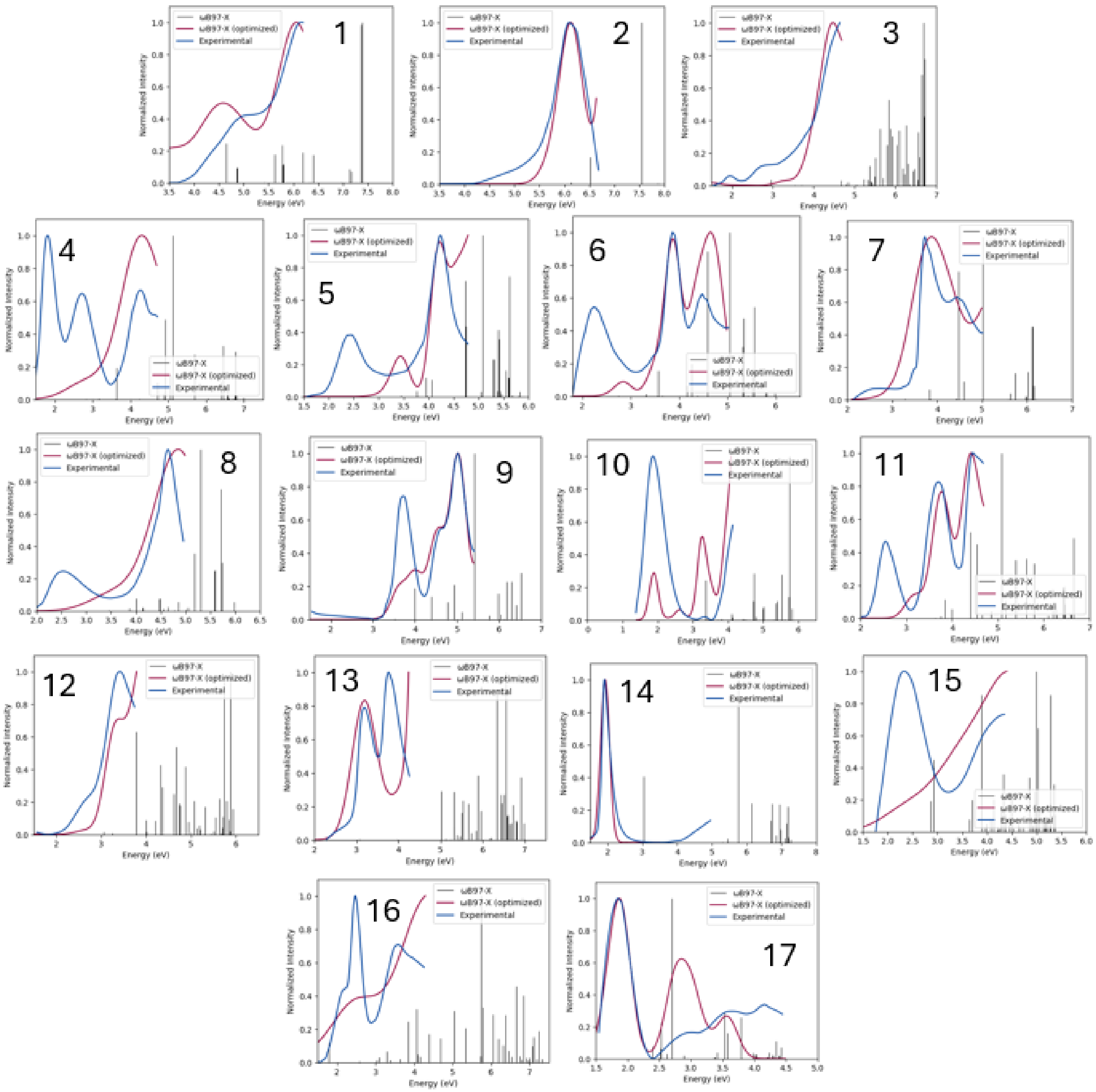

Figure S30: Calculated and optimized ωB97X/def2-TZVP TD-DFT spectra for all compounds in comparison with experimental UV-Vis data. Labels correspond to the respective compound.

Table S68: Average calculated errors for the TD-DFT spectra obtained with every method in comparison with experimental UV-Vis data.

**Method**	Shift (eV)	Absolute Shift (eV)	FWHM (eV)	sim (%)
TPSS	0.18	0.59	0.85	83.4
r^2SCAN	0.05	0.53	0.81	84.9
RevM06L	-0.19	0.52	0.74	87.1
TPSSh	-0.18	0.46	0.69	74.6
O3LYP	0.00	0.42	0.69	86.4
B97	-0.27	0.44	0.71	86.8
B3LYP/G	-0.27	0.44	0.72	86.7
RevM11	-0.95	1.04	0.71	86.9
PBE0	-0.32	0.51	0.69	87.0
MN15	-0.31	0.50	0.71	87.6
ωPBE	-0.35	0.54	0.80	85.8
CAM-B3LYP	-0.68	0.69	0.73	87.0
ωB97X	-0.83	0.84	0.60	85.5

References

(S1) Kotzian, M.; Roesch, N.; Schroeder, H.; Zerner, M. C. Optical spectra of transition-metal carbonyls: chromium hexacarbonyl, iron pentacarbonyl, and nickel tetracarbonyl. *J. Am. Chem. Soc.* **1989**, *111*, 7687–7696.

(S2) Scott, D. R.; Becker, R. S. Comprehensive Investigation of the Electronic Spectroscopy and Theoretical Treatments of Ferrocene and Nickelocene. *J. Chem. Phys.* **1961**, *35*, 516–531.

(S3) Scott, D. R.; Becker, R. S. Erratum: Comprehensive Investigation of the Electronic Spectroscopy and Theoretical Treatments of Ferrocene and Nickelocene. *J. Chem. Phys.* **1961**, *35*, 2246–2247.

(S4) Dunitz, J. D.; Orgel, L. E.; Rich, A. The crystal structure of ferrocene. *Acta Cryst.* **1956**, *9*, 373–375.

(S5) Pilon, A.; Gírio, P.; Nogueira, G.; Avecilla, F.; Adams, H.; Lorenzo, J.; Garcia, M. H.; Valente, A. New iron cyclopentadienyl complexes bearing different phosphane co-ligands: Structural factors vs. cytotoxicity. *J. Organomet. Chem.* **2017**, *852*, 34–42.

(S6) Liu, Y.; Kjaer, K. S.; Fredin, L. A.; Chábera, P.; Harlang, T.; Canton, S. E.; Lidin, S.; Zhang, J.; Lomoth, R.; Bergquist, K.-E.; Persson, P.; Wärnmark, K.; Sundström, V. A Heteroleptic Ferrous Complex with Mesoionic Bis(1,2,3-triazol-5-ylidene) Ligands: Taming the MLCT Excited State of Iron(II). *Chem. Eur. J.* **2015**, *21*, 3628–3639.

(S7) Nieuwenhuyzen, M.; Bertram, B.; Gallagher, J. F.; Vos, J. G. Dipotassium (2,2'-Bipyridyl-N,N')tetracyanoferrate(II)2.5-Hydrate, $K_2[Fe(bpy)(CN)_4] \cdot 2.5\,H_2O$. *Acta Cryst.* **1998**, *54*, 603–606.

(S8) Dick, S. Crystal structure of tris(2,2'-bipyridine)iron(II) bis(hexafluorophosphate), $(C_{10}H_8N_2)_3Fe(PF_6)_2$. *Z. fur Krist. - New Cryst. Struct.* **1998**, *213*, 370.

(S9) Vilà, N.; Walcarius, A. Bis(terpyridine) Iron(II) Functionalized Vertically-Oriented Nanostructured Silica Films: Toward Electrochromic Materials. *Front. Chem.* **2020**, *8*.

(S10) Baker, A.; Goodwin, H. Crystal Structure of Bis(2,2':6',2"-terpyridine)iron(II) Bis(perchlorate) Hydrate. *Aust. J. Chem.* **1985**, *38*, 207.

(S11) Fatur, S. M.; Shepard, S. G.; Higgins, R. F.; Shores, M. P.; Damrauer, N. H. A Synthetically Tunable System to Control MLCT Excited-State Lifetimes and Spin States in Iron(II) Polypyridines. *J. Am. Chem. Soc* **2017**, *139*, 4493–4505.

(S12) Germscheidt, R.; Morais, C.; Francischini, D.; Arruda, M. A.; Bonacin, J. Photochemical Pre-Treatment to Quantify Iron in Thin Films. *J. Braz. Chem. Soc.* **2023**, *34*, 958–966.

(S13) Mudasir; Yoshioka, N.; Inoue, H. Iron(II) and nickel(II) mixed-ligand complexes containing 1,10-phenanthroline and 4,7-diphenyl-1,10-phenanthroline. *Transit. Met. Chem.* **1999**, *24*, 210–217.

(S14) Kumar, K. S.; Giudice, N. D.; Heinrich, B.; Douce, L.; Ruben, M. Bistable spin-crossover in a new series of $[Fe(BPP-R)_2]^{2+}$ (BPP = 2,6-bis(pyrazol-1-yl)pyridine; R = CN) complexes. *Dalton Trans.* **2020**, *49*, 14258–14267.

(S15) Bohn, A.; Sénéchal-David, K.; Vanoutryve, J.; Guillot, R.; Rivière, E.; Banse, F. Synthesis and Characterization of Iron(II) Complexes with a BPMEN-Type Ligand Bearing π-Accepting Nitro Groups. *Eur. J. Inorg. Chem.* **2017**, *2017*, 3057–3063.

(S16) Santra, A.; Das, A.; Kaur, S.; Jain, P.; Ingole, P. P.; Paria, S. Catalytic reduction of oxygen to water by non-heme iron complexes: exploring the effect of the secondary coordination sphere proton exchanging site. *Chem. Sci.* **2024**, *15*, 4095–4105.

(S17) Atta, S.; Mandal, A.; Saha, R.; Majumdar, A. Reduction of nitrite to nitric oxide and generation of reactive chalcogen species by mononuclear Fe^{II} and Zn^{II} complexes of thiolate and selenolate. *Dalton Trans.* **2024**, *53*, 949–965.

(S18) Jackson, C. S.; Schmitt, S.; Dou, Q. P.; Kodanko, J. J. Synthesis, Characterization, and Reactivity of the Stable Iron Carbonyl Complex $[Fe(CO)(N_4Py)](ClO_4)_2$: Photoactivated Carbon Monoxide Release, Growth Inhibitory Activity, and Peptide Ligation. *Inorg. Chem.* **2011**, *50*, 5336–5338.

(S19) Malme, J. T.; Clendening, R. A.; Ash, R.; Curry, T.; Ren, T.; Vura-Weis, J. Nanosecond Metal-to-Ligand Charge-Transfer State in an Fe(II) Chromophore: Lifetime Enhancement via Nested Potentials. *J. Am. Chem. Soc* **2023**, *145*, 6029–6034.

(S20) Wang, H.; Cleary, M. B.; Lewis, L. C.; Bacon, J. W.; Caravan, P.; Shafaat, H. S.; Gale, E. M. Enzyme Control Over Ferric Iron Magnetostructural Properties. *Angew. Chem. Int. Ed.* **2022**, *61*.

(S21) Golovanov, I. S.; Leonov, A. V.; Lesnikov, V. K.; Pospelov, E. V.; Frolov, K. V.; Korlyukov, A. A.; Nelyubina, Y. V.; Novikov, V. V.; Sukhorukov, A. Y. Iron(IV) complexes with tetraazaadamantane-based ligands: synthesis, structure, applications in dioxygen activation and labeling of biomolecules. *Dalton Trans.* **2022**, *51*, 4284–4296.

(S22) Tomyn, S.; Shylin, S. I.; Bykov, D.; Ksenofontov, V.; Gumienna-Kontecka, E.; Bon, V.; Fritsky, I. O. Indefinitely stable iron(IV) cage complexes formed in water by air oxidation. *Nat. Commun.* **2017**, *8*, 14099.
